# KEAP1 promotes anti-tumor immunity by inhibiting PD-L1 expression in NSCLC

**DOI:** 10.1038/s41419-024-06563-3

**Published:** 2024-02-27

**Authors:** Jinghan Li, Daiwang Shi, Siyi Li, Xiang Shi, Yu Liu, Yi Zhang, Gebang Wang, Chenlei Zhang, Tian Xia, Hai-long Piao, Hong-Xu Liu

**Affiliations:** 1 Department of Thoracic Surgery, Cancer Hospital of China Medical University, Liaoning Cancer Hospital & Institute, Shenyang, 110042 China; 2 Dalian Institute of Chemical Physics, Chinese Academy of Sciences, Dalian, 116023 China; 3 Department of Thoracic Surgery, Shengjing Hospital, China Medical University, Shenyang, Liaoning China; 4 Department of Biochemistry & Molecular Biology, School of Life Sciences, China Medical University, Shenyang, 110122 China

**Keywords:** Non-small-cell lung cancer, Cancer immunotherapy, Ubiquitylation

## Abstract

Immunotherapy has become a prominent first-line cancer treatment strategy. In non-small cell lung cancer (NSCLC), the expression of PD-L1 induces an immuno-suppressive effect to protect cancer cells from immune elimination, which designates PD-L1 as an important target for immunotherapy. However, little is known about the regulation mechanism and the function of PD-L1 in lung cancer. In this study, we have discovered that KEAP1 serves as an E3 ligase to promote PD-L1 ubiquitination and degradation. We found that overexpression of KEAP1 suppressed tumor growth and promoted cytotoxic T-cell activation in vivo. These results indicate the important role of KEAP1 in anti-cancer immunity. Moreover, the combination of elevated KEAP1 expression with anti-PD-L1 immunotherapy resulted in a synergistic effect on both tumor growth and cytotoxic T-cell activation. Additionally, we found that the expressions of KEAP1 and PD-L1 were associated with NSCLC prognosis. In summary, our findings shed light on the mechanism of PD-L1 degradation and how NSCLC immune escape through KEAP1-PD-L1 signaling. Our results also suggest that KEAP1 agonist might be a potential clinical drug to boost anti-tumor immunity and improve immunotherapies in NSCLC.

## Introduction

Non-small cell lung cancer (NSCLC) comprises 85% of all lung cancer cases and remains a significant threat to human life, with high morbidity and mortality rates [1]. Despite the availability of treatment options for NSCLC, including radiotherapy, chemotherapy and targeted therapy [2], which have limited effectiveness due to drug resistance and harmful side effects [1, 3, 4]. Thus, the advent of immunotherapy, rooted in a deeper comprehension of tumor immunobiology, stands as a promising breakthrough in combating NSCLC [5].

Immunotherapy has become an emerging means of treating malignant tumors in recent years, which aims for reversing the immunosuppressive effect and evoking anticancer immune behaviors. Different from traditional chemoradiotherapy and targeted therapy, immunotherapy mainly kills tumor cells by activating the immune system [6, 7]. Immune checkpoint blockade (ICB) therapy is a type of immunotherapy, which functions mainly by blocking the recognition of the immune system to cancer cells. The transmembrane protein of programmed death ligand-1 (PD-L1) is an important ICB target for cancer treatment which is found to abnormally expressed on the surface of cancer cells [8, 9]. As a cell surface ligand, PD-L1 could interact with its receptor, programmed death protein-1 (PD-1), which is mainly expressed on the surface of T cells, to repress the activating of cytotoxic T cells, resulting in cancer cell evasion from immune surveillance [10, 11]. Despite ICBs therapies have demonstrated unprecedented clinical efficacy in the treatment of NSCLC [12] and greatly improved survival in some patients [13, 14]. it still should be noticed that most patients cannot benefit from these therapies [12, 15]. Recent studies indicate that the PD-L1 expression level in tumors were closely associated with the immunotherapy effect [16, 17]. Therefore, it is of great significance to investigate the regulation mechanism of PD-L1 and develop strategies to for improve the efficacy of immunotherapy.

Kelch-like ECH-associated protein 1 (KEAP1), which is an adaptor subunit of Cullin 3-based E3 ubiquitin ligase [18, 19], plays an important roles in diverse biological processes including metabolic reprogramming [20], ferroptosis [21], cell cycle [22] and radiation-therapy resistance [23]. Cancer genome sequencing studies have identified that KEAP1 is mutated in approximately 20% of both adenocarcinoma and squamous lung cancers [24, 25]. KEAP1 mutation is associated with cancer progression, treatment resistance, and poor patient survival in lung cancers [26–30]. Thus, KEAP1 plays an important role in the lung cancer progression, however, its relationship and molecular mechanism in immunotherapy is not fully understood.

In this study, we revealed previously undiscovered molecular mechanism of KEAP1 in inhibiting the NSCLC progression via mediating ICB. We found that KEAP1 directly interacts with PD-L1 and subsequently decreases PD-L1 expression by ubiquitinating PD-L1 for proteosome degradation. In turn, this degradation increased the number of CD8^+^ T cells, which enhance the effectiveness of immunotherapy. In addition, we found a negative correlation between KEAP1 and PD-L1 in clinical samples, and their different expressions were correlated with the prognosis. Collectively, our findings suggest that KEAP1 could serve as a potential target for the anti-PD-L1 immunotherapy to inhibit NSCLC progression.

## Results

### KEAP1 inhibits NSCLC cell growth and colony formation

**Fig. 1:**
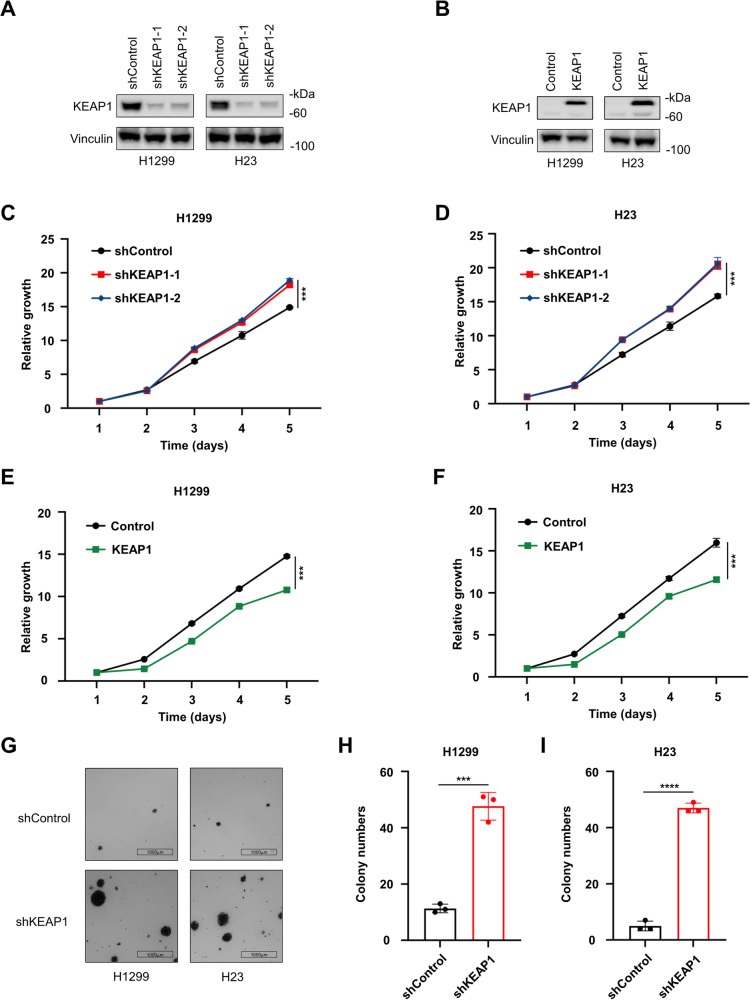
KEAP1 inhibits NSCLC cell growth and colony formation. **A** and **B** Expression of KEAP1 in KEAP1 knockdown or overexpression H1299 and H23 cells were analyzed by immunoblotting. **C** and **D** Cell growth curves of H1299 and H23 KEAP1 knockdown cells according to the CCK8 assays. **E** and **F** Cell growth curves of KEAP1 overexpression H1299 and H23 cells according to the CCK8 assays. **G**-**I** The colony formation assay of KEAP1 knockdown H1299 and H23 cells in soft agar. The colonies were represented by nitrotetrazolium blue chloride staining followed by capturing pictures (**G**). Then the colony numbers were calculated by Image J (**H, I**). *n* = 3 wells per group. Scale bar: 1000 μm. Data in **C**-**F**, **H**, **I** are presented as mean values ± SD. **C**-**F** are two-way ANOVA test. H, I are unpaired t test.

Initially, we used The Cancer Genome Atlas (TCGA) database to analyze the gene mutations of patients with lung adenocarcinoma (LUAD) and lung squamous cell carcinoma (LUSC), respectively. We found that the high mutation rates of KEAP1 ranked in both LUAD and LUSC (Figure S1A, B). To investigate the role of KEAP1 in lung cancer cells, we first determined KEAP1 protein levels in a panel of different lung cancer cell lines. KEAP1 was abundantly expressed in H1299 and H23 cells, while moderately expressed in other lung cancer cell lines (Figure S2A). To clarify the role of KEAP1 in the pathogenesis of lung cancer, we knock down of KEAP1 with two different short hairpin RNAs (shRNAs) in H1299, H23 and HCC827 cells (Fig. 1A, S2B). Additionally, we overexpressed exogenous KEAP1 fused with an SF (S and Flag) tag in H1299 and H23 cells (Fig. 1B). Knockdown of KEAP1 accelerated cell growth (Fig. 1C, D, S2C), while KEAP1 overexpression significantly decreased cell proliferation in these lines (Fig. 1E, F). Furthermore, KEAP1 knockdown notably increased cell colony formation ability in soft agar (Fig. 1G–I, S2D, E). In summary, these findings strongly suggest that KEAP1 serves as a tumor suppressor, inhibiting lung cancer cell growth and colony formation.

### KEAP1 inhibits cell growth and tumorigenesis by downregulation of PI3K-AKT signaling in NSCLC

**Fig. 2:**
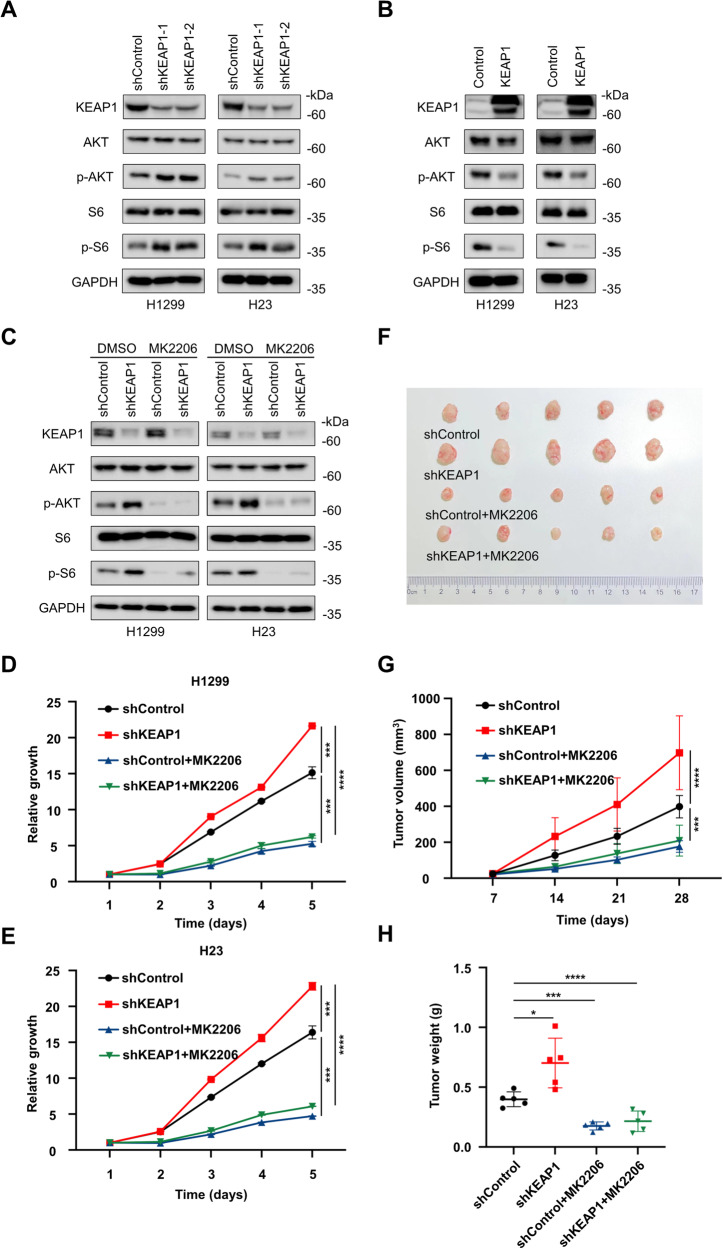
KEAP1 inhibits cell growth and tumorigenesis by downregulation of PI3K-AKT signaling in NSCLC. **A** and **B** Immunoblotting analysis of KEAP1, AKT, p-AKT, S6 and p-S6 in KEAP1 knockdown (**A**) and overexpression (**B**) H1299 and H23 cells. **C** KEAP1 knockdown H1299 and H23 cells were treated with 2.5 μM MK2206 for 24 hours and then the cells were lysed to test the levels of KEAP1, AKT, p-AKT, S6 and p-S6 by immunoblotting. **D** and **E** Cell growth curves of KEAP1 knockdown cells H1299 and H23 as well as their corresponding control groups with or without the treatment of 1 μM MK2206. **F**–**H** Tumor images (**F**), growth curve (**G**) and weight (**H**) of from mice injected subcutaneously with KEAP1 knockdown H1299 cells, along with control cells, treated with or without MK2206 were analyzed (*n* = 5). Data in **D**, **E**, **G** and **H** are presented as mean values ± SD. **D**, **E** and **G** are two-way ANOVA test. **H** is unpaired t test.

To gain further insights into the potential mechanism of KEAP1 in cancer progression, we conducted an initial analysis using Gene Set Enrichment Analysis (GSEA). Leveraging the KEGG database, we identified signaling pathways associated with KEAP1, with particular emphasis on the negative association with the PI3K-AKT signaling pathway, a widely recognized promoter of cancer (Figure S3A, B). In line with this finding, we observed a significant upregulation of Phospho-AKT (p-AKT) and its downstream target, Phospho-S6 Ribosomal Protein (p-S6), in KEAP1 knockdown cell lines compared to control cells (Fig. 2A, S3C). Conversely, KEAP1 overexpressed cells displayed decreased levels of p-AKT and p-S6 (Fig. 2B). To elucidate the functional role of KEAP1 in regulating the AKT signaling pathway, we treated KEAP1 knockdown cells with the specific AKT inhibitor MK2206 to neutralize the impact of KEAP1 on p-AKT levels (Fig. 2C). Furthermore, KEAP1 knockdown lung cancer cells exhibited heightened proliferation capability (Fig. 2D, E) and accelerated tumor development in nude-mice xenograft models (Fig. 2F–H). Importantly, these tumorigenic effects were abrogated in the presence of MK2206 (Fig. 2D–H). These results collectively indicate that KEAP1 hinders NSCLC cell proliferation by negatively regulating the PI3K-AKT signaling pathway both in vivo and in vitro.

### KEAP1 directly interacts with PD-L1

**Fig. 3:**
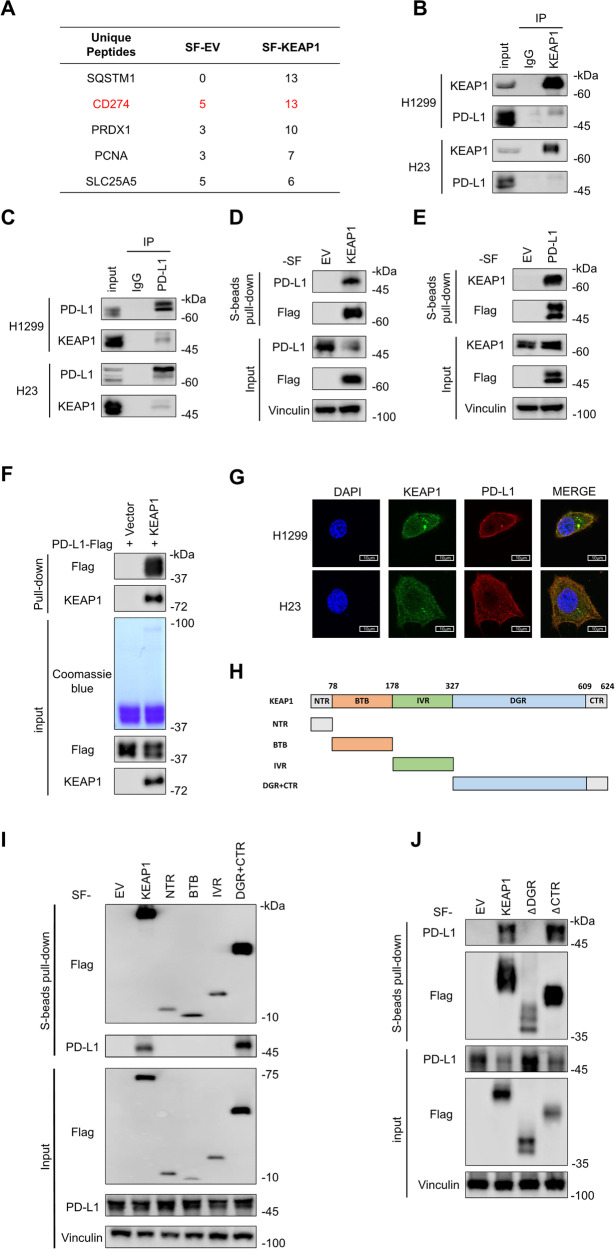
KEAP1 directly interacts with PD-L1. **A** HEK293T cells were transfected with SF-EV (empty vector with SF-tag) and SF-KEAP1 (KEAP1 with SF-tag), then cell lysates were pulled down with S-beads and analyzed by mass spectrometry. The protein names and corresponding peptide numbers were listed in the column. **B** and **C** Cell lysates of H1299 and H23 were immunoprecipitated with IgG, KEAP1 or PD-L1 antibodies, and endogenous interactions of KEAP1 and PD-L1 were tested by immunoblotting. **D** and **E** HEK293T cells were transfected with SF-KEAP1 and PD-L1-SF (PD-L1 tagged with SF), respectively. Cell lysates were pulled down with S-beads, and immunoblotting was performed using PD-L1 or KEAP1 antibodies to test the interaction of KEAP1 and PD-L1. **F** GST pull-down assay with purified GST-KEAP1 and PD-L1-Flag, and immunoblotting was performed to assess the interaction of KEAP1 and PD-L1. **G** Immunofluorescence (IF) staining for KEAP1 (green), PD-L1 (red), and nuclei (DAPI, blue) was performed in H1299 and H23 cells. Scale bars, 10 μm. **H–J** SF-tag full-length and truncated KEAP1 constructs were transfected into HEK293T cells, lysates were pulled down with S-beads to elucidate the interacting domain of KEAP1 to PD-L1.

To further gain insight into the underlying anti-tumor mechanism of KEAP1, we performed mass spectrometry (MS) based protein interaction analysis in HEK293T cells to determine the KEAP1 interacting proteins. The MS results highlighted PD-L1 (CD274) as one of the robustly interacting proteins with KEAP1 (Fig. 3A). Next, the association of KEAP1 and PD-L1 was verified by mutually immunoprecipitation assays using endogenous KEAP1 and PD-L1 in H1299 and H23 cells (Fig. 3B, C). Moreover, the specific interaction between exogenously transduced KEAP1 and PD-L1 was confirmed through co-precipitation assays in HEK293T cells (Fig. 3D, E). To investigate whether there is a direct interaction between KEAP1 and PD-L1, we conducted pulldown assay by using purified GST-KEAP1 and PD-L1-Flag proteins, and found that clearly interacted with each other (Fig. 3F). This interaction was further substantiated by immunofluorescence assays, indicating co-localization of KEAP1 with PD-L1 predominantly in the membrane and cytoplasmic regions (Fig. 3G).

To obtain more comprehensive understanding of the interaction, full-length and various truncated fragments of KEAP1, each fused with an SF-tag, were transduced in HEK293T cells (Fig. 3H). Consequently, it was determined that the double glycine region (DGR) and the C-terminal region (CTR) of KEAP1 were instrumental in associating with PD-L1 (Fig. 3I). Further investigation, involving separate deletions of the DGR and CTR regions, pinpointed the DGR as the crucial region binding to PD-L1 (Fig. 3J). In summary, our findings identify PD-L1 as a novel interaction protein with KEAP1, and further investigations are warranted to elucidate the intricacies of their regulatory relationship.

### KEAP1 ubiquitinates and induces PD-L1 degradation through 26S-proteosome route

According to previous reports, PD-L1 degradation via both lysosomal and proteasomal pathways. KEAP1 is well-known to target NRF2 for ubiquitination and degradation. In this study, we have demonstrated an interaction between PD-L1 and KEAP1 at the DGR domain, which raise the speculation that PD-L1 may be a new substrate of KEAP1. To test this hypothesis, we first investigated the expression levels of PD-L1 in both KEAP1-knockdown and overexpressing cell lines, with NRF2 as a control. The results indicated that KEAP1 exerts a negative regulatory effect on PD-L1, like that on NRF2 (Figure S4A, B). Besides, PD-L1 expression exhibit negative correlation with KEAP1 in NSCLC cells (Figure S2A). In addition, analysis of RNA levels in KEAP1-knockdown H1299 cells revealed no discernible impact between KEAP1 and PD-L1 transcript level (Figure S4C). Moreover, treatment of KEAP1-knockdown H1299 cells with the NRF2 inhibitor ML385 did not alter the protein expression level of PD-L1 or the KEAP1 downstream responder p-AKT. (Figure S4D). Collectively, these results suggest that KEAP1 negatively regulates PD-L1 protein level.

**Fig. 4:**
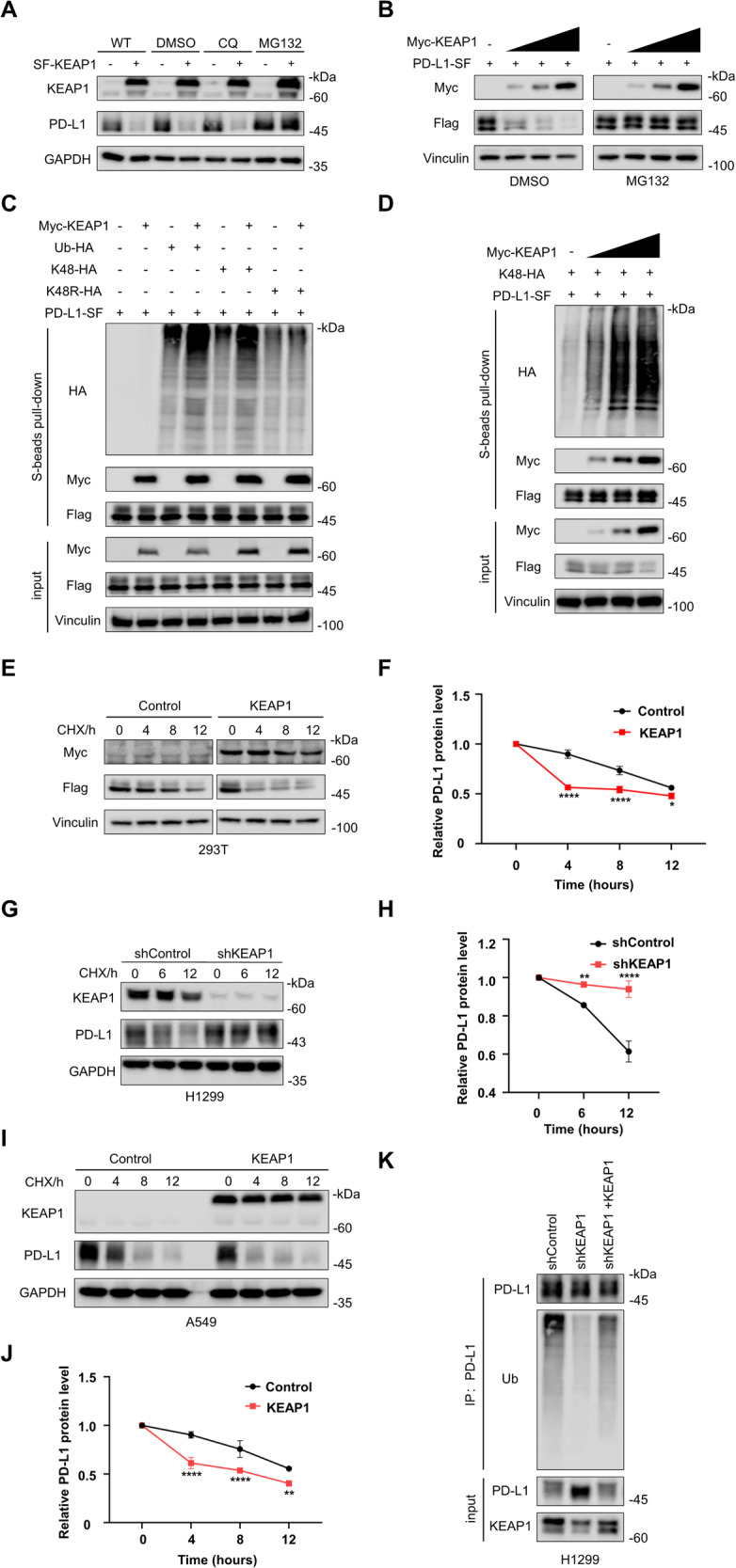
KEAP1 ubiquitinates PD-L1 and induces PD-L1 degradation through 26S-proteosome route. **A** HEK293T cells were transfected with SF-KEAP1 and treated with DMSO, CQ and MG132 respectively, then the cells were lysed to test the level of KEAP1 and PD-L1. **B** HEK293T cells were transfected with Myc-KEAP1 (Myc-tagged KEAP1) and PD-L1-SF, following with the treatment of 10 μM MG132 for 6 hours. Immunoblotting with Myc and Flag antibodies. **C** Ubiquitination assay of PD-L1 in HEK293T cells co-transfected with Myc-KEAP1, PD-L1-SF, Ub-HA, or K48-HA and K48R-HA with the treatment of 10 μM MG132 for 6 hours. Then the cells were lysed and pulled-down with S-protein agarose, and the indicated protein levels were tested by immunoblotting. **D** Ubiquitination assay of PD-L1 in HEK293T cells co-transfected with PD-L1-SF, and K48-HA, along with varying amounts of Myc-KEAP1, followed by being treated with 10 μM MG132 for 6 hours. Then the cells were lysed and pulled-down with S-protein agarose, the indicated protein levels were tested by immunoblotting. **E** and **F** HEK293T cells were co-transfected with Myc-KEAP1, PD-L1-SF, and treated with 40 mΜ cycloheximide (CHX) for indicated time points. Then immunoblotting was performed to test the protein levels in cell lysate (**E**) and the protein levels were quantified according to the grey values (**F**). **G** and **H** shControl and shKEAP1 H1299 cells were treated with 40 mΜ cycloheximide (CHX) for indicated time points. Then immunoblotting was performed to test the protein levels in cell lysate (**G**) and the protein levels were quantified according to the grey values (**H**). **I** and **J** Control and KEAP1overexprssion A549 cells were treated with 40 mΜ cycloheximide (CHX) for indicated time points. Then immunoblotting was performed to test the protein levels in cell lysate (**I**) and the protein levels were quantified according to the grey values (**J**). **K** Endogenous ubiquitination assay of PD-L1 in shControl, shKEAP1 and shKEAP1 + KEAP1 H1299 cells. Cell lysates were immunoprecipitated with PD-L1 antibody, and endogenous PD-L1 ubiquitination was tested by immunoblotting. Data in **F**, **H** and **J** are presented as mean values ± SD. Two-way ANOVA test.

To investigate whether PD-L1 serves as a substrate for ubiquitination by KEAP1, we transduced SF-tagged KEAP1 alone (Fig. 4A) or Myc-tagged KEAP1 and SF-tagged PD-L1 together (Fig. 4B) into HEK293T cells. Remarkably, the upregulation of KEAP1 levels correlated with a significant reduction in both endogenous (Fig. 4A) and exogenous (Fig. 4B) PD-L1 protein, suggesting that KEAP1 inhibits the expression of PD-L1. Additionally, we observed that this decline of PD-L1 expression caused by KEAP1 was completely reversed in the presence of the proteasomal inhibitor MG132, but not the lysosomal inhibitor chloroquine (CQ) (Fig. 4A). Similar results were obtained when H1299 cells with KEAP1 knockdown or overexpression were treated with MG132 (Figure S5A, B), confirming that KEAP1 regulates PD-L1 expression through the proteasomal pathway.

Next, to investigate the possibility that KEAP1 ubiquitinates PD-L1, we performed PD-L1 ubiquitination assay by transducing KEAP1 in HEK293T cells. The results showed that KEAP1 enhanced the ubiquitination level of PD-L1 mainly through K48-linked manner, as the ubiquitination level was not altered when K48R mutated ubiquitin was used (Fig. 4C). In addition, we found KEAP1 promoted PD-L1 ubiquitination in a dose-dependent manner (Fig. 4D). Consistently, exogenously expression of KEAP1 in HEK293T cells accelerated PD-L1 reduction in the presence of the protein synthesis inhibitor cycloheximide (CHX) (Fig. 4E, F). This result was further validated in H1299 KEAP1 knockdown cells (Fig. 4G, H), providing additional evidence that KEAP1 facilitates PD-L1 degradation. In addition, we overexpressed KEAP1 in A549 cells, which harbors a KEAP1 mutation. The cycloheximide (CHX) assay demonstrated that KEAP1 overexpression in A549 cells also prompted a rapid reduction in PD-L1 levels (Fig. 4I, J). Subsequently, we rescued KEAP1 in KEAP1-knockdown H1299 cells and conducted endogenous ubiquitination assays. The results illustrated that the depletion of KEAP1 led to a reduction in the ubiquitination levels of PD-L1, which was restored upon KEAP1 reintroduction (Fig. 4K).

KEAP1 functions as an adaptor subunit of the Cullin3 (CUL3) E3 ubiquitin ligase complex. However, it remains to be determined whether CUL3 directly participates in the ubiquitination process of PD-L1. To address this, we generated CUL3 knockdown cell lines in H1299, observing a subsequent increase in PD-L1 expression (Figure S5C). Furthermore, treatment with MG132 significantly inhibited the degradation of PD-L1 (Figure S5D). Subsequently, in HEK293T cells with CUL3 knockdown, we conducted exogenous ubiquitination assays by transfecting Myc-KEAP1, PD-L1-SF, and K48-HA. The results revealed that upon CUL3 knockdown, the K48-ubiquitination levels of PD-L1 by KEAP1 decreased (Figure S5E). Additionally, the CHX assay indicated a significant slowdown in the degradation of PD-L1 after CUL3 knockdown (Figure S5F, G). These findings collectively suggest that KEAP1 can reduce the protein stability of PD-L1 by targeting PD-L1 for ubiquitination, with CUL3 playing a crucial role in this regulatory mechanism.

### KEAP1 regulates the PI3K-AKT signaling pathway through PD-L1 to inhibit cell growth in vivo and in vitro

**Fig. 5:**
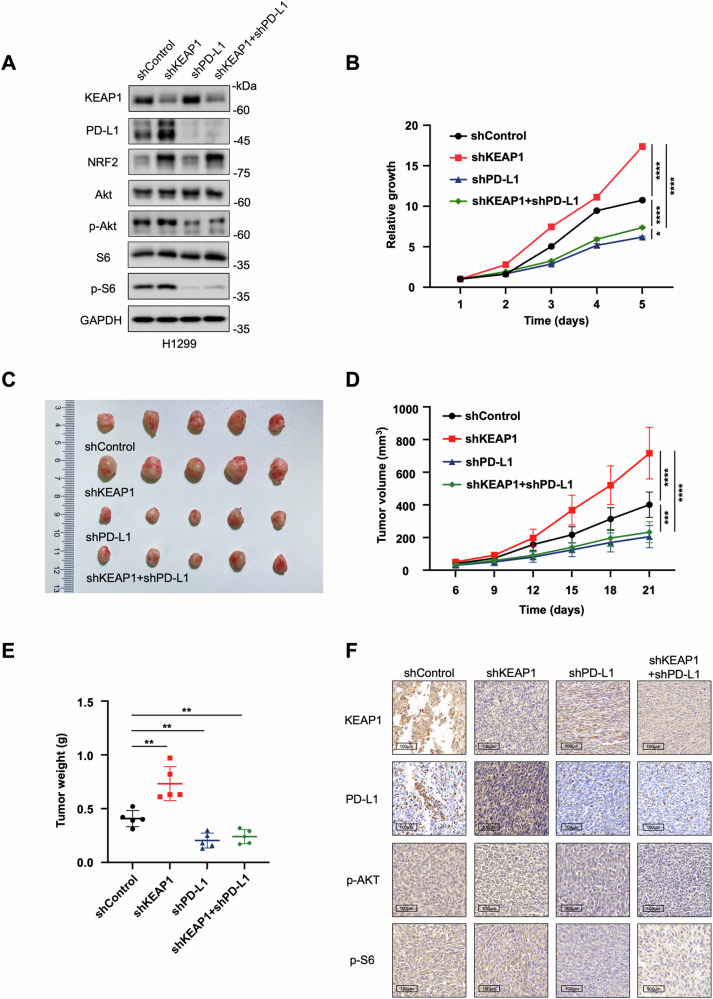
KEAP1 regulates the PI3K-AKT signaling pathway through PD-L1 to inhibit cell growth in vivo and in vitro. **A** Immunoblotting analysis of KEAP1, PD-L1, NRF2, AKT, p-AKT, S6 and p-S6 in shControl, shKEAP1, shPD-L1 and shKEAP1+shPD-L1 H1299 cells. **B** Cell growth curves of shControl, shKEAP1, shPD-L1 and shKEAP1+ shPD-L1 H1299 cells according to the CCK8 assays. **C**–**E** shControl, shKEAP1, shPD-L1 and shKEAP1+ shPD-L1 H1299 cells were injected into the nude mice, respectively. Tumor volumes were measured every 3 days. Tumor image (**C**), growth curves (**D**) and weight (**E**) were obtained at day 21 after injection (*n* = 5). **F** Representative IHC staining images in randomly selected tumors derived from (**C**). Scale bar: 100 µm. Data in **B**, **D** and **E** are presented as mean values ± SD. B and D are two-way ANOVA test. E is unpaired t test.

In order to assess the interplay between KEAP1 and PD-L1 in cancer cell proliferation and tumorigenesis, we established cell lines with PD-L1 knockdown and those with simultaneous knockdown of both KEAP1 and PD-L1 in H1299. Interestingly, we observed a decrease in the levels of p-Akt and p-S6 upon PD-L1 downregulation (Fig. 5A). Notably, the proliferation rate of cells decreased following PD-L1 knockdown, and this effect counteracted the accelerated proliferation caused by KEAP1 knockdown (Fig. 5B). This observation suggested an epistatic relationship of PD-L1 to KEAP1 in modulating cell proliferation.

To further assess tumorigenesis, we subcutaneously injected these cells into nude mice. As illustrated in Fig. 5C–E, knockdown of KEAP1 led to enhanced tumor development, whereas the introduction of PD-L1-targeting shRNA significantly mitigated the tumorigenic tendency. Additionally, our findings were consistent with previous results indicating that KEAP1 suppresses AKT signaling (Fig. 2A–C). Additionally, the tumors resulting from KEAP1 knockdown exhibited increased AKT activation (Fig. 5F), which was interestingly attenuated in the KEAP1 and PD-L1 double knockdown tumors (Fig. 5F), indicating that KEAP1 represses AKT signaling through PD-L1. Collectively, these results highlighted the role of KEAP1 in regulating the PI3K-AKT signaling pathway via PD-L1, thereby exerting inhibitory effects on NSCLC cell growth in vitro and in vivo.

### KEAP1 promotes anti-tumor immunity via PD-L1 degradation

**Fig. 6:**
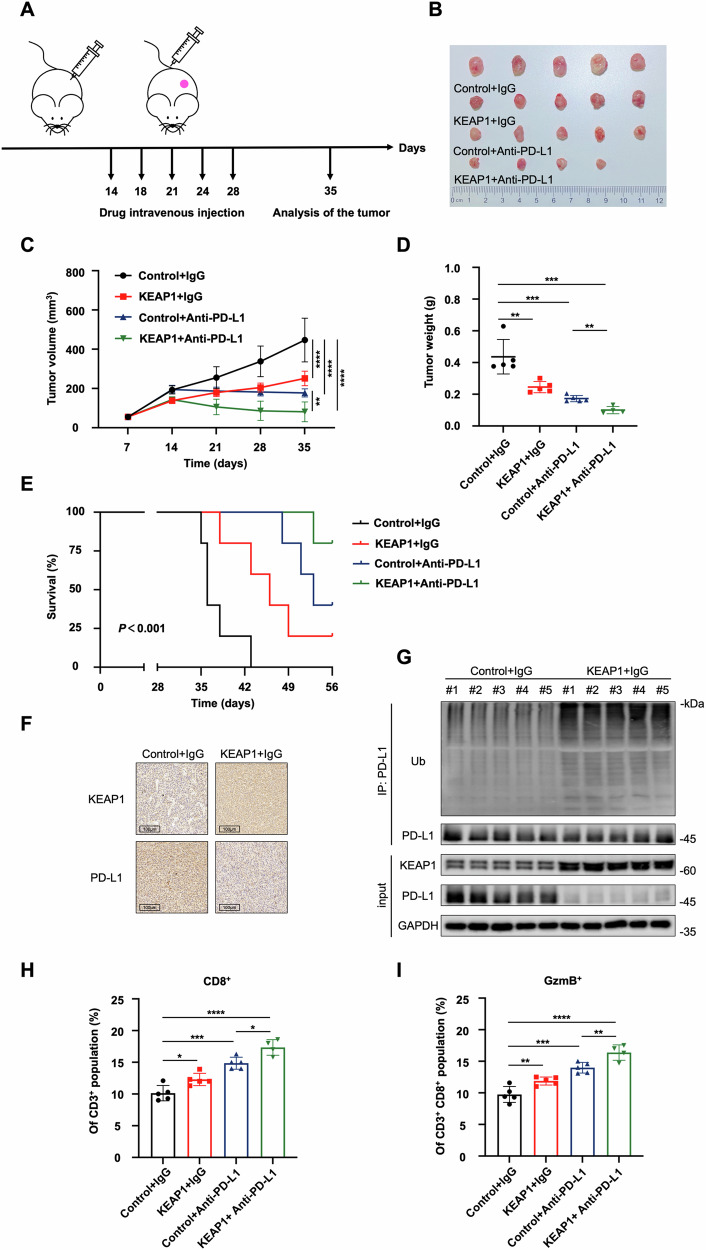
KEAP1 promotes anti-tumor immunity via inducing PD-L1 degradation. **A** The schematic of the in vivo anti-tumor effect analysis of KEAP1. **B**–**D** LLC KEAP1 overexpression cells and the corresponding control cells were injected into C57BL/6 mice. IgG or anti-PD-L1 were injected intravenously after inoculation of LLC cells. Tumor volumes were measured every 7 days. Tumor images (**B**) growth curves (**C**) and weight (**D**) were obtained at day 35 after inoculation (*n* = 4-5). **E** Kaplan–Meier curves of the survival analysis of the mice with indicated treatments, *n* = 5 mice per group. **F** Representative IHC staining images in randomly selected tumors from mice in IgG treatment group. Scale bar: 100 µm. **G** The dissected tumors from IgG treatment group were lysed and followed by immunoblotting to test the indicated protein levels. **H** and **I** Flow cytometry analysis for the levels of CD3^+^ CD8^+^ T cells (**F**) and CD3^+^CD8^+^ GzmB^+^ T cells (**G**) (*n* = 4-5) in different groups of tumors. Data in **C**, **D**, **H**, and **I** are presented as mean values ± SD. **C** is two-way ANOVA test. D, H, and I are unpaired t test.

Our findings robustly established that KEAP1 ubiquitinates PD-L1 for degradation (Fig. 4), influencing tumor progression (Fig. 5). Given the significance of PD-L1 as a target for tumor immunotherapy, we aimed to investigate the potential involvement of KEAP1 in tumor immunity. To address this, we stably expressed KEAP1 in mouse Lewis lung carcinoma (LLC) cells, followed by their subcutaneous injection into C57BL/6 mice (Figure S3A). After 14 days implantation, we administered IgG or anti-PD-L1 into the mice (Fig. 6A). Consistent with the observations in human lung cancer cells, the overexpression of KEAP1 in LLC cells led to inhibited tumor growth (Fig. 6B–D) and prolonged survival (Fig. 6E) compared to the control cells. In line with the uncovered regulatory mechanism in this study, the overexpression of KEAP1 resulted in increased ubiquitination of PD-L1 (Fig. 6G) and a significant reduction in PD-L1 protein level (Fig. 6F, G).

As expected, the reduction in PD-L1 expression in KEAP1-overexpressing cells led to an upregulation of tumor immunity, evidenced by elevated levels of total and activated CD8^+^ cytotoxic T cells (GzmB^+^) within tumor-infiltrating lymphocytes (Fig. 6H, I and Figure S3B). This was accompanied by elevated expressions of inflammatory cytokines and T-cell chemokines, including tumor necrosis factor-α (TNF-α), interferon-γ (IFN-γ), T-cell chemokines C-C motif chemokine ligand 5 (CCL-5), and C-X-C motif chemokine ligand 10 (CXCL-10) (Figure S3C-F). Remarkably, the administration of anti-PD-L1 significantly suppressed tumor progression (Fig. 6B–D), resulting in prolonged survival (Fig. 6E) and activated tumor immune response (Fig. 6H, I). Furthermore, the combination of KEAP1 overexpression and anti-PD-L1 treatment further enhanced the therapeutic outcome (Fig. 6B–D, H, I, and S3C–F). Taken together, our data provided compelling evidence that KEAP1 enhances the efficacy of anti-PD-L1 tumor immunotherapy, suggesting that KEAP1 may hold promise as a potential treatment target.

### Clinical implication of KEAP1 as a potential target for tumor immunotherapy

**Fig. 7:**
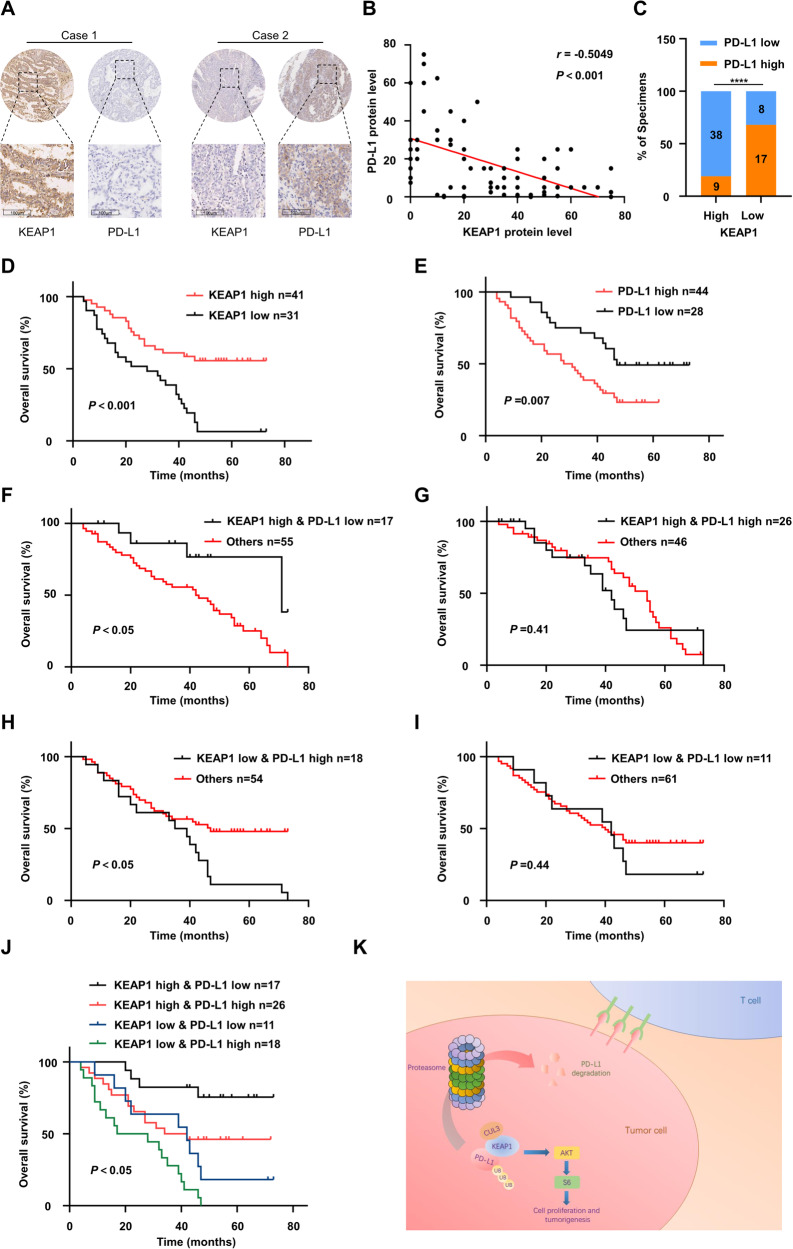
Clinical implication of KEAP1 as a potential target in tumor immunotherapy. **A** Representative IHC staining images of KEAP1 and PD-L1 in TMAs (HLugA180Su04, contains prognosis information, Shanghai Outdo Biotech Company). Scale bar: 100 µm. **B** Correlation analysis between KEAP1 and PD-L1 protein expression based on H-Score in NSCLC TMAs (HLugA180Su04). The *r* values and *P* values are obtained by Pearson’s correlation analysis. **C** Percentages of specimens exhibiting high or low KEAP1 expression were correlated with PD-L1 levels. Chi-square test. **D** and **E** Kaplan–Meier curves of the survival analysis of patients with high and low expression levels of KEAP1 (**D**) and PD-L1 (**E**) based on TMAs prognosis data (HLugA180Su04). **F**–**J** Kaplan–Meier curves of the survival analysis of KEAP1 high & PD-L1 low (**F)**, KEAP1 high & PD-L1 high (**G**), KEAP1 low & PD-L1 high (**H**), and KEAP1 low & PD-L1 low (**I**) with the rest samples respectively and all four groups (**J**) of patients based on TMAs prognosis data (HLugA180Su04). **K** A schematic displaying the working model of KEAP1 regulating PD-L1.

To elucidate the clinical potential of KEAP1-mediated PD-L1 expression, we conducted IHC staining and analysis on tissue microarrays (TMAs) comprising lung cancer and corresponding adjacent normal tissues from non-small cell lung cancer (NSCLC) patients (Figure S7). As depicted in Fig. 7A and B, a significant negative correlation was evident between the levels of KEAP1 and PD-L1 based on the IHC staining. Subsequently, we categorized the samples into two groups based on the expression of KEAP1, revealing distinct patterns of PD-L1 expression (Fig. 7C). In this context, the majority of individuals in the KEAP1-high group exhibited low PD-L1 expression, while samples with low PD-L1 expression accounted for approximately two-thirds of the KEAP1-high group (Fig. 7C).

Moreover, we conducted an analysis of clinical follow-up information from 72 NSCLC patients to validate the impact of KEAP1 and PD-L1 on overall survival. Our results confirmed that high level of KEAP1 was associated with prolonged overall survival in NSCLC (Fig. 7D), while PD-L1 exhibited the opposite effect (Fig. 7E). Subsequently, we divided the patient population into four distinct groups based on the expression levels of KEAP1 and PD-L1, and compared their overall survival with the remaining three groups (Fig. 7F–J). Notably, patients with high KEAP1 and low PD-L1 expression displayed significantly extended survival time (Fig. 7F), whereas those with low KEAP1 and high PD-L1 expression had a poorer prognosis (Fig. 7H). Conversely, there was no significant difference in survival between the other two groups characterized by either high or low expression levels of both KEAP1 and PD-L1 compared to the remaining groups (Fig. 7G, I).

Collectively, these findings suggested a negative correlation between KEAP1 and PD-L1 in NSCLC tumorigenesis, and the expression of KEAP1 closely associated patient prognosis. Given the regulatory role of KEAP1 in PD-L1 degradation, enhancing the expression or activity of KEAP1 may provide potential benefits for PD-L1-targeted immunotherapy in NSCLC.

## Discussion

This study uncovered a novel KEAP1 anti-tumor mechanism in NSCLC by orchestrating the degradation of PD-L1 (Figs. 4 and 5). Specifically, the downregulation of KEAP1 activates PI3K-AKT signaling pathway in NSCLC cells through PD-L1 (Fig. 7K). Conversely, the downregulated PD-L1 expression by KEAP1 increases CD8^+^ T cells, thereby promoting anti-tumor immunity (Fig. 6H, I). TMA analysis confirmed the negative correlation between KEAP1 and PD-L1 expression (Fig. 7A, B), while patients with high KEAP1 and low PD-L1 expression associated with better prognosis in NSCLC (Fig. 7F–G). Therefore, these results suggest that KEAP1 agonists could enhance the effectiveness of PD-L1 antibodies and improve therapeutic outcomes.

Most cells depend on KEAP1 to facilitate the degradation of nuclear factor erythroid 2-related factor 2 (NRF2) via the proteasome. When subjected to oxidative stress, KEAP1 becomes inactive, allowing NRF2 to accumulate and translocate into the nucleus, thereby stimulating the expression of genes responsible for detoxification and antioxidant defense [19]. While p62 physically interacts with KEAP1 to competitively release NRF2 and promoted NRF2 translocation to nucleus [31]. Under oxidative stress conditions, NRF2 expression increased, while KEAP1 and p62 expression decreased. However, their interactions with KEAP1 changed differently, while the interaction of KEAP to PD-L1 was upregulated, the binding to p62 decreased (Figure S8), suggesting a potential competitive interaction of PD-L1 and p62 to KEAP1. Although we have proved that KEAP1 regulated PD-L1 independently of NRF2, these clues indicated a possible regulatory function of p62 to PD-L1, especially in ROS, which needs more evidences in the future study. Furthermore, the dysregulation of PD-L1, coupled with reduced KEAP1-mediated regulation, could contribute to tumor progression and poor survival outcomes. Thus, understanding the impact of KEAP1 modulation on PD-L1 expression is significant for the development and efficacy of therapeutic approaches targeting both oxidative stress and immune checkpoints in cancer treatment. Previous research indicates that the CUL3-KEAP1 complex regulates the ubiquitination of NRF2 and modulates the transcription of PD-L1 [32]. Consequently, inhibiting NRF2 function could potentially downregulate the protein level of PD-L1. In addition, patients with low NRF2 expression demonstrate a better prognosis after anti-PD-L1 therapy [33]. Our study identified a novel mechanism that KEAP1 interacts with PD-L1 and induces PD-L1 K48 ubiquitination and the following degradation, independently of NRF2. Taken together, our findings suggest that KEAP1 modulates PD-L1 level through ubiquitination, and providing more evidences for the application of KEAP1 in the treatment of NSCLC.

Research on the function of KEAP1 in immunotherapy is ongoing, but results remain inconclusive. A recent study found that patients with KEAP1 mutations had lower overall survival (OS) and progress-free survival (PFS) after anti-PD-1/PD-L1 treatment compared to patients with wild-type KEAP1 [29]. This discrepancy may be attributed to the association of KEAP1 mutations with lower immune cell infiltration and diminished activity in immune-related pathways, crucial for effective immunotherapy [34]. CB839 (Telaglenastat), a potent glutaminase inhibitor, is being investigated in phase II trials for advanced NSCLC patients with KEAP1 mutations [35, 36]. However, a different study revealed that combining CB839 with anti-PD-1 treatment inhibited the clonal expansion and activation of CD8 + T cells in KEAP1 and STK11 double-mutant lung cancer, resulting in a loss of therapeutic benefit [37]. Therefore, the effect of single-agent or combination therapy for patients with KEAP1 mutations requires further investigation.

Our results showed that high KEAP1 expression combined with anti-PD-L1 is associated with better therapeutic outcomes and prognosis, due to the increase of total and activated CD8^+^ cytotoxic T cells (GzmB^+^) induced by KEAP1 (Fig. 6A–E and H-I). Additionally, a prognostic analysis based on different expression levels of KEAP1 and PD-L1 revealed that patients with high KEAP1 expression and low PD-L1 expression had the best prognosis, while those with low KEAP1 and high PD-L1 expression exhibited the worst OS. These findings suggest that combination therapy with a KEAP1 agonist along with anti-PD-1/PD-L1 may offer enhanced treatment outcomes, and KEAP1 could potentially serve as a predictive biomarker for anti-PD-1/PD-L1 therapy.

In summary, our study identifies KEAP1 as a pivotal regulator of PD-L1, holding important implications for anti-tumor immunity. We have demonstrated that KEAP1 reduces PD-L1 stability by facilitating ubiquitin-mediated degradation, leading to the inhibition of tumor growth both in vivo and in vitro. Importantly, KEAP1 overexpression enhances the anti-tumor therapeutic efficiency in mice. Furthermore, our findings reveale a negative correlation between KEAP1 and PD-L1 expression level, highlighting the potential of KEAP1 as a prognostic biomarker. Although no KEAP1 agonists are currently in clinical development or application, our results underscore KEAP1 as a promising target for cancer immune checkpoint blockade therapy. Future preclinical studies are warranted to delve into its mechanisms and explore potential targeting drugs.

## Materials and Methods

### Cell lines and culture conditions

The NSCLC cell lines and LLC cell were purchased from the cell bank of Committee on Type Culture Collection of the Chinese Academy of Sciences (CTCC, Shanghai, China). HEK293T cells were obtained from ATCC (American Type Culture Collection) and cultured under conditions specified by the provider. The NSCLC cell lines were maintained in RPMI 1640 medium (GIBCO, USA). HEK293T and LLC cells were cultured in DMEM medium (GIBCO, USA). The medium was mixed with 10% FBS (GIBCO, USA) and 1% penicillin/streptomycin (ThermoFisher). All cells were cultured at 37°C with 5% CO_2_ in a humidified incubator.

### Reagents and antibody

The compounds and their sources are as follows: MK-2206 (#S1078, Selleck), ML385 (#S8790, Selleck), DMSO (#PWL064, Meilun), Chloroquine (#S6999, Selleck), MG132(#S2619, Selleck), Cycloheximide (#S7418, Selleck), anti-PD-L1 (#A2004, Selleck), Human IgG1 kappa, Isotype Control (#HY-P99001, MCE), purified GST-KEAP1 protein (#HY-P75899, MCE), purified PD-L1-Flag protein (#HY-P70663, MCE), Coomassie Blue Staining Solution (#P0017F, Beyotime), Reactive Oxygen Species Assay Kit (#S0033S, Beyotime). The following antibodies were used in this study: CD3 (#12-0037-42, eBioscience), CD8 (#MA5-28792, eBioscience), Granzyme B (#17-8898-82, eBioscience), KEAP1 (#4678, Cell signaling Technology, 1:1000), KEAP1 (#sc-365626, Santa Cruz Biotechnology, 1:1000), KEAP1 (#10503-2-AP, Proteintech, 1:3000), PD-L1 (#13684, Cell signaling Technology, 1:1000), PD-L1 (#17952-1-AP, Proteintech, 1:5000), NRF2 (#12721, Cell signaling Technology, 1:1000), NRF2 (#16396-1-AP, Proteintech, 1:2000), CUL3 (#11107-1-AP, Proteintech, 1:2000), SQSTM1/p62 (#88588, Cell signaling Technology, 1:1000), AKT (#60203-2-Ig, Proteintech, 1:3000), Phospho-AKT (Ser473) (#4060, Cell signaling Technology, 1:1000), Ribosomal Protein S6 (#sc-74459, Santa Cruz Biotechnology, 1:1000), Phospho-S6 Ribosomal Protein (Ser235/236) (#4858, Cell signaling Technology, 1:1000), Vinculin (#sc-73614, Santa Cruz Biotechnology, 1:1000), GAPDH (#60004-1-Ig, Proteintech, 1:3000), Flag-tag (#F2555, Sigma-Aldrich, 1:1000), Myc-tag (#16286-1-AP, Proteintech, 1:3000), HA-tag (#66006-2-Ig, Proteintech, 1:2000), Ubiquitin (#10201-2-AP, Proteintech, 1:2000), Goat anti-Mouse IgG (H + L), Alexa Fluor™ 488 (#A-11001, ThermoFisher), Donkey anti-Rabbit IgG (H + L), Alexa Fluor™ 555 (#A-31572, ThermoFisher).

### Vector construction

Full-length and truncated KEAP1 coding sequences, as well as PD-L1 sequences were amplified by PCR and then subcloned to pCDNA3.1 or pBOBI vector with SF (S and Flag) tag or Myc tag in appropriate restriction endonuclease sites.

For the shRNAs (short hairpin RNA) in cells, lentivirus packaging system was used. Lentiviral shRNAs were cloned in pLKO.1 within the AgeI/EcoRI sites at the 3’end of the human U6 promoter. The shRNA sequences sequences were as follows:

hKEAP1-1

F 5’-CCGGGCGAATGATCACAGCAATGAACTCGAGTTCATTGCTGTGATCATTCGCTTTTTTG -3’

R 5’-AATTCAAAAAAGCACTGCAAATAACCCATCTTCTCGAGAAGATGGGTTATTTGCAGTGC-3’

hKEAP1-2

F 5’-CCGGGCACTGCAAATAACCCATCTTCTCGAGAAGATGGGTTATTTGCAGTGCTTTTTTG-3’

R 5’-AATTCAAAAAAGCACTGCAAATAACCCATCTTCTCGAGAAGATGGGTTATTTGCAGTGC-3’

hCUL3-1

F 5’- CCGGGACTATATCCAGGGCTTATTGCTCGAGCAATAAGCCCTGGATATAGTCTTTTTTG-3’

R 5’-AATTCAAAAAAGACTATATCCAGGGCTTATTGCTCGAGCAATAAGCCCTGGATATAGTC -3’

hCUL3-2

F 5’- CCGGCGTGTGCCAAATGGTTTGAAACTCGAGTTTCAAACCATTTGGCACACGTTTTTTG -3’

R 5’- AATTCAAAAAACGTGTGCCAAATGGTTTGAAACTCGAGTTTCAAACCATTTGGCACACG -3’

hPD-L1

F 5’- CCGGAATGAAGAAAGATGGAGTCAACTCGAGTTGACTCCATCTTTCTTCATTTTTTTG -3’

R 5’- AATTCAAAAAAATGAAGAAAGATGGAGTCAACTCGAGTTGACTCCATCTTTCTTCATT -3’

### Transfection and lentivirus infection

HEK293T cells were used for transfection and lentivirus packaging. Plasmids and PEI (Polyethylenimine, Polysciences 24765) were mixed at 1:4 ratio (w/w) in Opti-MEM medium (Gibco), and then added to HEK293T cell medium. Protein expression was tested about 48 hours after transfection.

For lentivirus packaging, the protein or shRNA expressing lentivirus vectors were transfected into HEK293T cells together with packaging vectors psPAX2 and pVSVG using PEI. Virus containing medium was harvested 48 hours after transfection and filtered with 0.45 μm membrane to remove cell debris, then the virus-containing medium was used to culture target cells for 8-24 hours. Puromycin or blasticidine were used to screen positive cells, depending on the lentivirus vector-resistant gene.

### Immunoblotting assay

Cells were scraped from plates and lysed in RIPA buffer [50 mM Tris-Cl (PH7.4), 150 mM NaCl, 1 mM EDTA (PH8.0), 0.25% DOC (deoxycholic acid), 10% glycerol, 1% Nonidet P40 and 1% Triton-X100] supplemented with protease inhibitors (Bimake) and phosphatase inhibitors (Bimake). The cell lysates were clarified by centrifugation at 13,000 rpm, for 15 minutes at 4 °C. The protein samples from tumors were homogenized with glass beads and then lysed in RIPA buffer with protease/phosphatase inhibitors. The protein concentrations of the lysates were measured using EASY II Protein Quantitative Kit (Transgene). SDS-PAGE was performed for equal amounts of protein per sample, followed by transferring to a PVDF membrane (Millipore). Then the proteins on the PVDF membrane were immunoblotted with indicated antibodies.

### Immunoprecipitation and pull down

Cells were scraped from dishes and lysed with NETN buffer [20 mM Tris-Cl (PH8.0), 100 mM NaCl, 1 mM EDTA (PH8.0), 0.5% Nonidet P40] containing protease and phosphatase inhibitors. The cell lysates were clarified by centrifugation at 13000 rpm. for 15 minutes at 4 °C.

For immunoprecipitation, the cell lysate was pre-cleared with normal IgG, which had been bound to Protein A/G Agarose (Thermo) or Protein A/G magnetic Beads (Bimake) for 1 hour at room temperature. The pre-cleared cell lysate was then incubated with indicated antibodies which had been bound to Protein A/G Agarose or Protein A/G magnetic Beads for 1 hour at room temperature. After incubating with the cell lysate, the IgG/antibody-bound Protein A/G agarose or magnetic beads were washed 4 times with NETN buffer to remove non-specific binding proteins. Then 1×SDS-PAGE Loading buffer was added to the Protein A/G agarose or magnetic beads. Subsequent immunoblotting was performed as described above.

For S-protein pull down, cell lysates were incubated with S-protein agarose (Millipore) for 4 hours at 4 °C. Then the agarose was washed 4 times with NETN, followed by adding 1×SDS-PAGE Loading buffer and being tested by immunoblotting.

For GST pull down, purified GST-KEAP1 and PD-L1-Flag protein were incubated with GST-tag Purification Resin (Beyotime) for 4 hours at 4 °C, and then wash 3 times with PBS, followed by adding 1×SDS-PAGE Loading buffer and being tested by immunoblotting.

For immunoprecipitation with mouse tumor tissues, fresh xenograft tissues were lysed in NETN buffer like mentioned before, and mixed with PD-L1 antibody for 4 hours at 4 °C; protein A/G agarose beads were added and incubated at 4 °C overnight. Beads were washed three times with NETN buffer and subjected to immunoblotting.

### Immunofluorescence

H1299 and H23 cells were seeded in confocal dishes (Biofilm) for 48 hours and then fixed with 4% paraformaldehyde diluted in PBS for 15 minutes at room temperature. After being washed with PBS for three times, cells were incubated with a blocking buffer containing 0.25% triton X-100 for 1 hour, then incubated with anti-PD-L1 antibody (1:100, Proteintech, 17952-1-AP) and anti-KEAP1 antibody (1:100, Santa Cruz, sc-365626) at 4 °C overnight. And then, cells were washed with PBS for three times and incubated with Alexa Fluor 488-conjugated anti-mouse secondary antibody and Alexa Fluor 555-conjugated anti-rabbit secondary antibody for 2 hours at room temperature followed by incubation with DAPI for 5 minutes before being investigated with confocal microscope.

### Cell proliferation and colony formation assay

Cell proliferation was performed by cell counting kit-8 (CCK-8) assay according to the manufacturer’s requirement. Briefly, indicated cells (1000 − 2000 per well) were seeded in a 96-well plate. Cells were incubated with a 100 μL relative culture medium containing 10% CCK-8 assay solution at different time points. After 2 hours of incubation, OD values were measured at 450 nm using a plate reader (Cytation5, Biotech). Relative growth is calculated to calculate the average OD450 of day1, and then divide each OD values of each day by the average OD value of the first day to obtain relative growth.

For the soft agar colony formation assay, cells (5000 per well) were suspended in a medium containing 0.4% agar and overlaid on 0.7% agar in 6-well plates. Extra liquid medium was added on the surface of ager to keep the top layer moist. The plates were incubated for 3 weeks in a 37 °C incubator, followed by nitrotetrazolium blue chloride staining for 12 hours. Then pictures of the cell colony were taken and the clone numbers were counted with Image J.

### Tumor xenograft experiments

For xenograft model with H1299 cells, 1 × 10^6^ cells were suspended in 100 μl PBS and Matrigel (1:1 v/v), and subcutaneously injected into BALB/c-nu mice (3-5 weeks old). Tumor size was measured every 3 days with a caliper and the tumor volume was determined using the formula: 1/2 × length × width^2^. The mice were sacrificed at the end of the studies and the tumors were dissected and weighted, followed by keeping in liquid nitrogen for protein extraction.

For xenograft model with H1299 cells and combination therapy with MK2206, 1 × 10^6^ H1299 cells suspended in 100 μL PBS and Matrigel (1:1 v/v) and subcutaneously injected into BALB/c-nu mice (3-5 weeks old). A week after injection, tumor size was measured and calculated by using the formula: 1/2 × length × width^2^. MK2206 (120 mg/kg) was administered twice a week from 7 days after inoculation and continued for two weeks.

For xenograft model with LLC cells and combination therapy with anti-PD-L1, 1 × 10^6^ LLC cells were suspended in 100 μL PBS and Matrigel (1:1 v/v) and subcutaneously injected into C57BL/6 mice (3-5 weeks old). Tumor size was measured and calculated by using the formula: 1/2 × length × width^2^. IgG and anti-PD-L1 (10 mg/kg) were intravenously administered twice a week from 14 days after inoculation with respective control treatment and continued for two weeks.

### Tumor sample preparation and flow cytometry

Excised tumors were digested in collagenase/hyaluronidase and DNase at 37 °C for 45 minutes followed by being filtered with a 45 μm filter (BD Bioscience) to obtain cell suspension. Then, cells were stained with PE-conjugated-CD3 antibodies, FITC-conjugated-CD8 antibodies, and APC-conjugated-Granzyme B antibodies. Data acquisition was performed using flow cytometry (Agilent NovoCyte Quanteon) and was analyzed using FlowJo software.

### qRT-PCR analysis for tumor cytokines

Fresh tumor tissues were lysed and total RNA was isolated with RNAiso Plus reagent (TaKaRa). The concentrations of RNA were determined with NanoDrop (Thermo). Reverse transcription PCR was performed using PrimeScript^TM^ RT Master Mix (TaKaRa) to obtain cDNA as template for qRT-PCR. qRT-PCR was performed using StepOnePlus and the DNA double‑strand‑specific reagent SYBR‑Green I for detection (Roche) in the CFX-96 instrument (Bio-Rad). The primer sequences were as follows:

hKEAP1

F 5’-CAACTTCGCTGAGCAGATTGGC-3’

R 5’-TGATGAGGGTCACCAGTTGGCA-3’

hPD-L1

F 5’-TGCCGACTACAAGCGAATTACTG-3’

R 5’-CTGCTTGTCCAGATGACTTCGG-3’

mTNFα

F 5’-CCCTCACACTCAGATCATCTTCT-3’

R 5’-GCTACGACGTGGGCTACAG-3’

mIFNγ

F 5’-ACAGCAAGGCGAAAAAGGATG-3’

R 5’-TGGTGGACCACTCGGATGA-3’

mCCL-5

F 5’-GCTGCTTTGCCTACCTCTCC-3’

R 5’-TCGAGTGACAAACACGACTGC-3’

mCXCL-10

F 5’-TGAATCCGGAATCTAAGACCATCAA-3’

R 5’-AGGACTAGCCATCCACTGGGTAAAG-3’

### Clinical specimens

Human NSCLC Tissue Microarrays were purchased from SHANGHAI OUTDO BIOTECH Company (HLugA180Su04), and all samples have complete clinical follow-up information.

### Immunohistochemistry

The samples were fixed with 4% paraformaldehyde, and embedded with paraffin. Standard IHC staining procedures were performed according to the instructions of the IHC Kit. Specific KEAP1 antibody was used at a dilution ratio of 1:200 (v/v). EDTA and Citrate solution were used for antigen retrieval depending on antibody instruction. IHC staining density was evaluated and estimated based on the average staining intensity and the percentage of positively stained cells. Combining the proportion of positive cells and the intensity, a total score of protein abundance was determined.

### Statistical analysis

Data representative of two or more independent experiments. Statistical analysis was performed using Two-way ANOVA test and Student’s *t*-test (unpaired, two-tailed) to compare two groups of independent samples, and a difference with *P* value < 0.05 was considered statistically significant. Bars and error represent mean ± standard deviations (SD) or standard error of the mean (SEM) of replicate measurements. *P < 0.05, **P < 0.01, ***P < 0.001,****P < 0.0001. All of the relative protein expression was normalized via comparing to the corresponding grey values of GAPDH or vinculin achieved by ImageJ. The survival curve was achieved using the Kaplan-Meier estimator. The correlation analysis between KEAP1 and PD-L1 was performed with Pearson Correlation Coefficient and Chi-square test. The detailed methods for TCGA data analysis and pathway enrichment were presented in the Computational analysis part below.

### Computational analysis

#### Somatic mutation analysis

Two independent sets of data were analyzed. First, the mutation data (.maf format) of somatic mutation genes (SMGs) were downloaded from the TCGA pan-cancer atlas (https://gdc.cancer.gov/about-data/publications/pancanatlas). Secondly, a panel of 425 genes was sequenced for the collected NSCLC patients, and the mutations were identified. The sequencing and analysis were done by Geneseeq Technology Inc.

#### Pathway enrichment analysis

The RNA-seq data of TCGA-LUAD cohorts were downloaded from cBioProtal (https://www.cbioportal.org/). We calculated the Pearson correlation coefficients between KEAP1 and all other genes according to their mRNA expression levels in the RNA-seq datasets. Furthermore, to obtain a genome-wide perspective of KEAP1 impacts on pathways, we also ranked all the genes in the RNA-seq data based on the Pearson correlation coefficients and utilized the ranked genome-wide gene list as the input of the GSEA software [38], and performed GSEA-based pathway enrichment. Pathway genes were obtained from the Kyoto Encyclopedia of Genes and Genomes (KEGG) [39], (https://www.kegg.jp/).

#### Survival analysis

For a single protein, patients were separated into two groups with expressions higher or lower than the median expression of the gene, then we utilized the log-rank test to compare the overall survival time between groups, and survival curves were created based on Kaplan-Meier estimator. To determine whether the inverse correlations between KEAP1 and PD-L1 can cooperatively impact the prognosis, we utilized the log-rank test to compare the overall survival in all four groups, and survival curves were created based on the Kaplan-Meier estimator.

### Reporting summary

Further information on research design is available in the Nature Research Reporting Summary linked to this article.

## Supplementary information

Supplementary materials

Original Data File

Reporting Summary

## Data Availability

All data generated or analyzed in this study are included in this published article and its supplemental information files. All the data that support the findings of this study are available from the corresponding authors.
