# Correction: KEAP1 promotes anti-tumor immunity by inhibiting PD-L1 expression in NSCLC

**DOI:** 10.1038/s41419-024-07292-3

**Published:** 2025-01-07

**Authors:** Jinghan Li, Daiwang Shi, Siyi Li, Xiang Shi, Yu Liu, Yi Zhang, Gebang Wang, Chenlei Zhang, Tian Xia, Hai-long Piao, Hong-Xu Liu

**Affiliations:** 1 Department of Thoracic Surgery, Cancer Hospital of China Medical University, Liaoning Cancer Hospital & Institute, Shenyang, 110042 China; 2 Dalian Institute of Chemical Physics, Chinese Academy of Sciences, Dalian, 116023 China; 3 Department of Thoracic Surgery, Shengjing Hospital, China Medical University, Shenyang, Liaoning China; 4 Department of Biochemistry & Molecular Biology, School of Life Sciences, China Medical University, Shenyang, 110122 China

**Keywords:** Non-small-cell lung cancer, Cancer immunotherapy, Ubiquitylation

Correction to: *Cell Death and Disease* 10.1038/s41419-024-06563-3, publish!ng19ed online 27 February 2024

We are willing to recognize and correct our mistakes and provide the correct results to our readers. And we want to emphasize that these errors occurred during the image assembly stage, and we take full responsibility for them. We have now replaced the images with the corrected versions, and we have thoroughly cross-checked the original data to ensure the accuracy. (Figs. 5F, 6H and 6I).

**Fig 5f amended** 
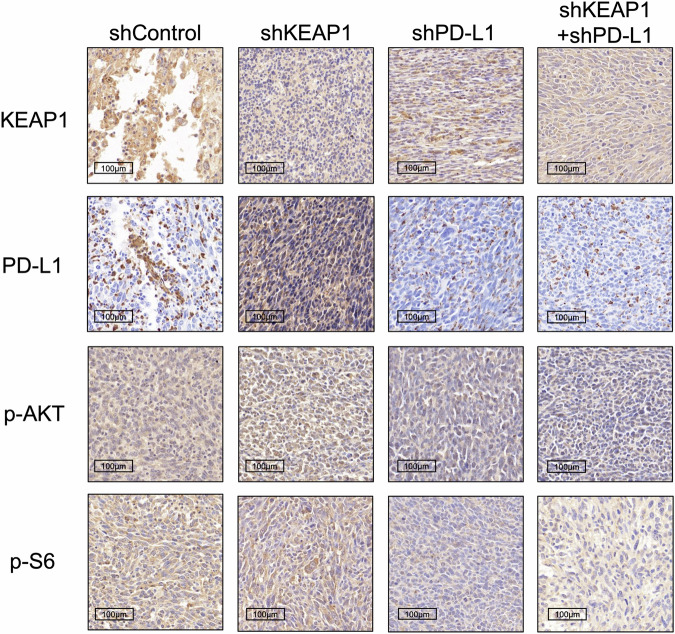


**Fig 5f original** 
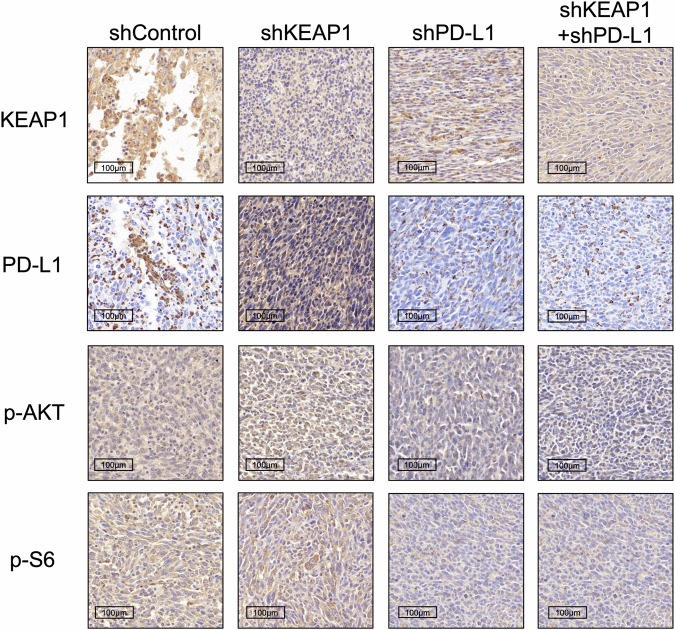


**Fig 6h amedned** 
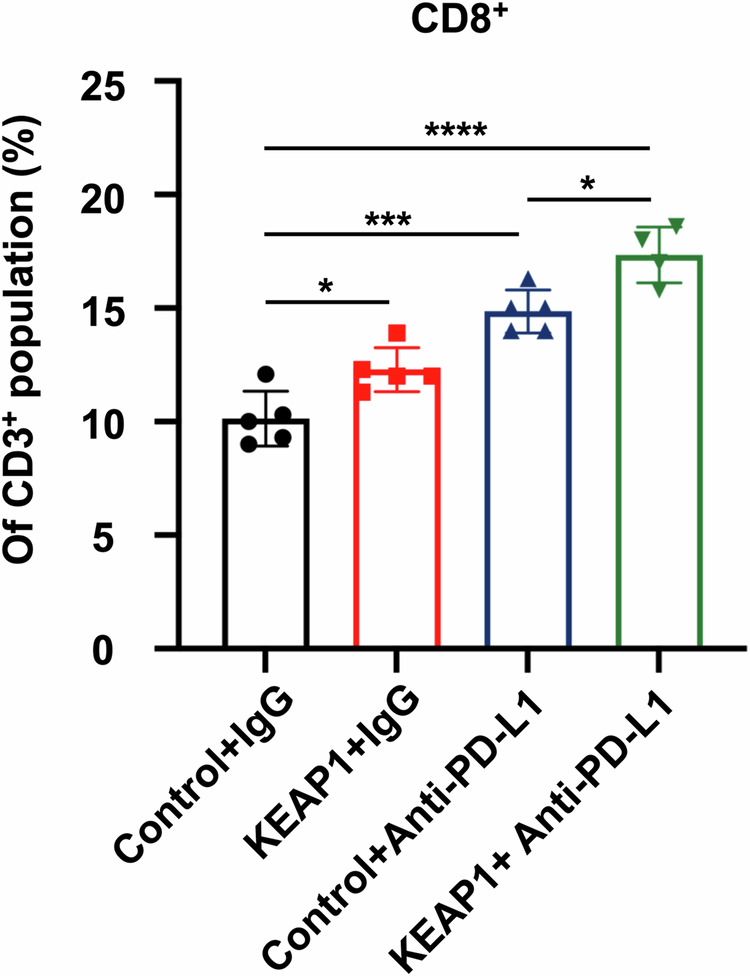


**Fig 6h original** 
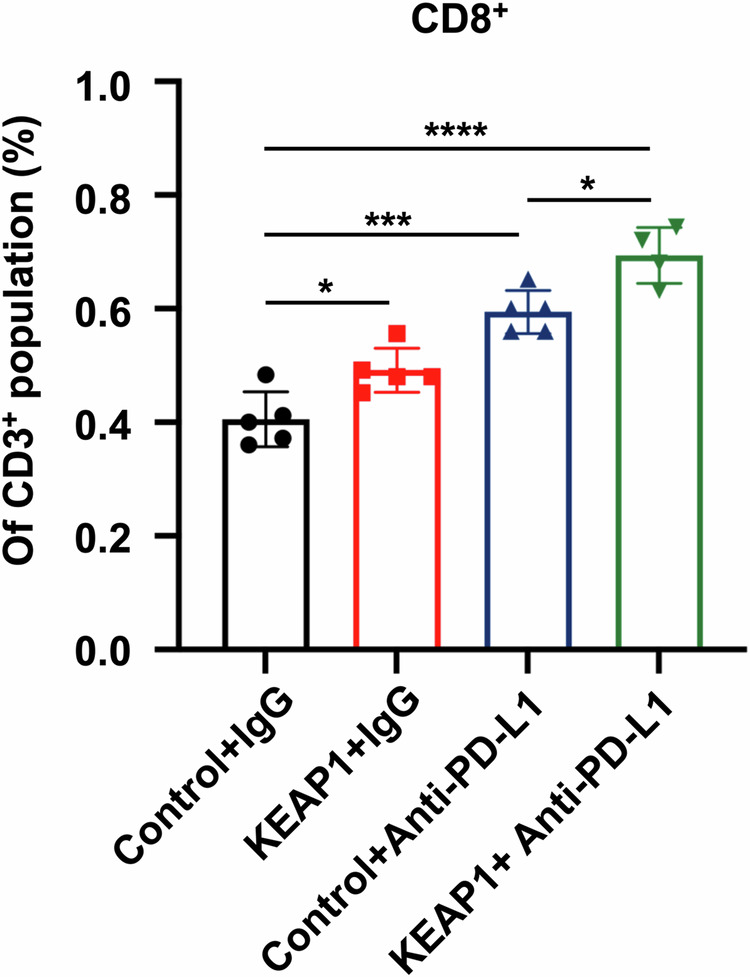


**Fig 6I amedned** 
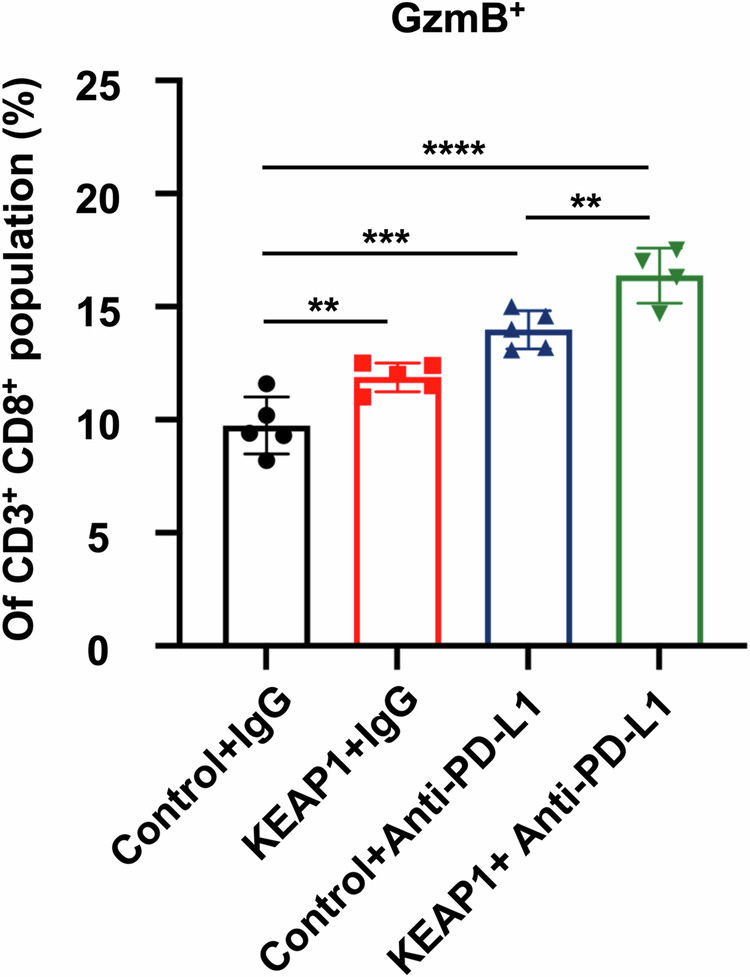


**Fig 6I original** 
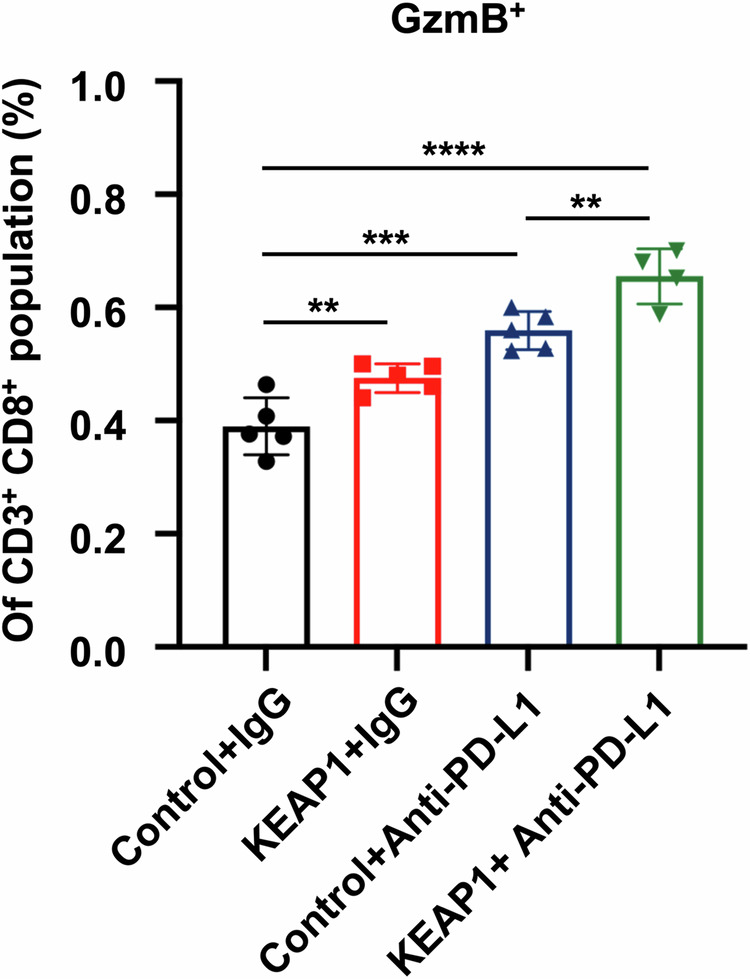


The original article has been corrected.

