# Supplementary material for: KEAP1 promotes anti-tumor immunity by inhibiting PD-L1 expression in NSCLC
Source: Cell Death Dis. 2024 Feb 27;15(2):175. doi: 10.1038/s41419-024-06563-3 (PMC10899596; doi:10.1038/s41419-024-06563-3)
Supplement: Supplementary file 1 — Supplementary materials [file 41419_2024_6563_MOESM1_ESM.docx]

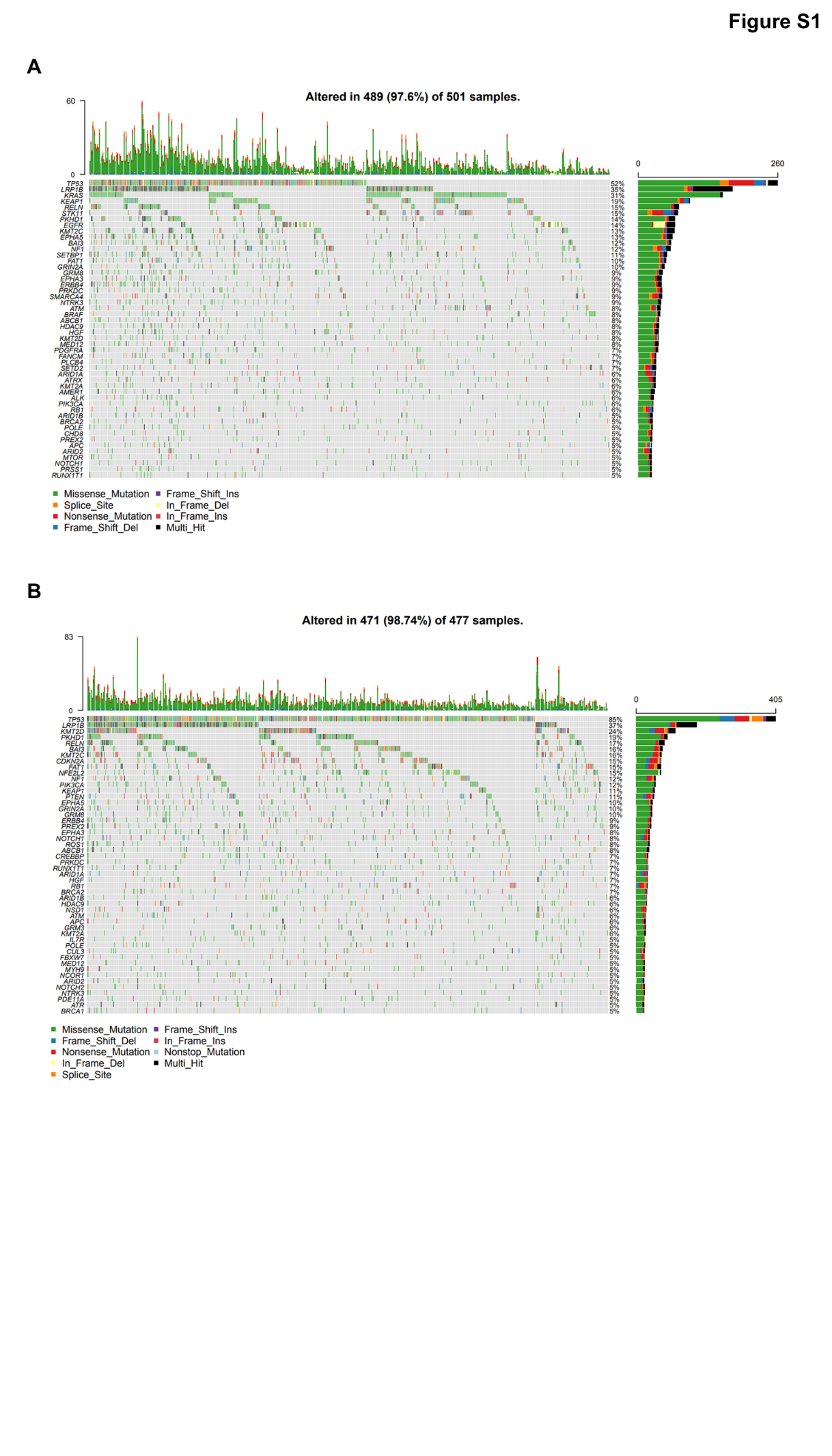


**Supplementary Figure 1 | Genetic mutation analysis of TCGA database**

**(A) and (B)** Somatic gene mutation analysis results based on TCGA-LUAD (A) and TCGA-LUSC (B) database.


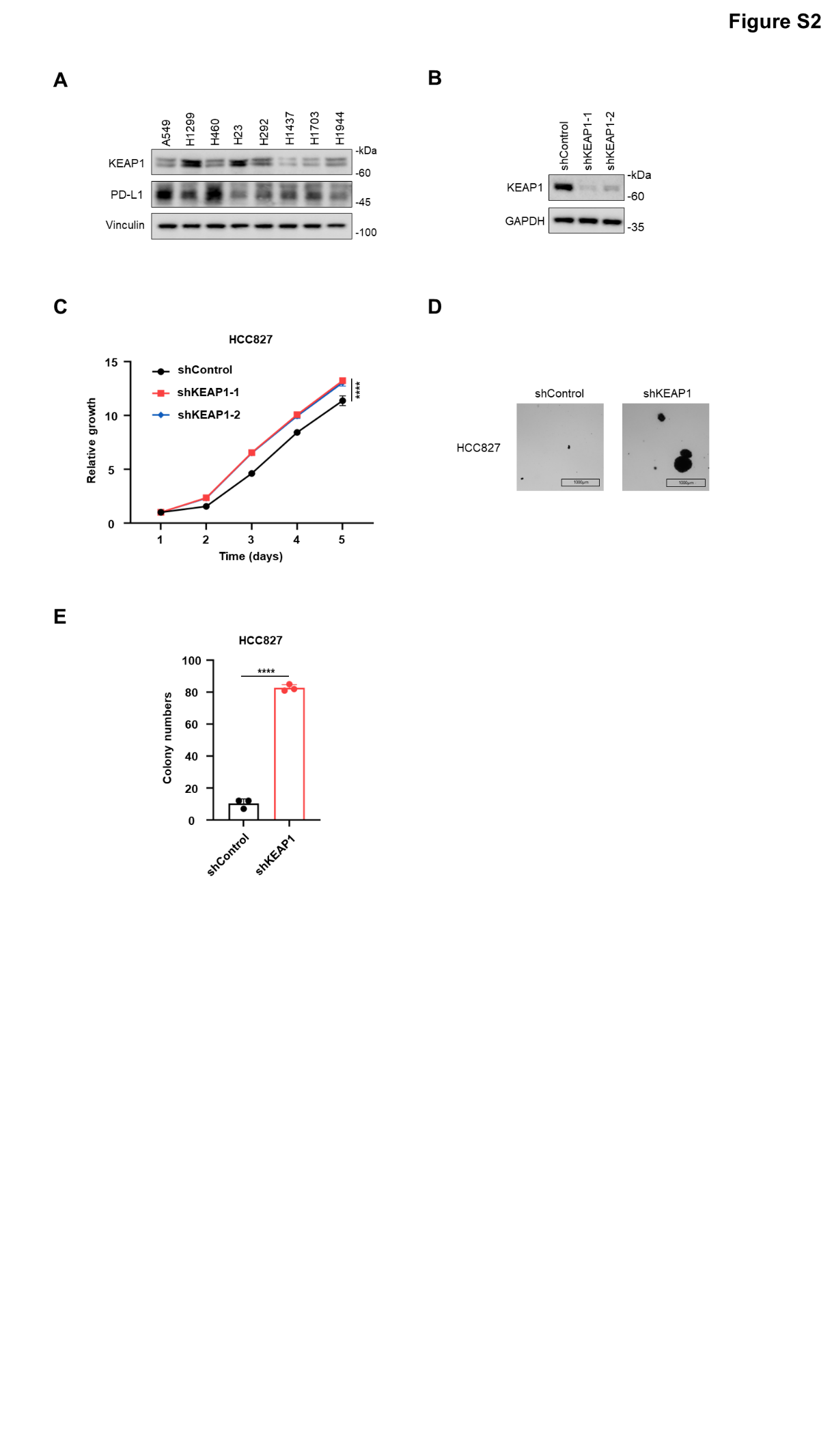


**Supplementary Figure 2 | KEAP1 inhibits NSCLC cell growth and colony formation**

**(A)** Expression analysis of KEAP1 and PD-L1 in NSCLC cell lines by immunoblotting.

**(B)** Expression of KEAP1 in KEAP1 knockdown HCC827 cells was analyzed by immunoblotting.

**(C)** Cell growth curves of KEAP1 knockdown HCC827 cells according to the CCK8 assays.

**(D) and (E)** The colony formation assay of KEAP1 knockdown HCC827 cells in soft agar. The colonies were represented by nitrotetrazolium blue chloride staining followed by capturing pictures (D). Then the colony numbers were calculated by Image J (E). n=3 wells per group. Scale bar: 1000 μm.

Data in D is presented as mean values ± SD. Unpaired t test.


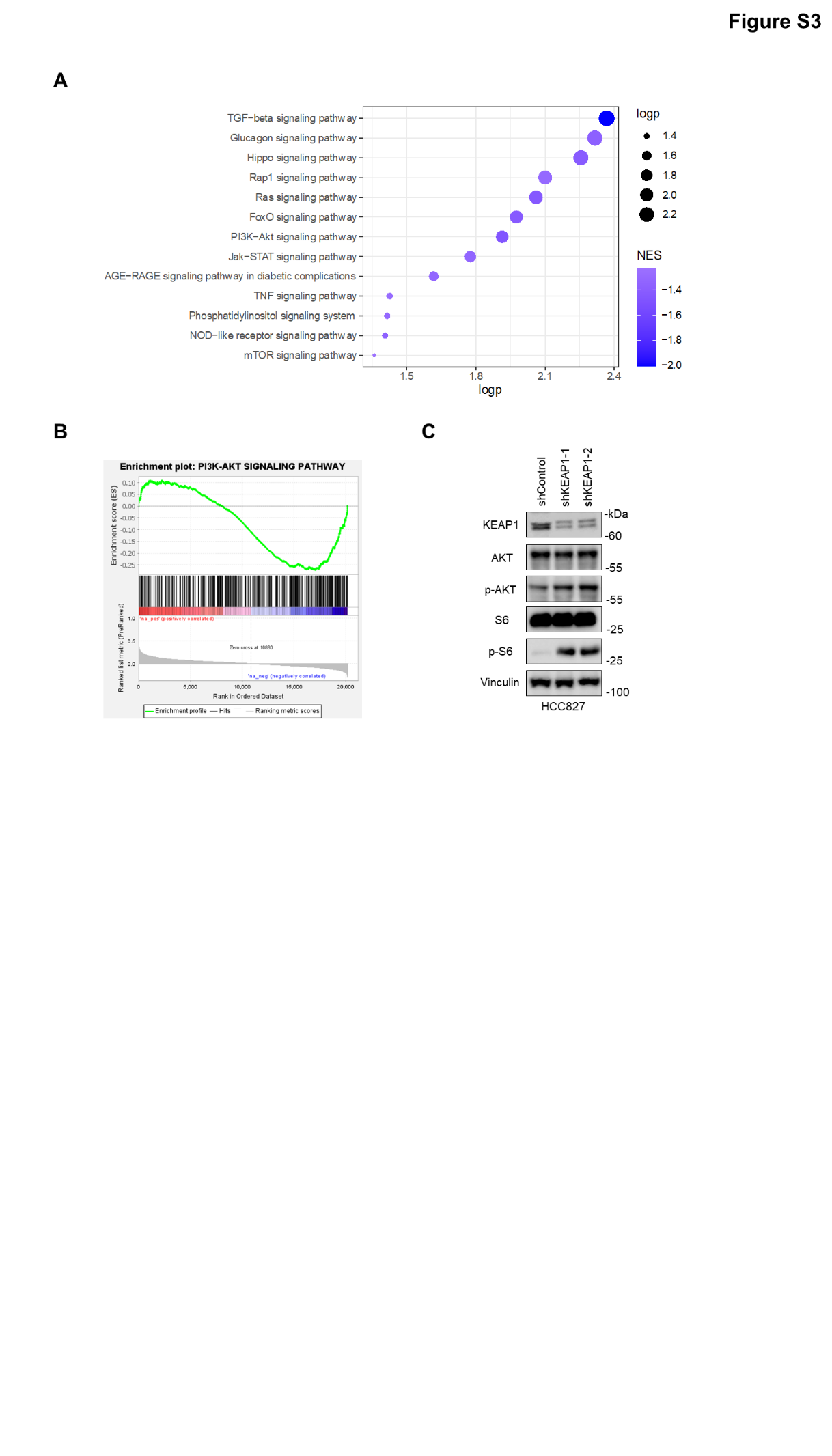


**Supplementary Figure 3 | KEAP1 regulates the PI3K-AKT signaling in NSCLC**

**(A**) Signaling pathway enrichment analysis based on the KEAP1-correlated genes. The correlations between KEAP1 and all the other genes were computed based on the RNA-seq data from the TCGA-LUAD database.

**(B)** GSEA results showing negative correlations between KEAP1 and PI3K-AKT signaling pathway based on the RNA-seq data from the TCGA-LUAD database.

**(C)** Immunoblotting analysis of KEAP1, AKT, p-AKT, S6 and p-S6 in KEAP1 knockdown HCC827 cells.


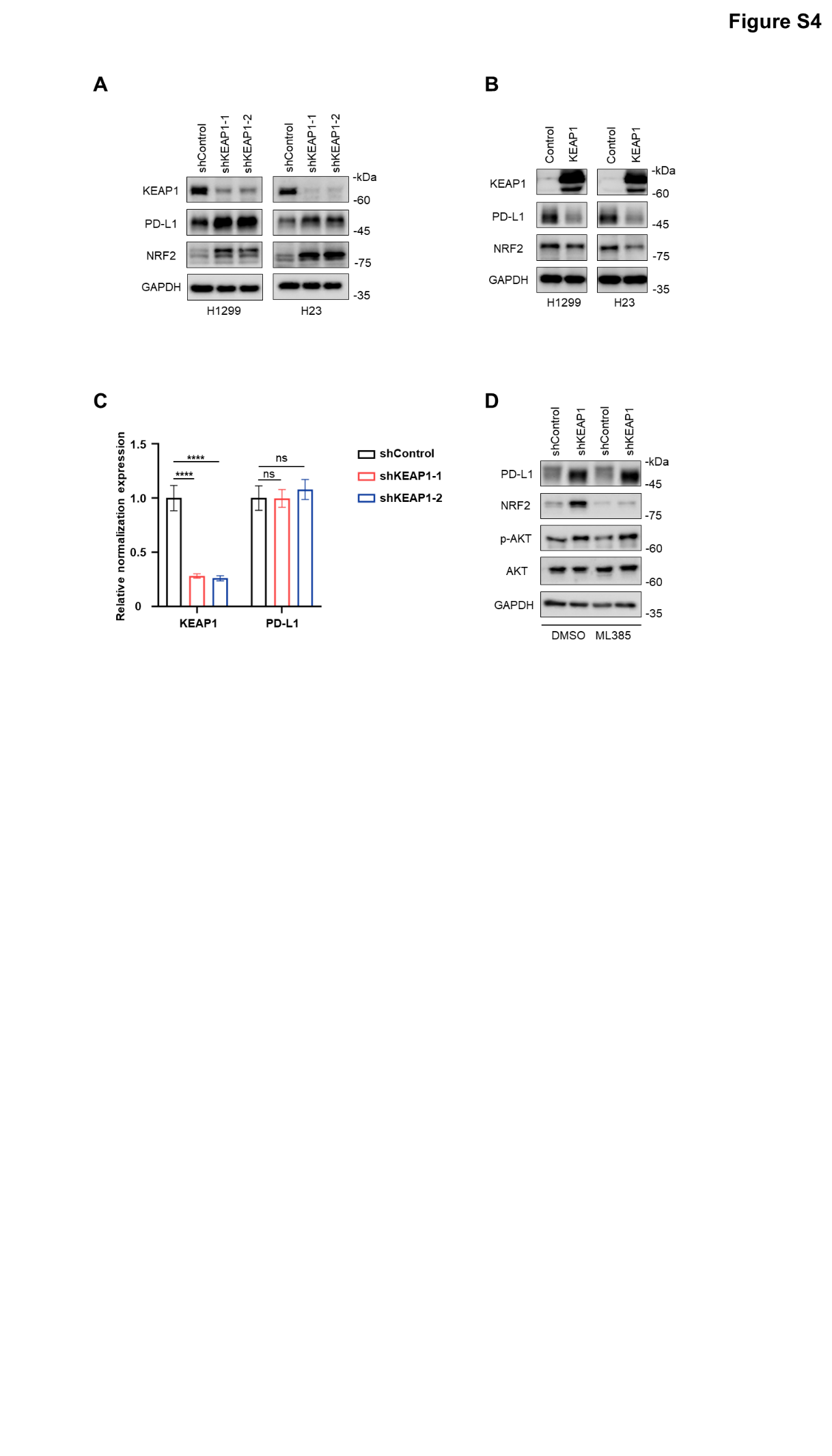


**Supplementary Figure 4 | KEAP1 directly interacts with PD-L1**

**(A) and (B)** Immunoblotting analysis of KEAP1, PD-L1, and NRF2 in KEAP1 knockdown (A) and overexpression (B) H1299 and H23 cells.

**(C)** RNA of shControl, shKEAP1-1 and shKEAP1-2 H1299 cells followed by reverse-transcription to obtain cDNA. The cDNA was used for qRT-PCR analysis to investigate the expression of KEAP1 and PD-L1.

**(D)** The KEAP1 knockdown H1299 cells were treated with 5μM ML385 for 24 hours and then the cells were lysed to test the levels of PD-L1 and NRF2 by immunoblotting.

Data in D is presented as mean values ± SEM, unpaired t test.


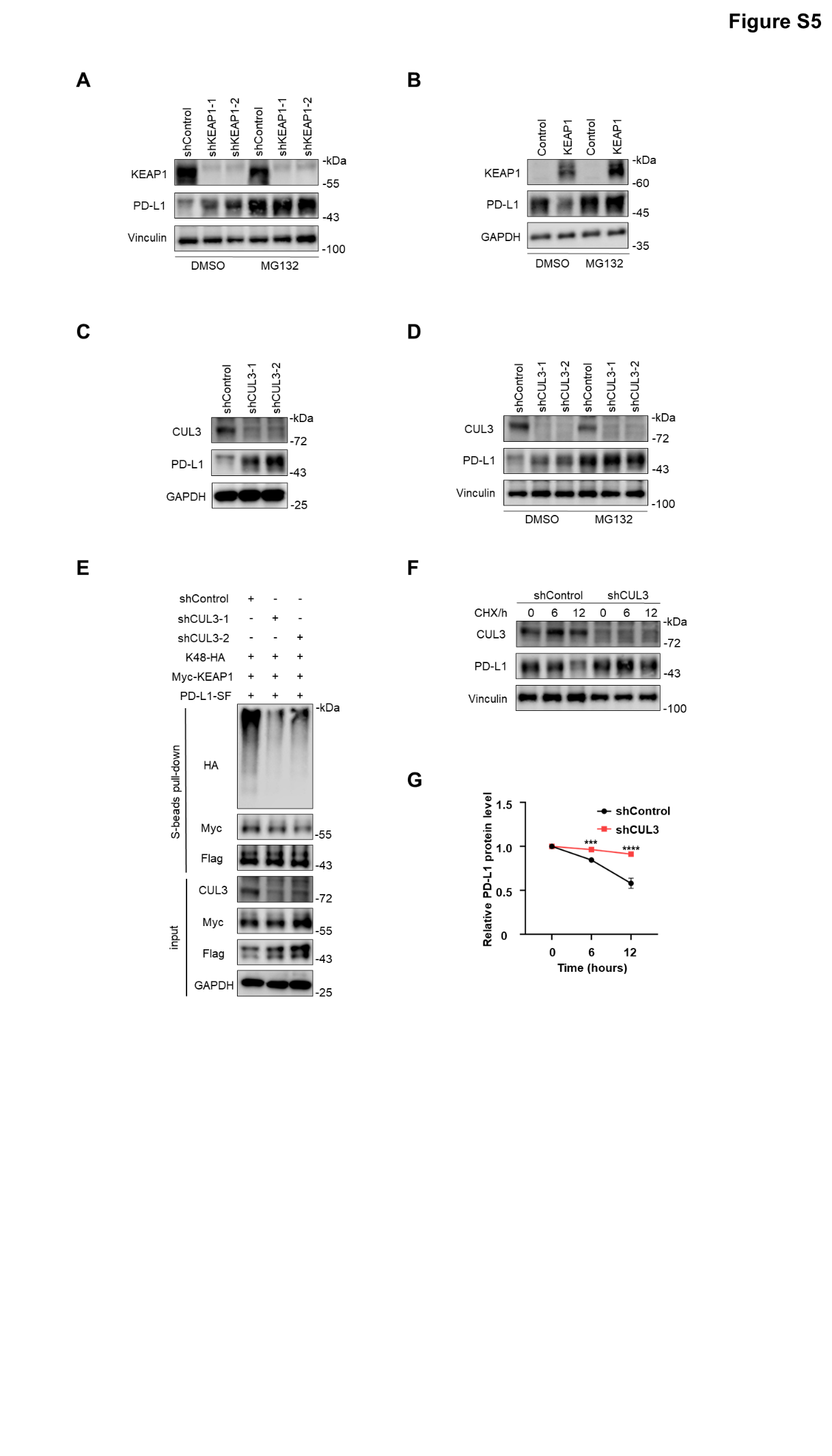


**Supplementary Figure 5 | KEAP1 ubiquitinates PD-L1 through 26S-proteosome route**

**(A) and (B)** The KEAP1 knockdown (A) and overexpression (B) H1299 cells were respectively treated with DMSO and MG132, then the cells were lysed to test the level of KEAP1 and PD-L1.

**(C)** Expression of CUL3 and PD-L1 in CUL3 knockdown H1299 cells was analyzed by immunoblotting.

**(D)** The CUL3 H1299 knockdown cells were respectively treated with DMSO and MG132, then the cells were lysed to test the level of CUL3 and PD-L1.

**(E)** Ubiquitination assay of PD-L1 in shControl, shCUL3-1 and shCUL3-2 HEK293T cells co-transfected with Myc-KEAP1, PD-L1-SF and K48-HA with the treatment of 10 μM MG132 for 6 hours. Then the cells were lysed and immunoprecipitated with S-beads, the indicated protein levels were tested by immunoblotting.

**(F) and (G)** The shControl and shCUL3 H1299 cells were treated with 40 mΜ cycloheximide (CHX) for indicated times. Then immunoblotting was performed to test the protein levels in cell lysate (F) and the protein levels were quantified according to the grey values (G).

Data in G is presented as mean values ± SD, two-way ANOVA test.


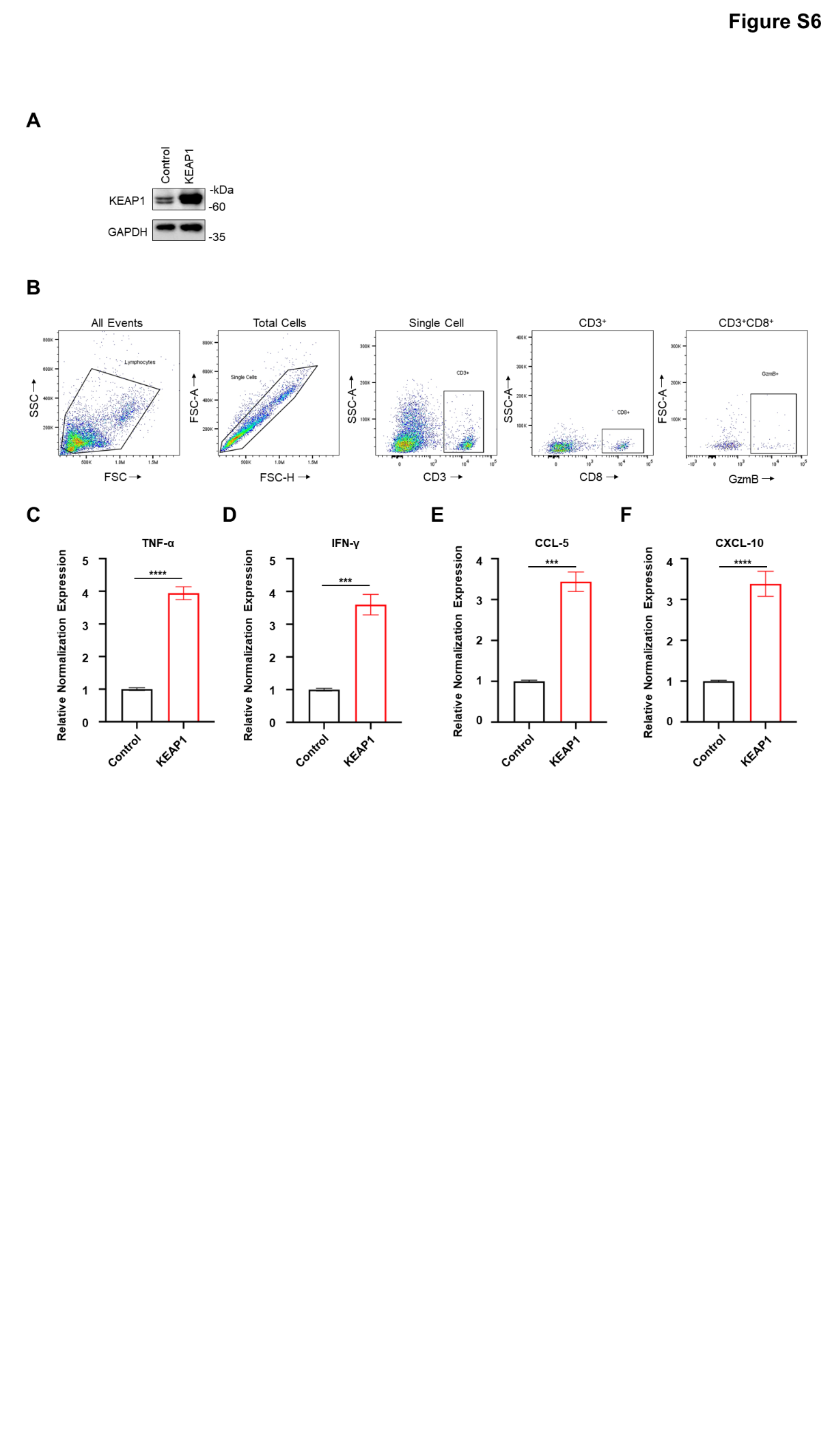


**Supplementary Figure 6 | KEAP1 promotes anti-tumor immunity via PD-L1 degradation**

**(A)** Exogenous KEAP1 was stably expressed in LLC cells, and the KEAP1 expression of this cell line was determined by immunoblotting.

**(B)** Gating procedure for CD8^+^ T cells and GzmB^+^ T cells in tumor microenvironment.

**(C)-(F)** RNA of tumor derived from injected control and KEAP1 overexpression LLC cells were extracted followed by reverse-transcription to obtain cDNA. The cDNA was used for qRT-PCR analysis to investigate the expression of TNF-α (A), IFN-γ (B), CCL-5 (C) and CXCL-10 (D) (n = 4-5).

Data in C-F are presented as mean values ± SEM. Unpaired t test.


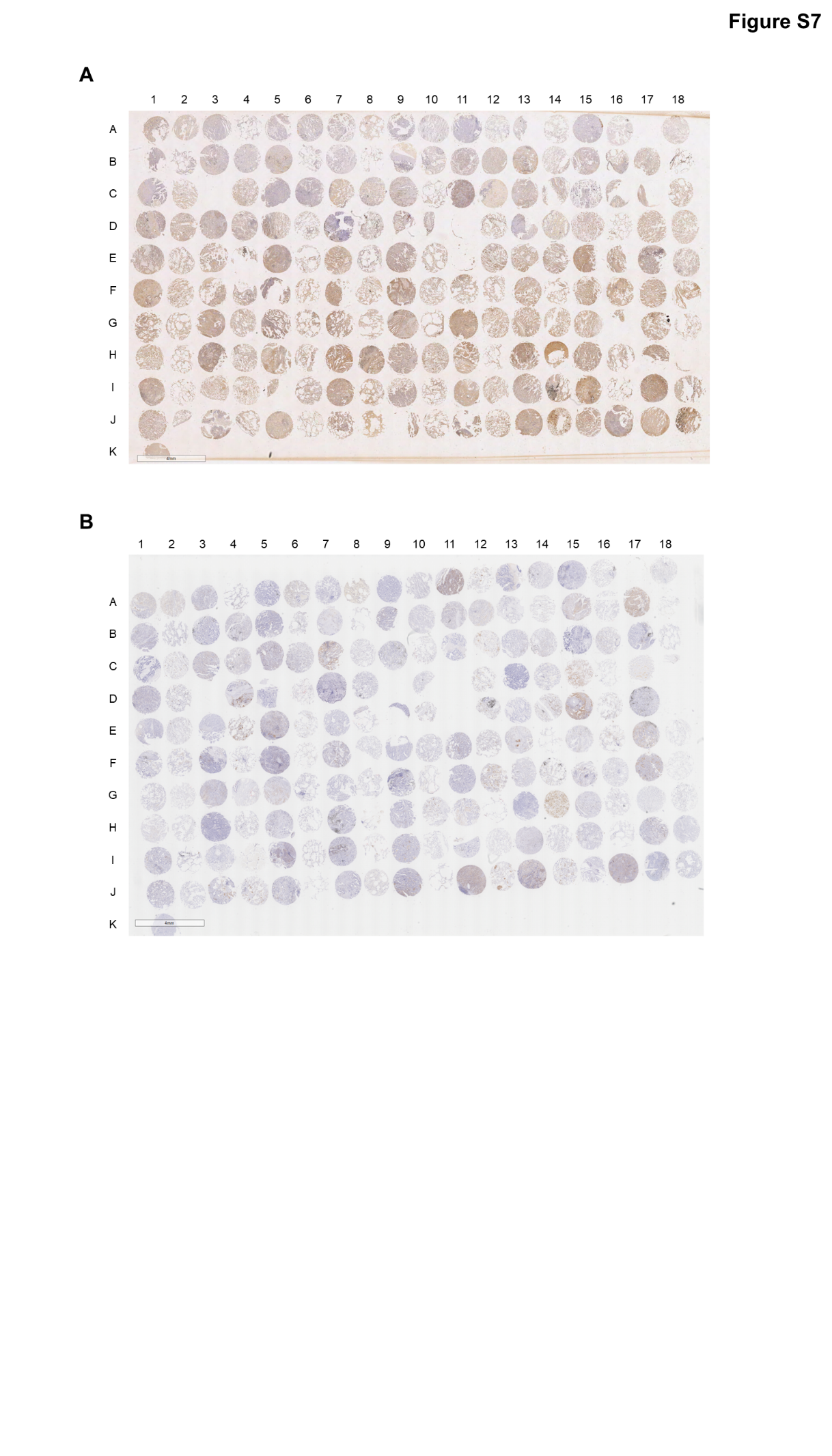


**Supplementary Figure 7 | KEAP1 is negatively correlated with PD-L1**

**(A) and (B)** Immunohistochemical staining shows the KEAP1 (A) and PD-L1 (B) expression in samples on TMAs (HLugA180Su04). Scale bar: 4 mm.


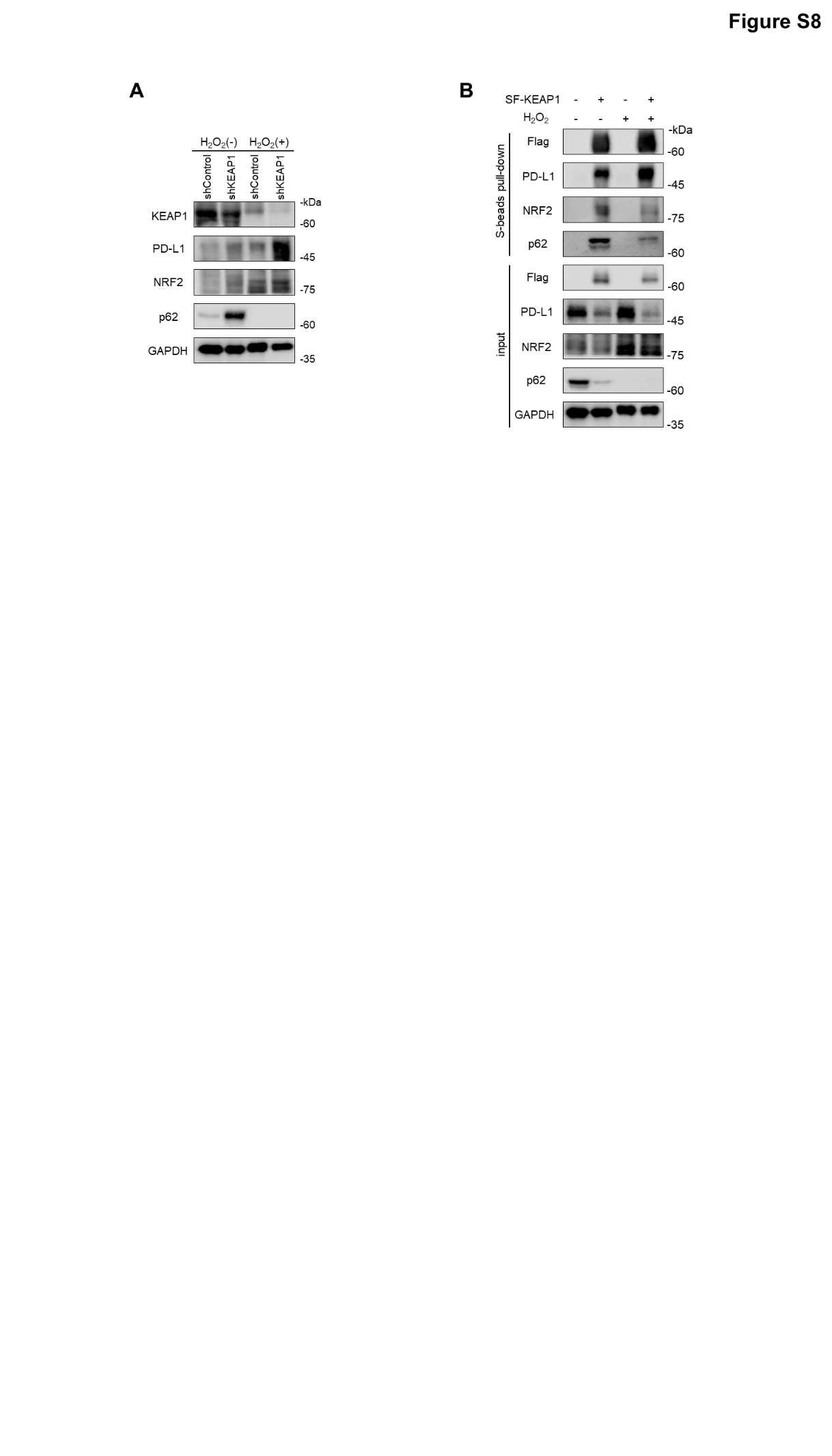


**Supplementary Figure 8 | PD-L1 expression is elevated under oxidative stress.**

**(A)** The shControl and shKEAP1 H1299 cells were treated with or without 20μM H_2_O_2_ for 2 hours, the cells were lysed to test the level of KEAP1, PD-L1, NRF2 and p62 by immunoblotting.

**(B)** HEK293T cells were transfected with SF-KEAP1 and treated with or without 20μM H_2_O_2_ for 2 hours, cell lysates were pulled down with S-beads, and immunoblotting was performed to test the interaction of KEAP1, PD-L1, NRF2 and p62.
