# Supplementary material for: KEAP1 promotes anti-tumor immunity by inhibiting PD-L1 expression in NSCLC
Source: Cell Death Dis. 2024 Feb 27;15(2):175. doi: 10.1038/s41419-024-06563-3 (PMC10899596; doi:10.1038/s41419-024-06563-3)
Supplement: Supplementary file 2 — Original Data File [file 41419_2024_6563_MOESM2_ESM.pdf]

Figure 1A

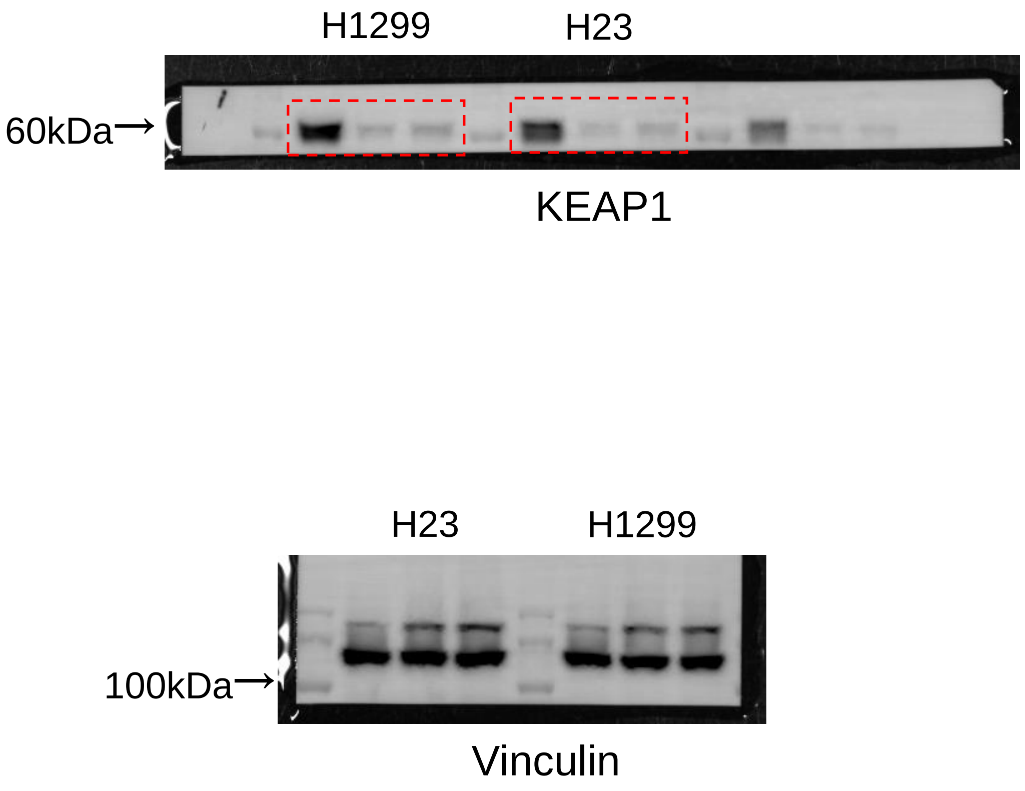

Figure 1B

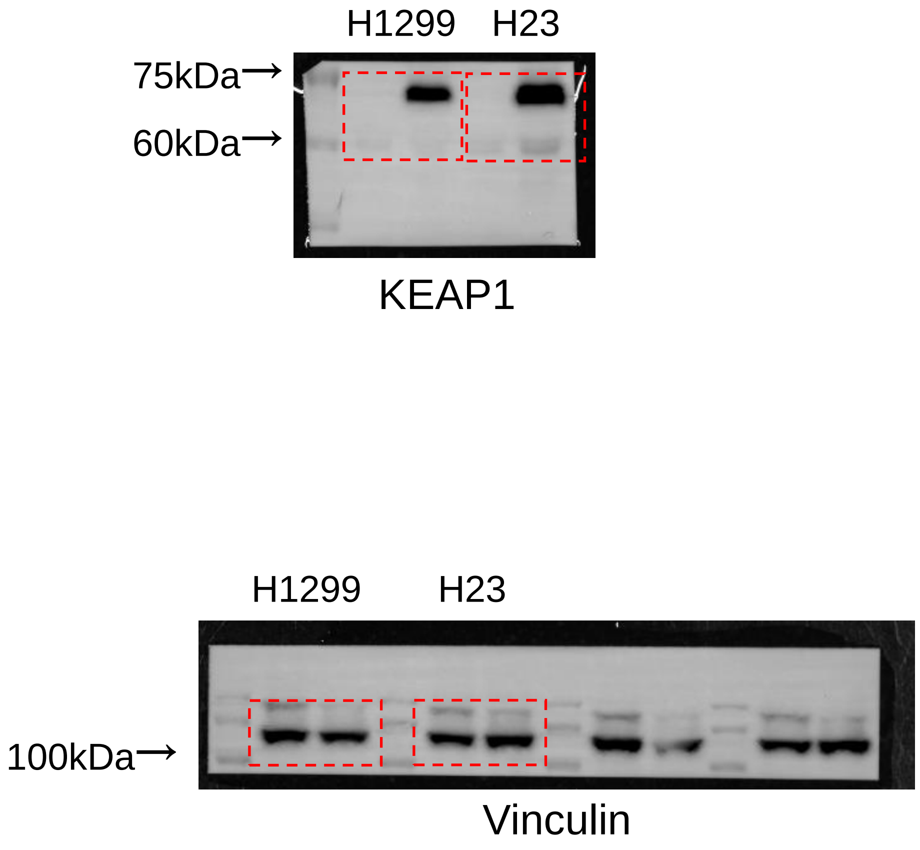

Figure 2A

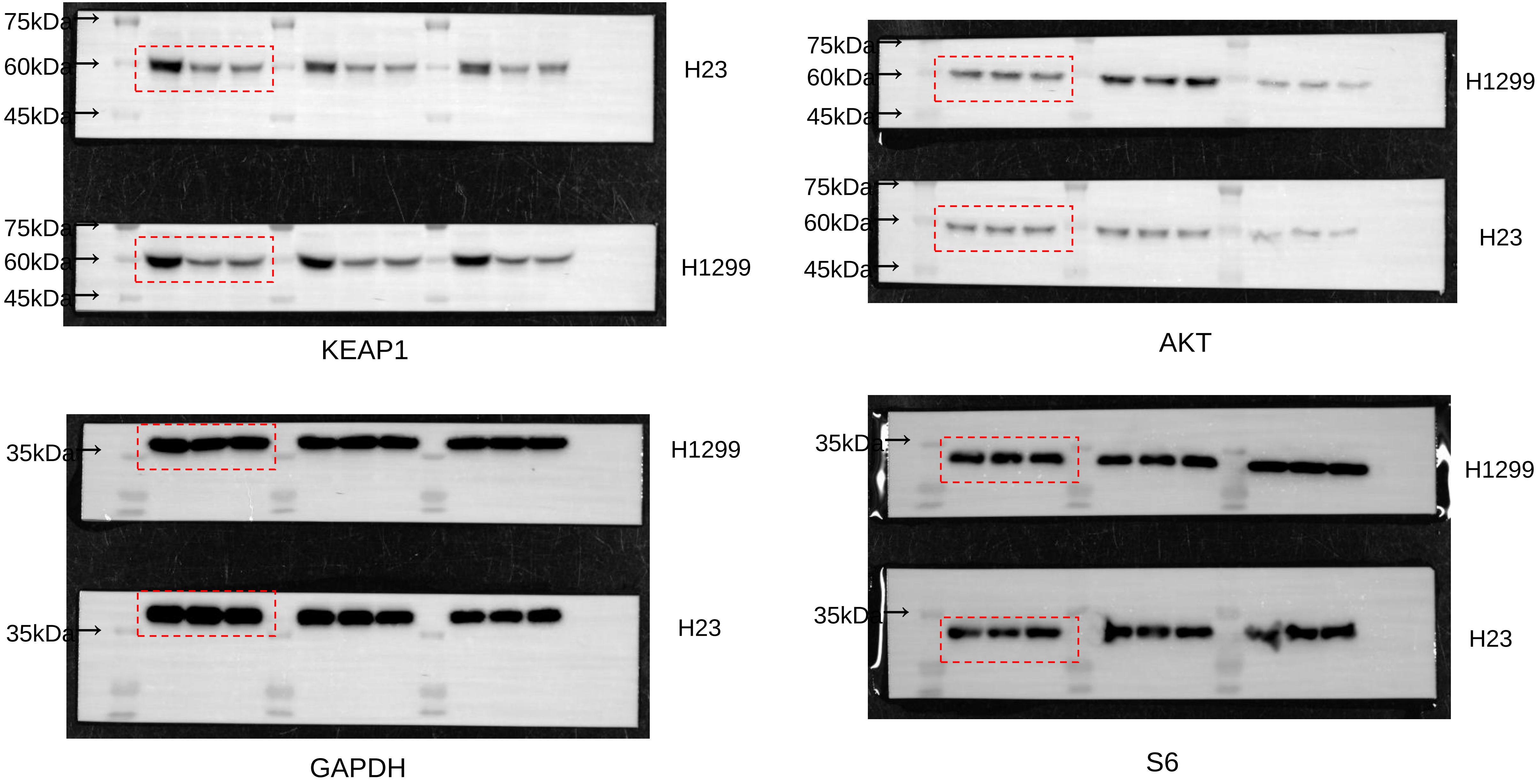
75kDa
60kDa
45kDa
H23
H1299
KEAP1
H1299
H23
AKT
35kDa
H1299
H23
GAPDH
S6

Figure 2A

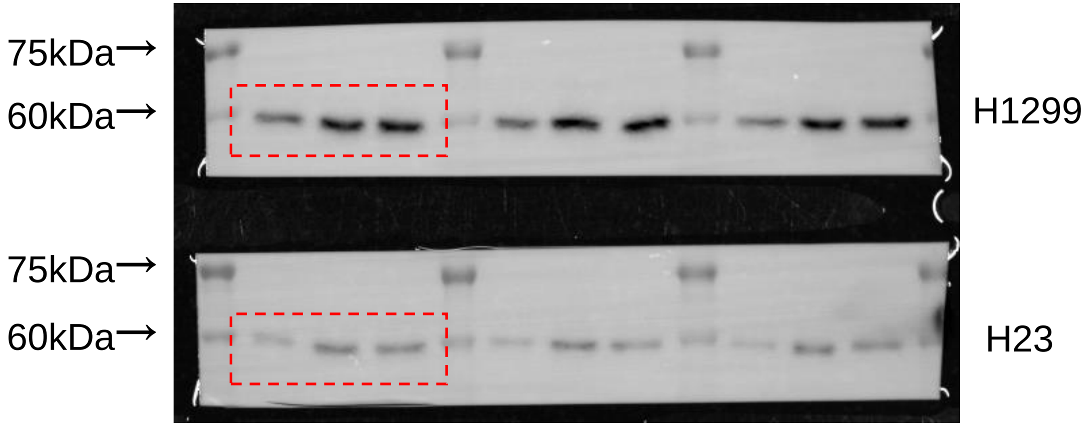

p-AKT

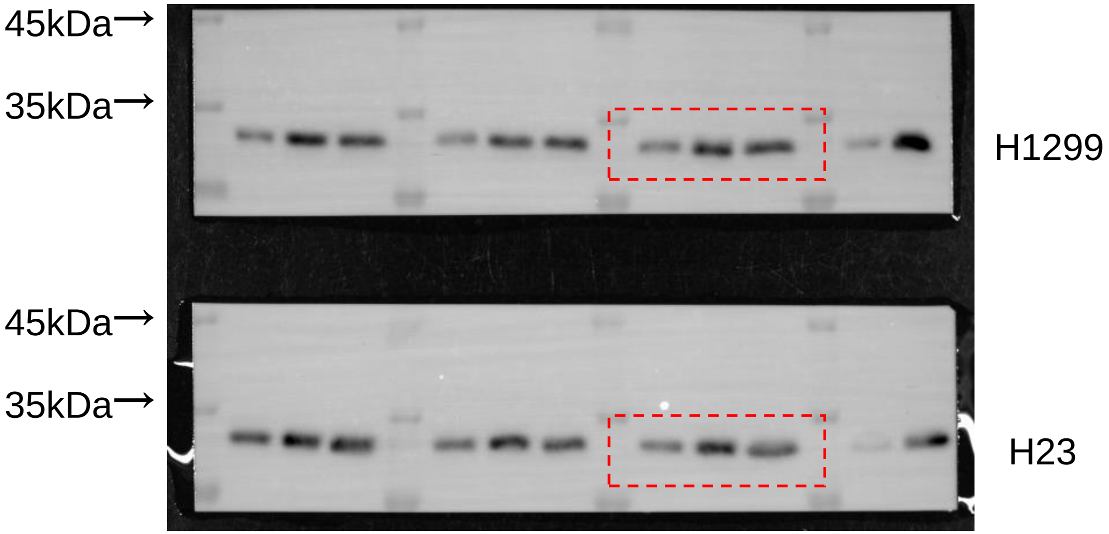

p-S6

Figure 2B

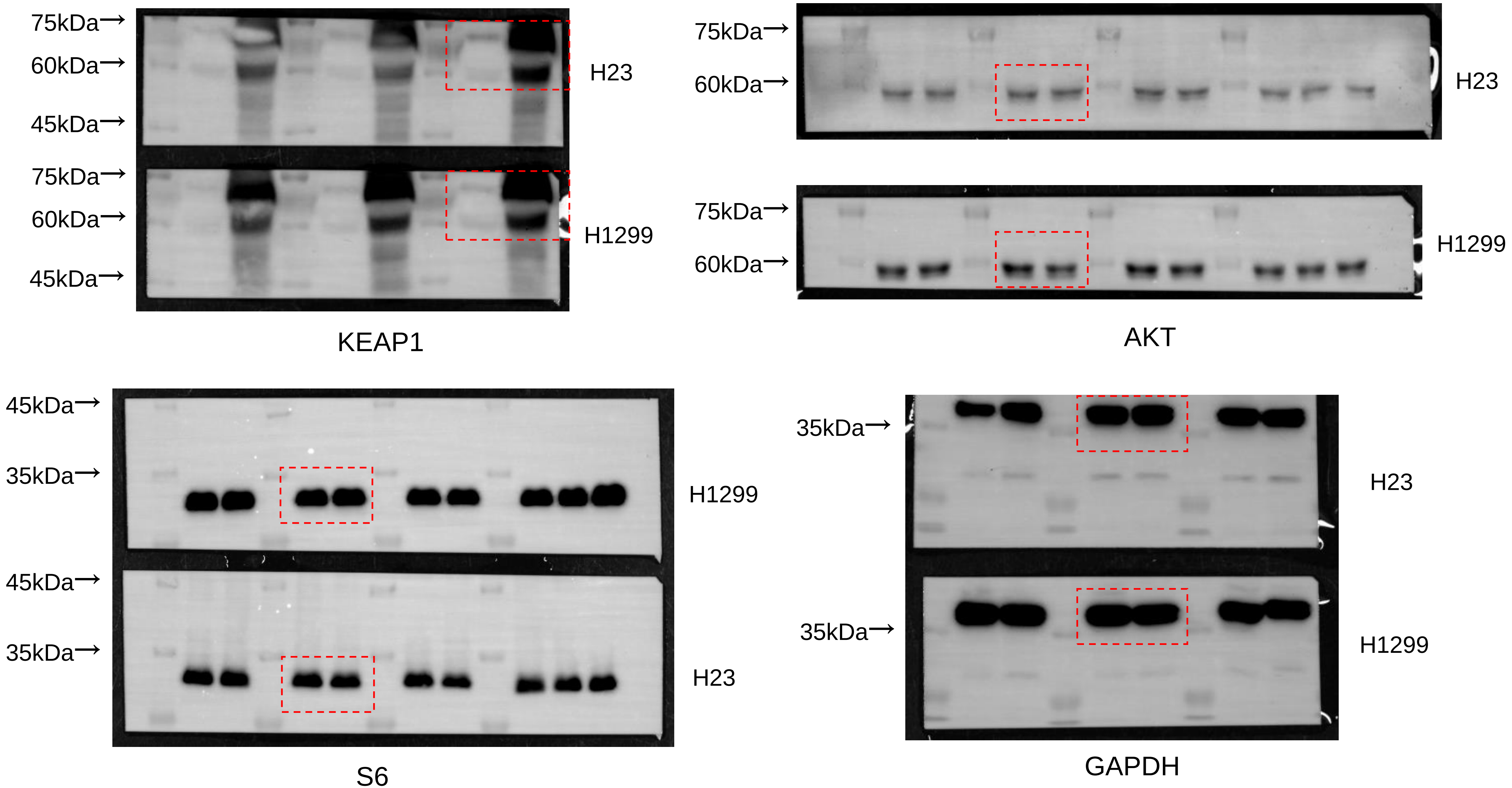
75kDa
60kDa
45kDa
H23
75kDa
60kDa
45kDa
H1299
KEAP1
75kDa
60kDa
H23
75kDa
60kDa
H1299
AKT
45kDa
35kDa
H1299
45kDa
35kDa
H23
S6
35kDa
H23
35kDa
H1299
GAPDH

Figure 2B

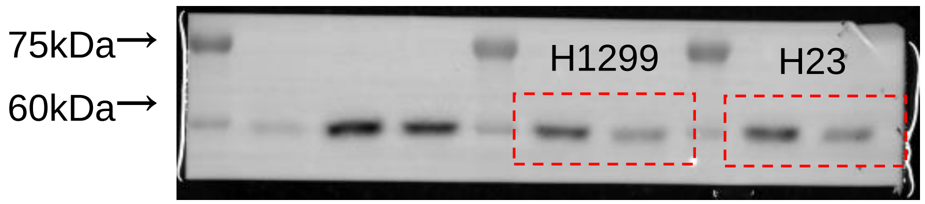

p-AKT

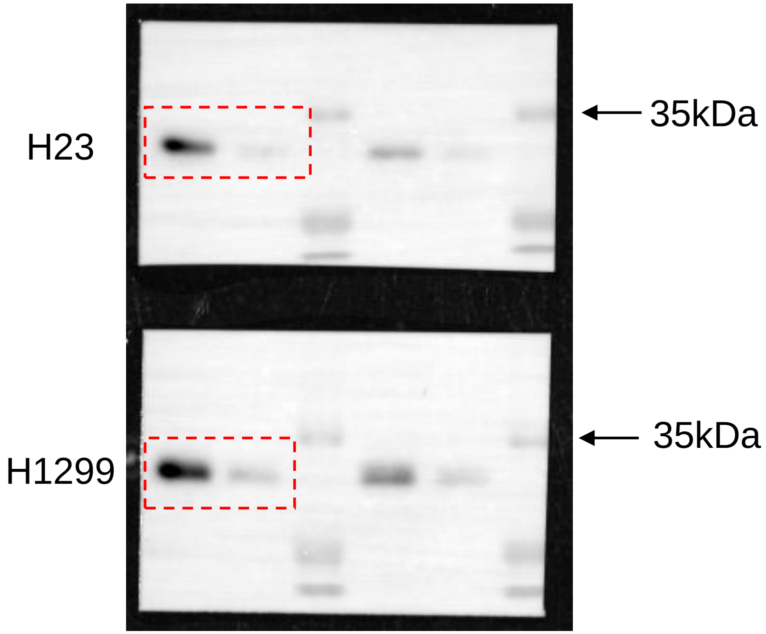

p-S6

Figure 2C

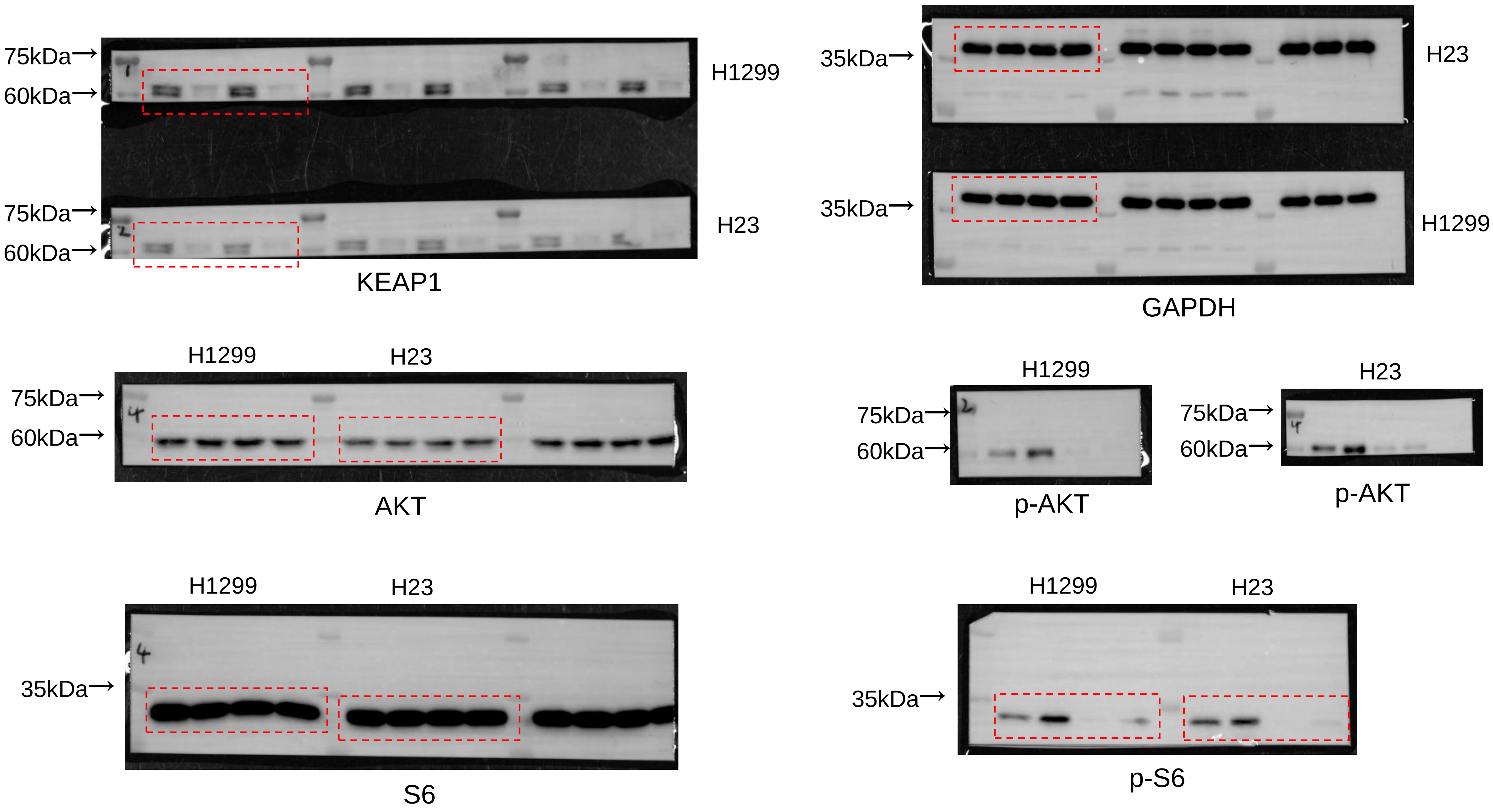
75kDa
60kDa
H1299
75kDa
60kDa
H23
KEAP1
35kDa
H23
35kDa
H1299
GAPDH
H1299
H23
75kDa
60kDa
AKT
H1299
75kDa
60kDa
p-AKT
H23
75kDa
60kDa
p-AKT
H1299
H23
35kDa
S6
H1299
H23
35kDa
p-S6

Figure 3B

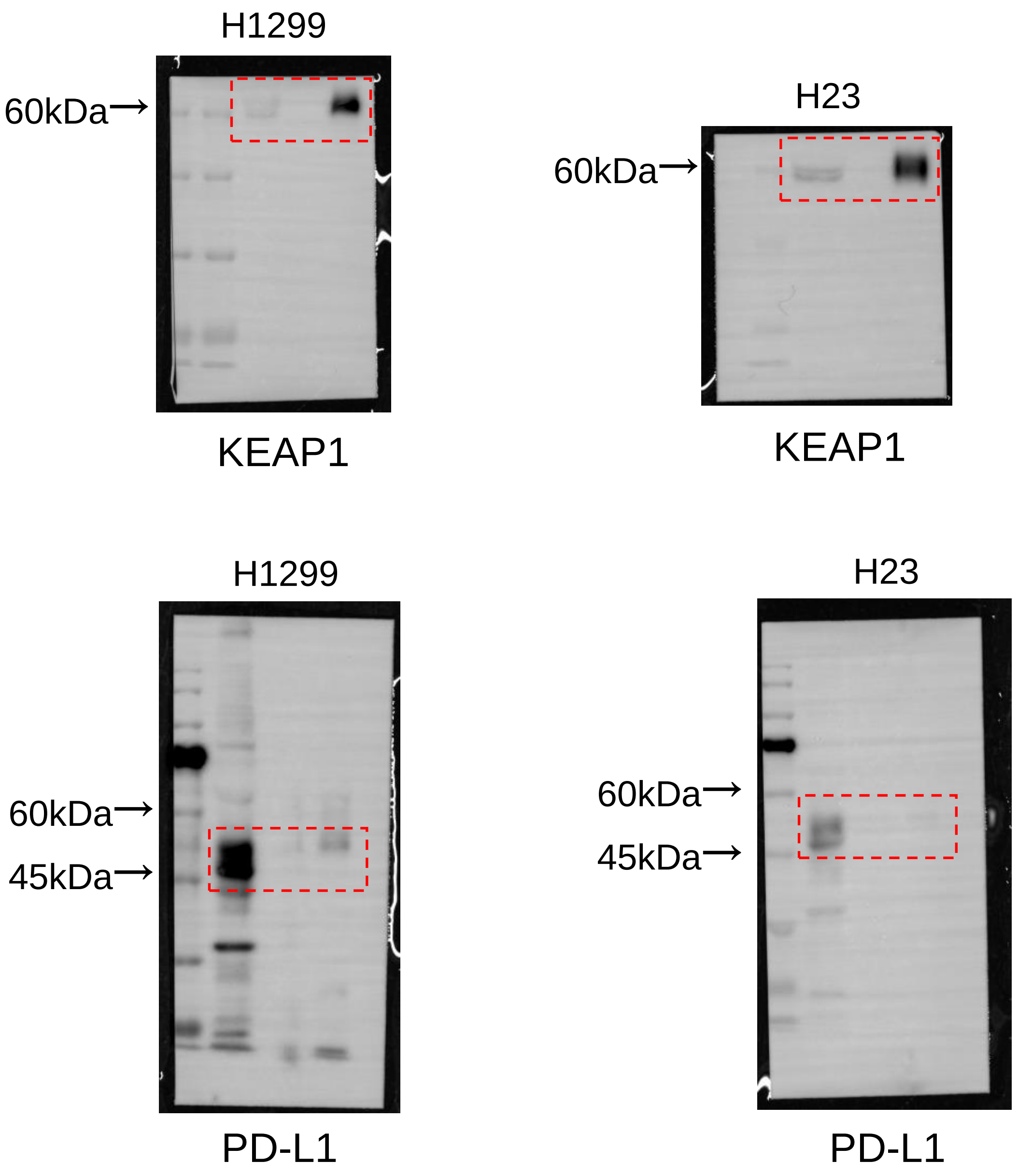

Figure 3C

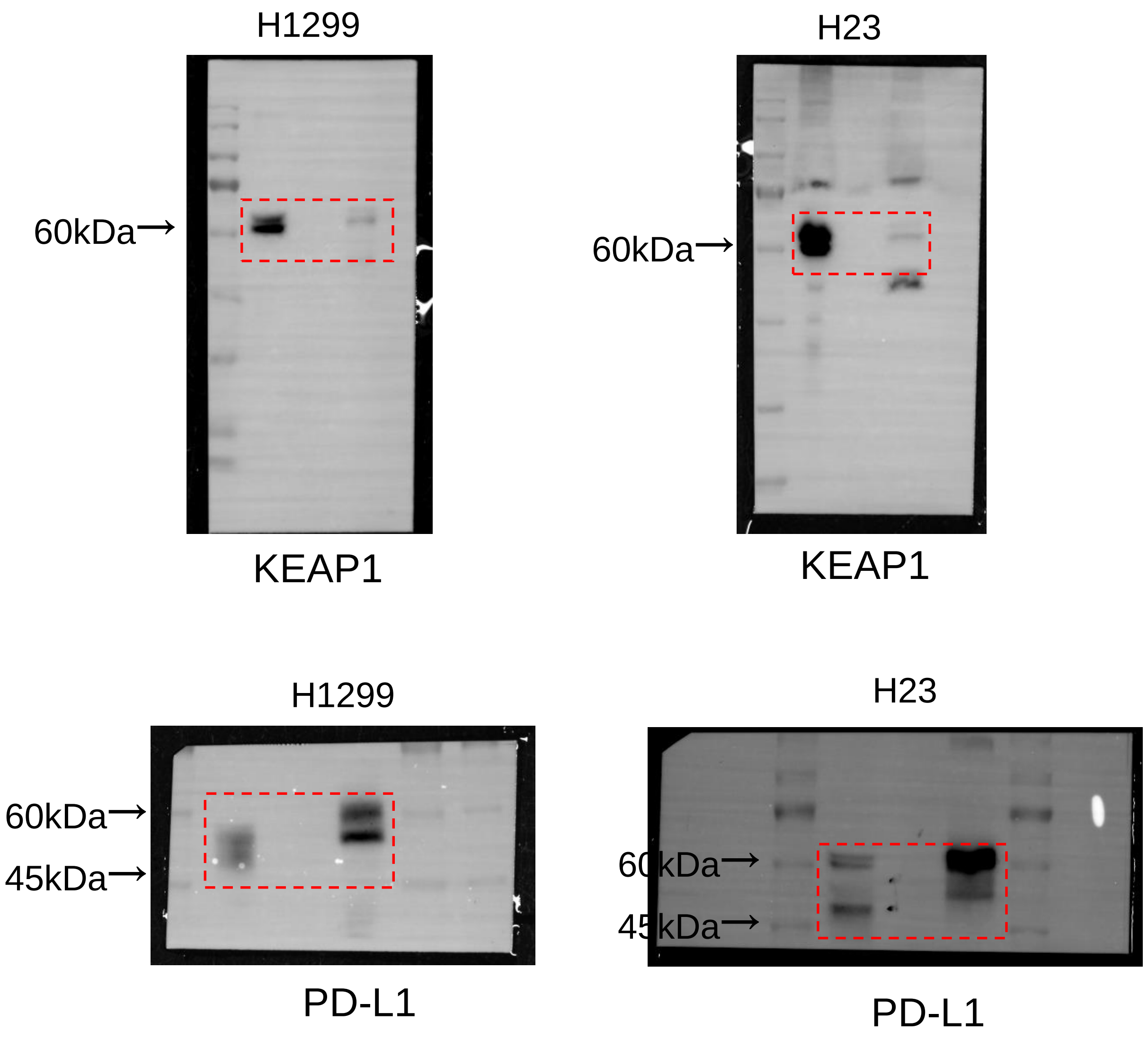

Figure 3D

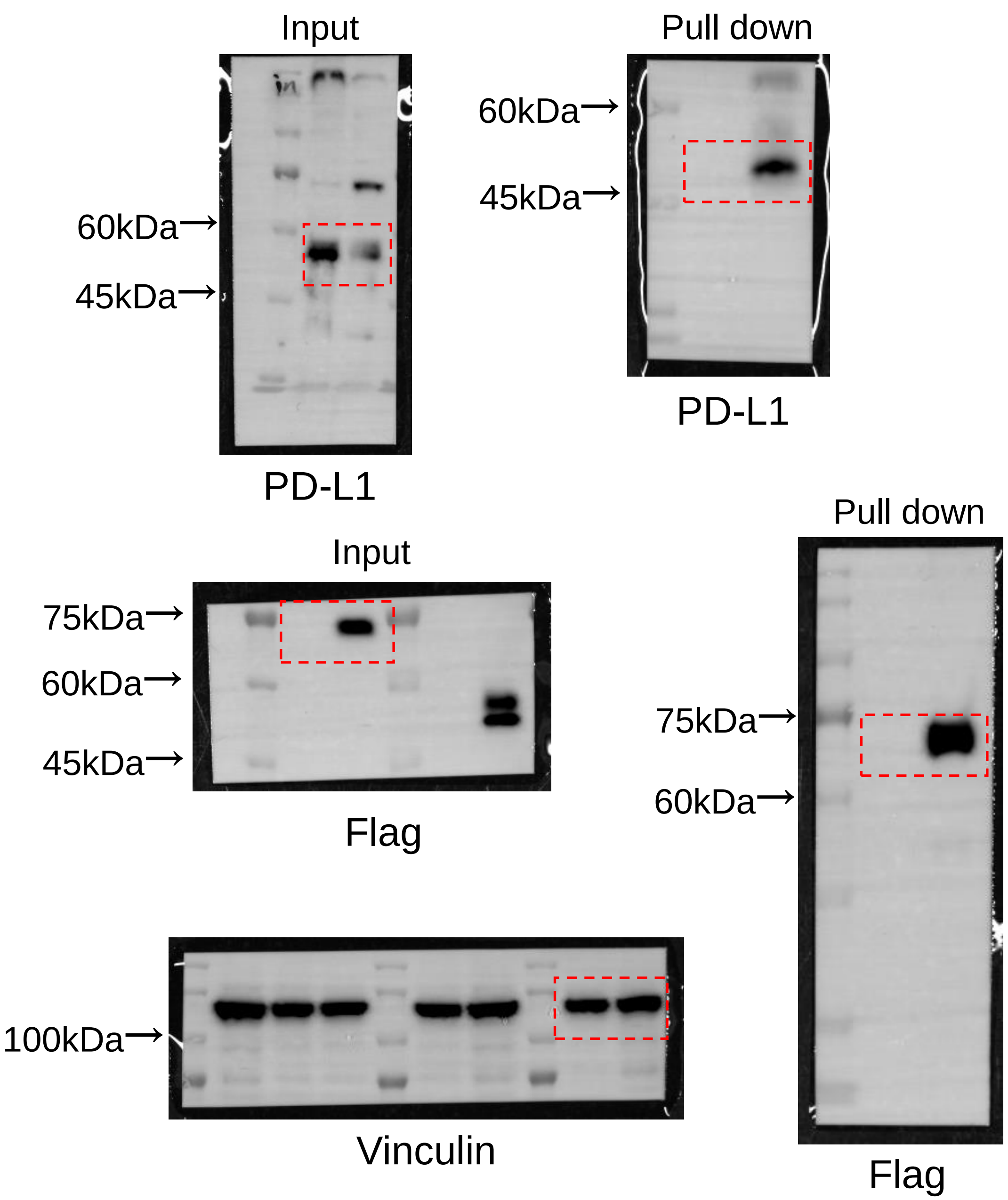

Figure 3E

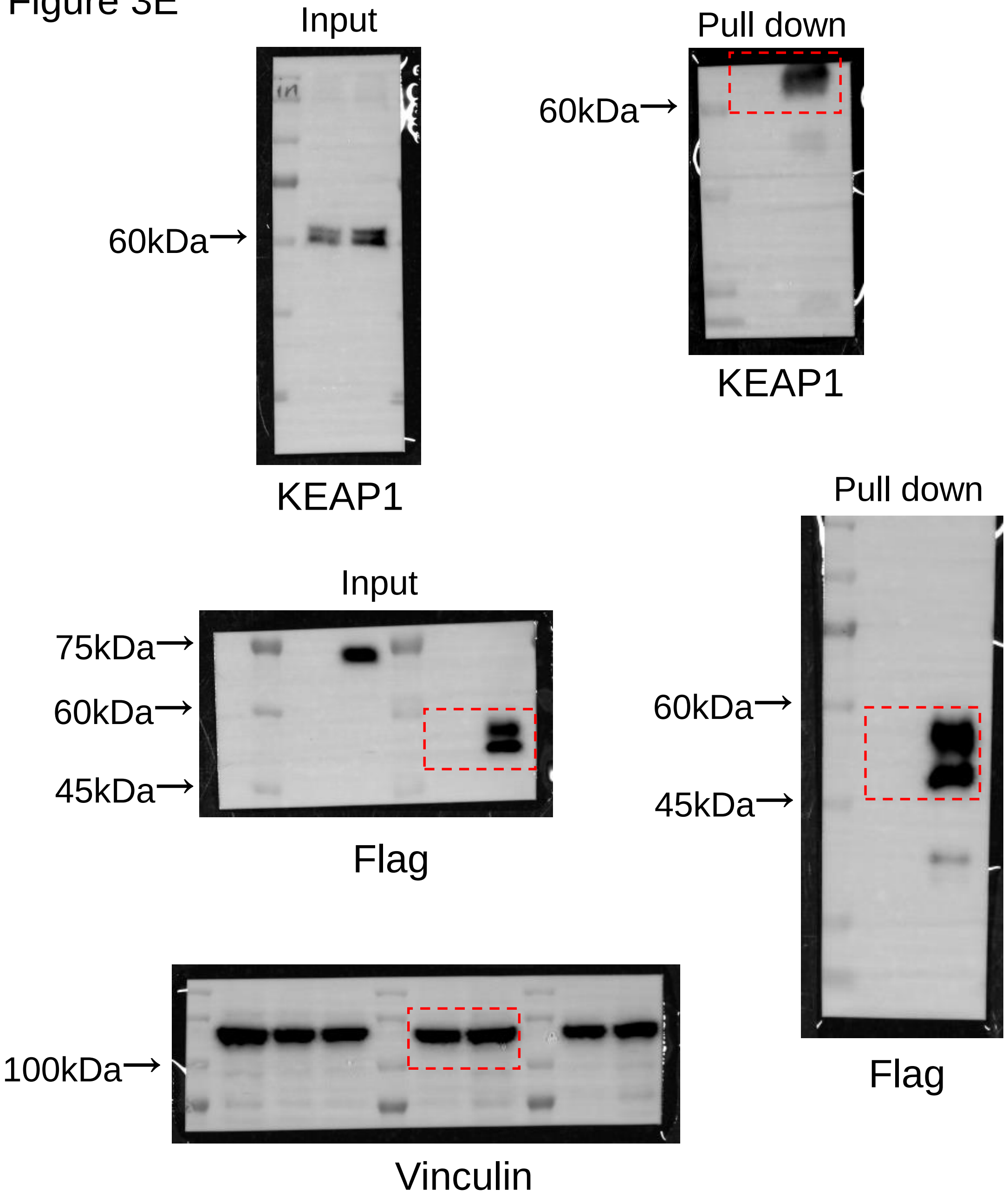

Figure 3F

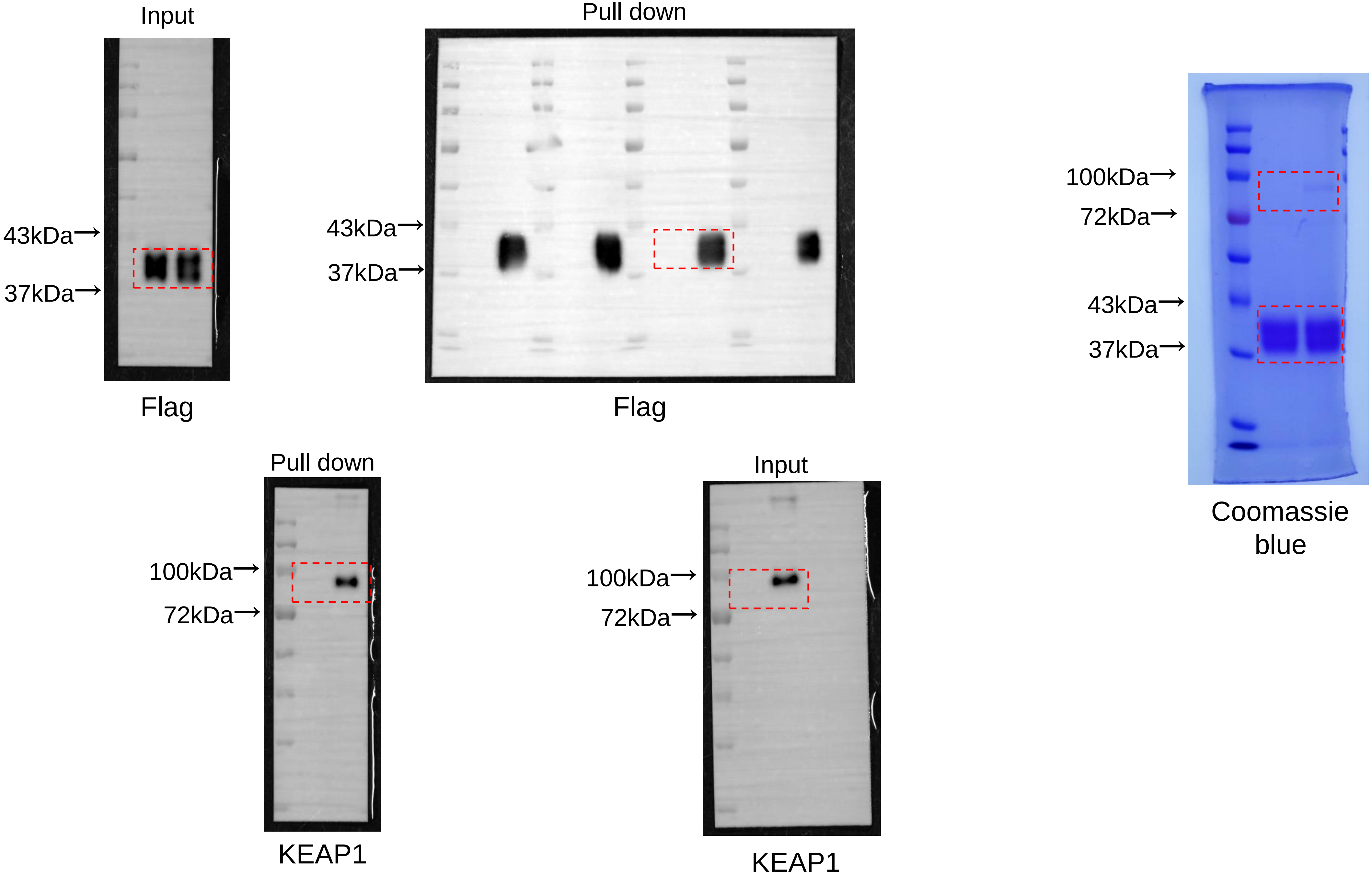

Input
43kDa→
37kDa→
Flag
Pull down
43kDa→
37kDa→
Flag
100kDa→
72kDa→
43kDa→
37kDa→
Coomassie blue
Pull down
100kDa→
72kDa→
KEAP1
Input
100kDa→
72kDa→
KEAP1

Figure 3I

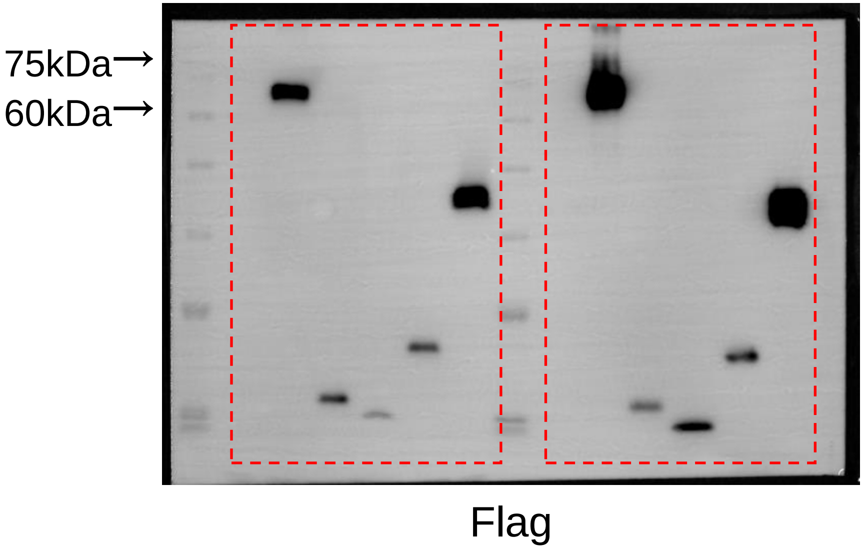
Input
Pull down
75kDa→
60kDa→
Flag

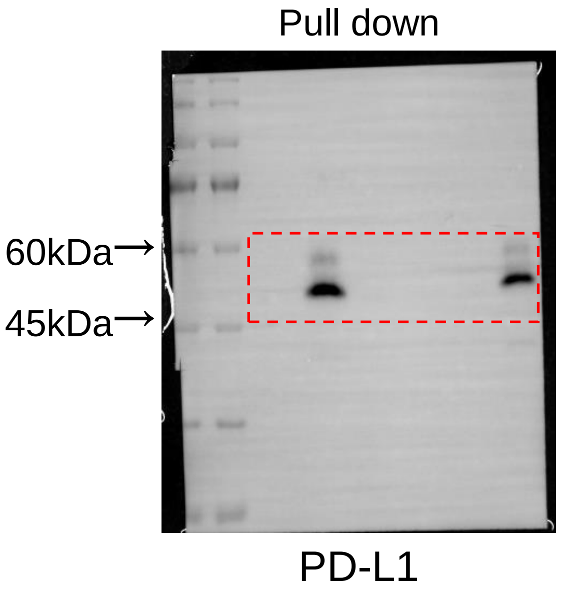
Pull down
60kDa→
45kDa→
PD-L1

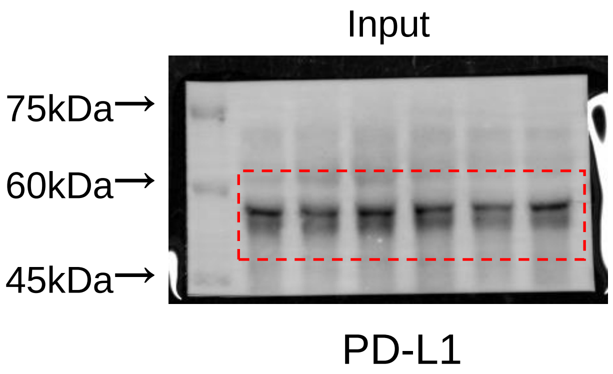
Input
75kDa→
60kDa→
45kDa→
PD-L1

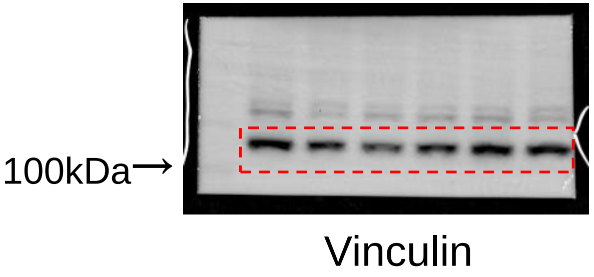
100kDa→
Vinculin

Figure 3J

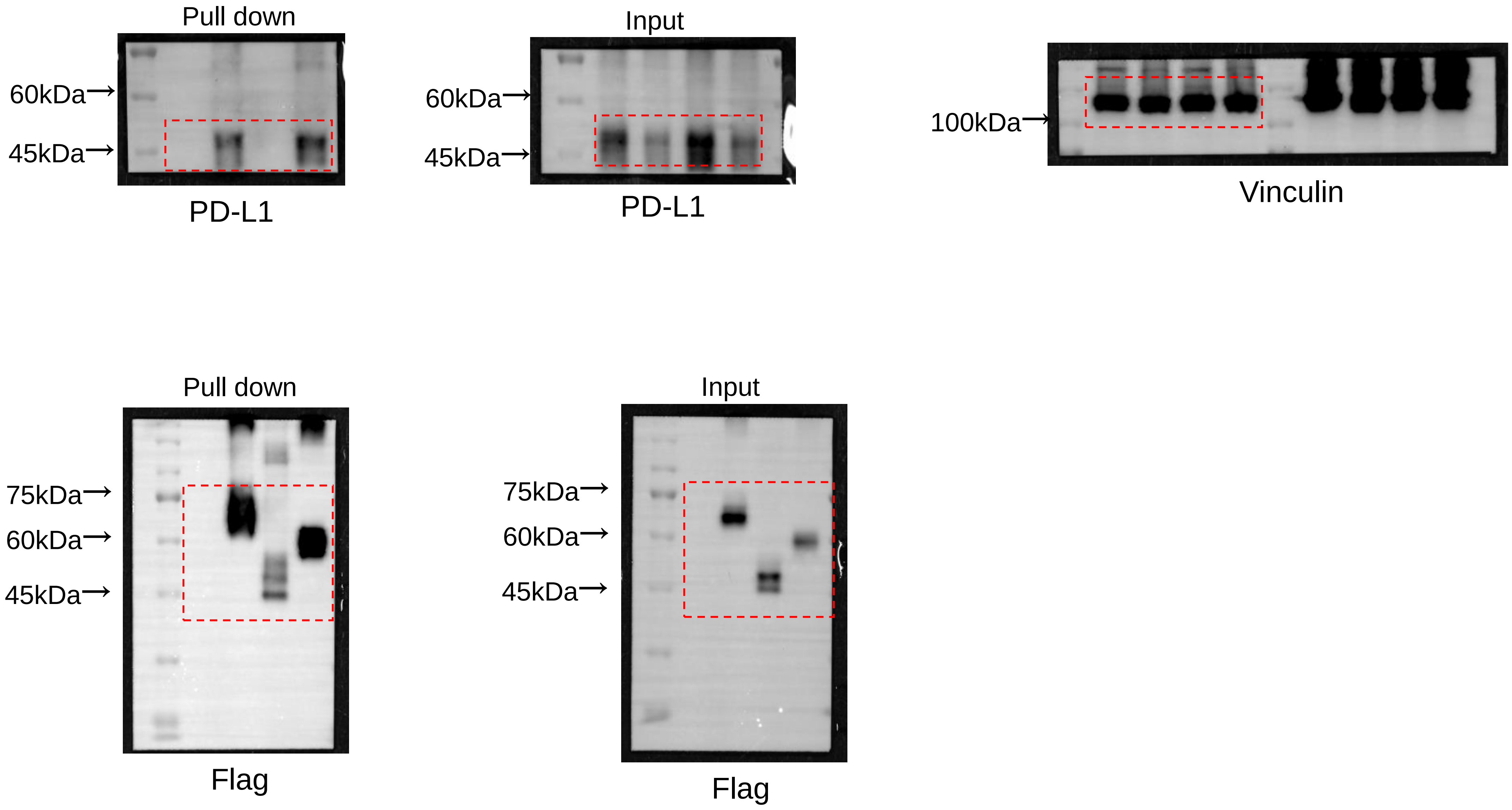

Pull down
60kDa
45kDa
PD-L1
Input
60kDa
45kDa
PD-L1
100kDa
Vinculin
Pull down
75kDa
60kDa
45kDa
Flag
Input
75kDa
60kDa
45kDa
Flag

Figure 4A

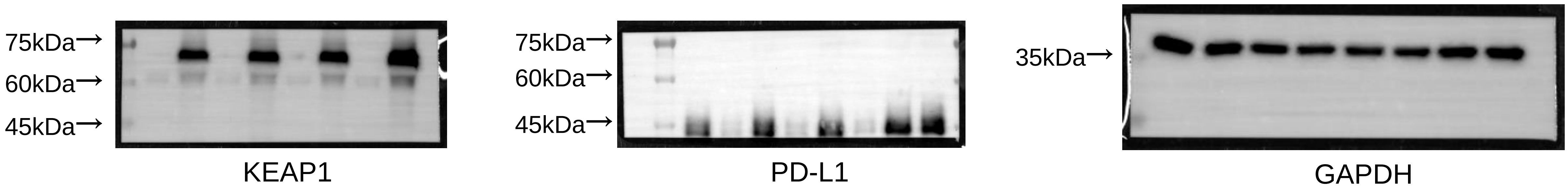

Figure 4B

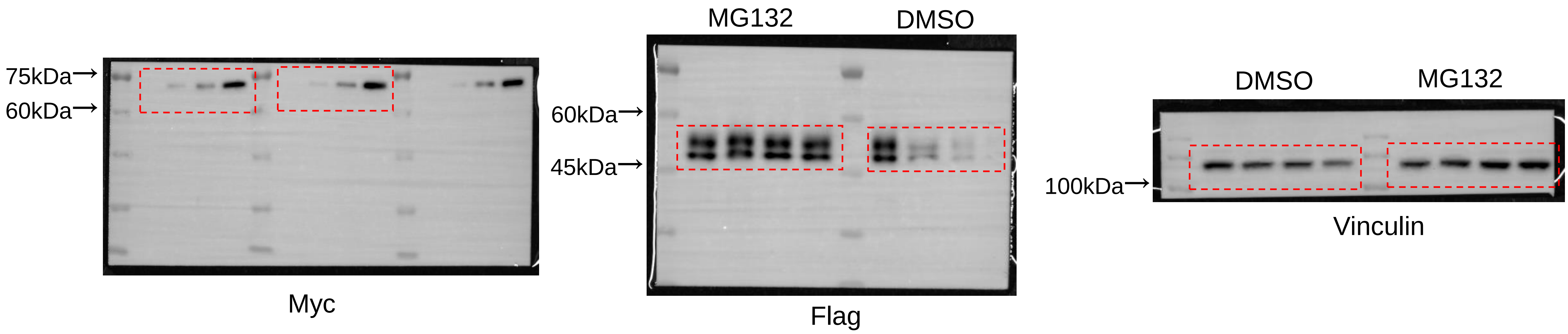

Figure 4C

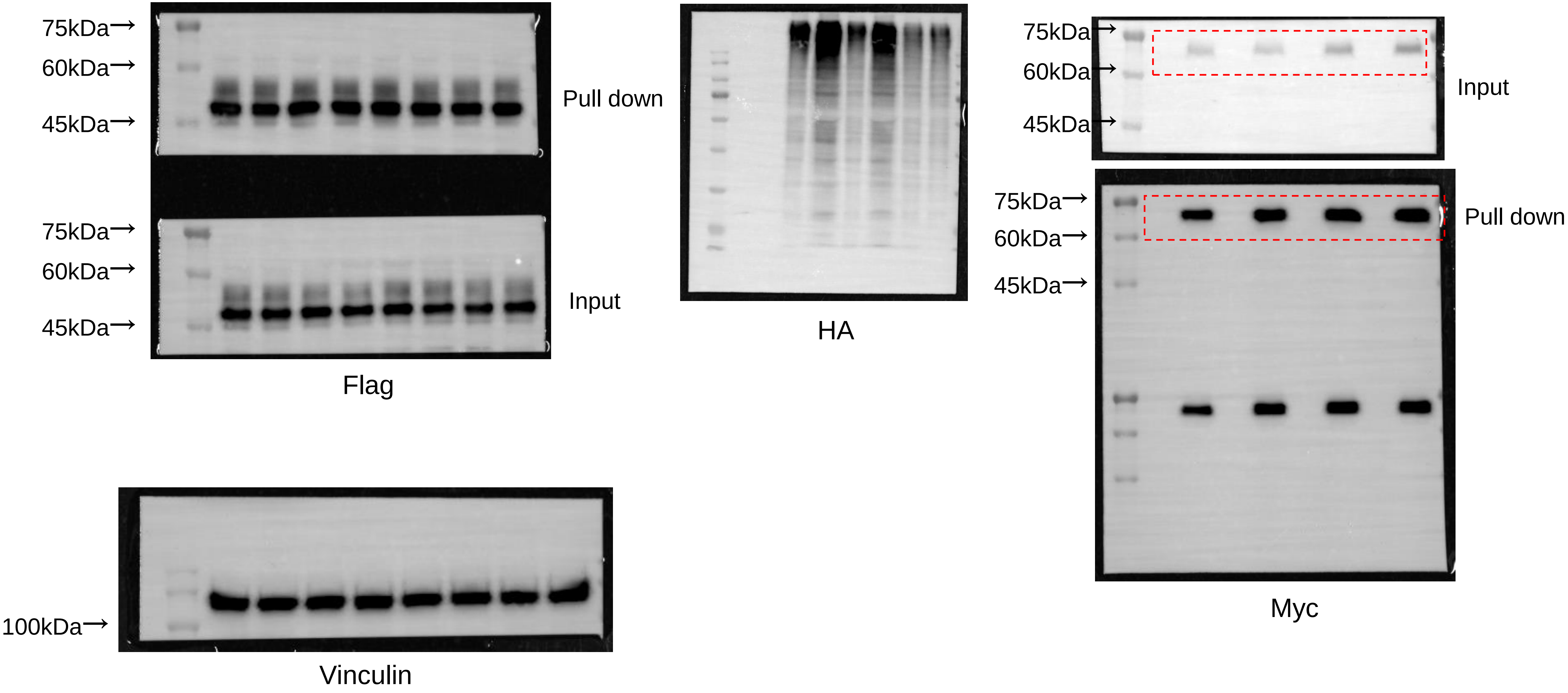
75kDa
60kDa
45kDa
Pull down
75kDa
60kDa
45kDa
Input
Flag
HA
75kDa
60kDa
45kDa
Input
75kDa
60kDa
45kDa
Pull down
Myc
100kDa
Vinculin

Figure 4D

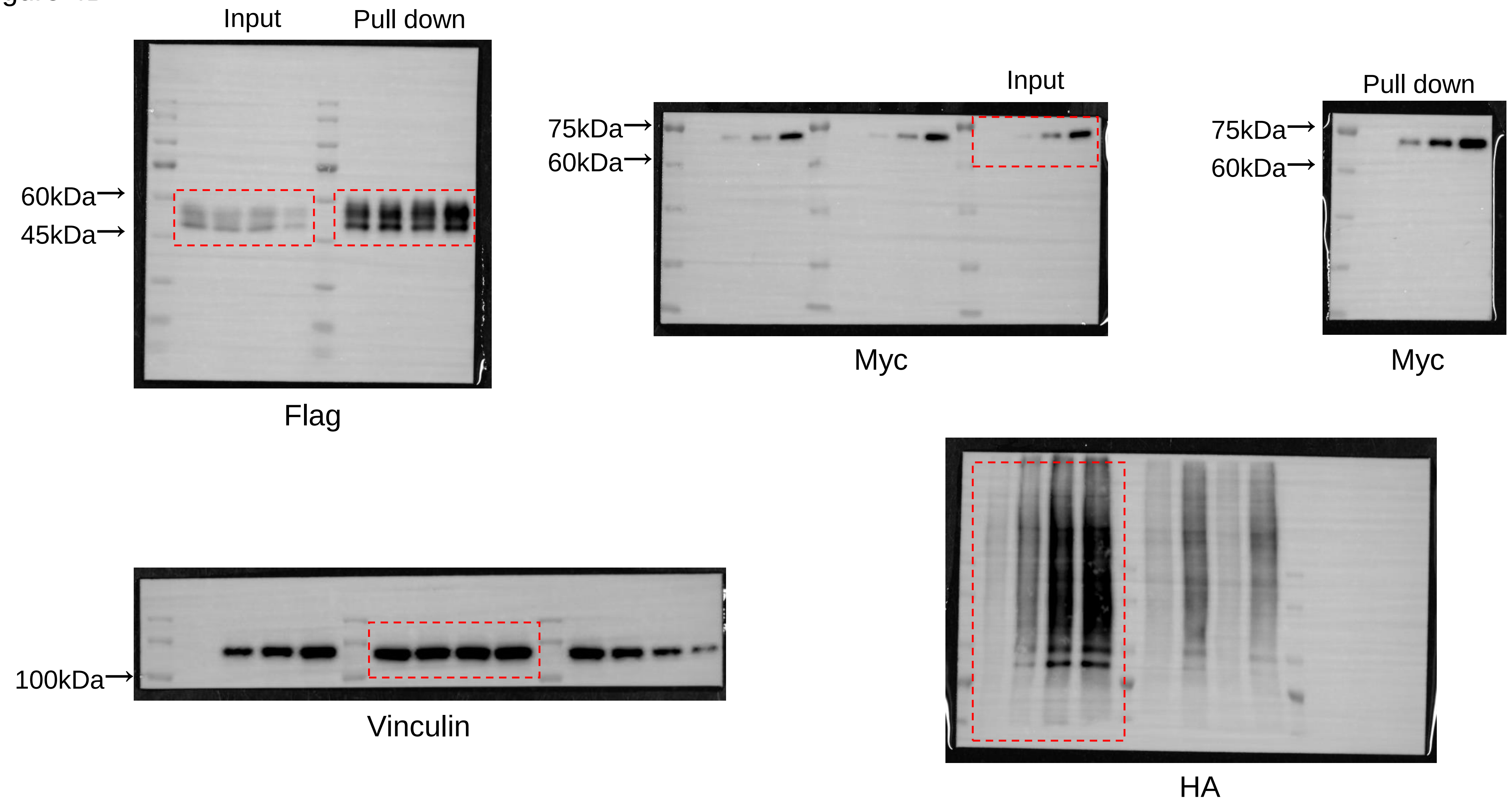
Input
Pull down
60kDa
45kDa
Flag
Input
75kDa
60kDa
Myc
Pull down
75kDa
60kDa
Myc
100kDa
Vinculin
HA

Figure 4E

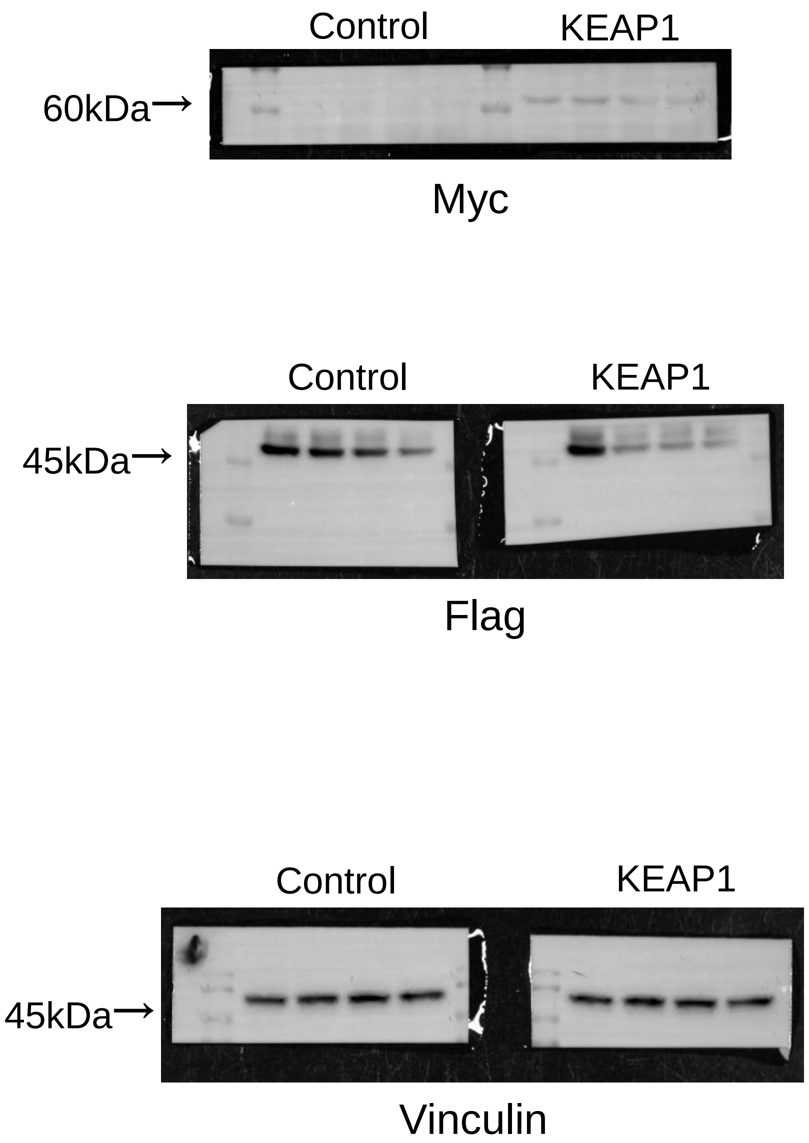

Figure 4G

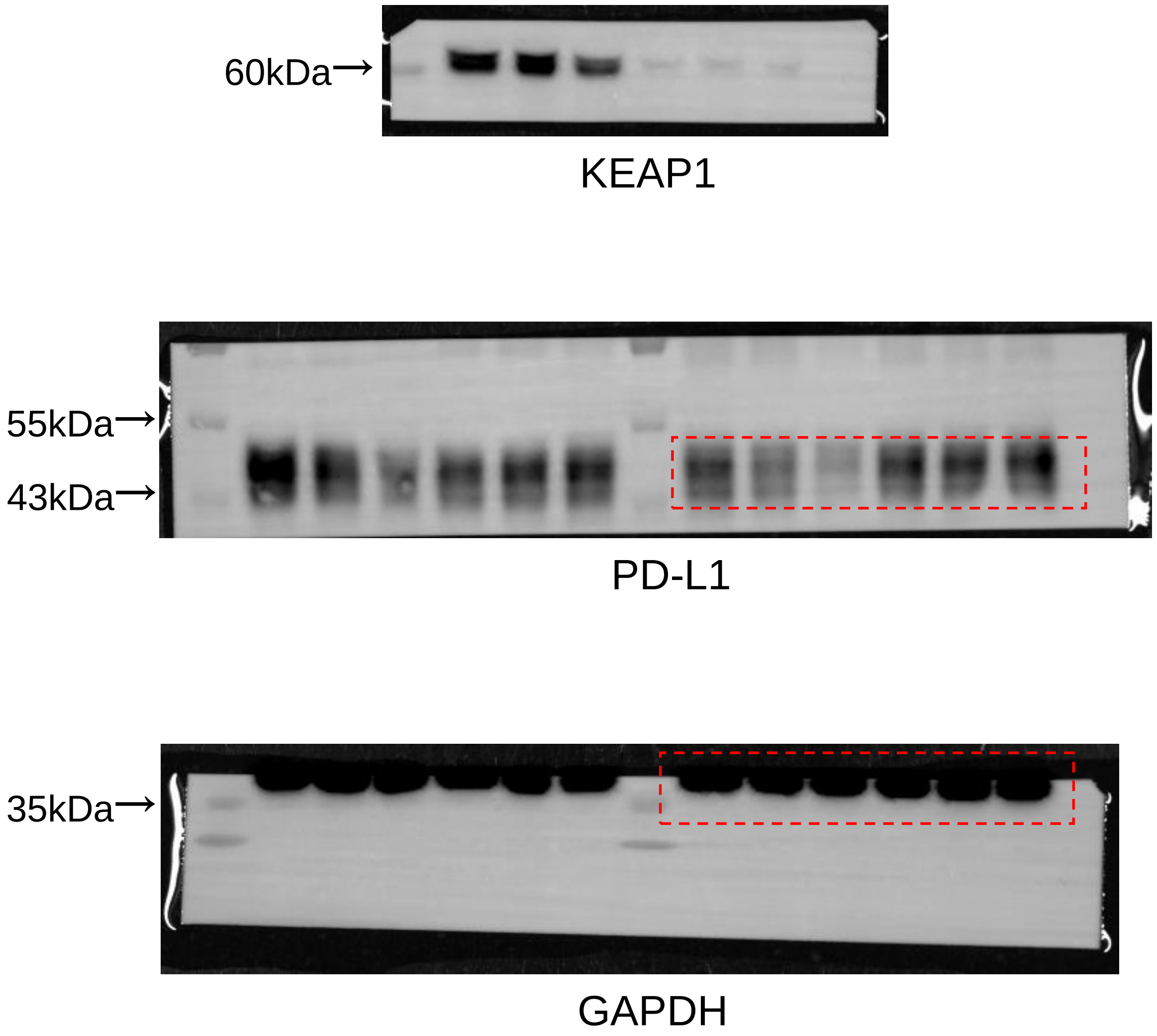

Figure 4I

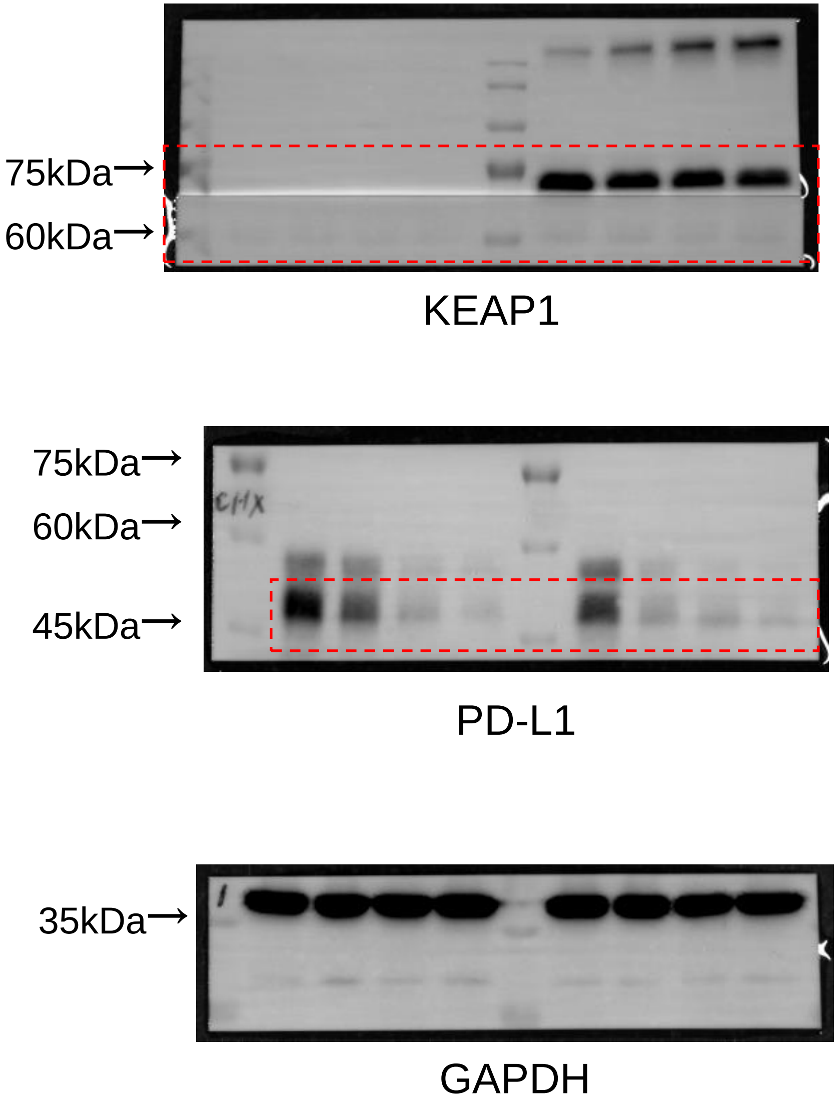

Figure 4K

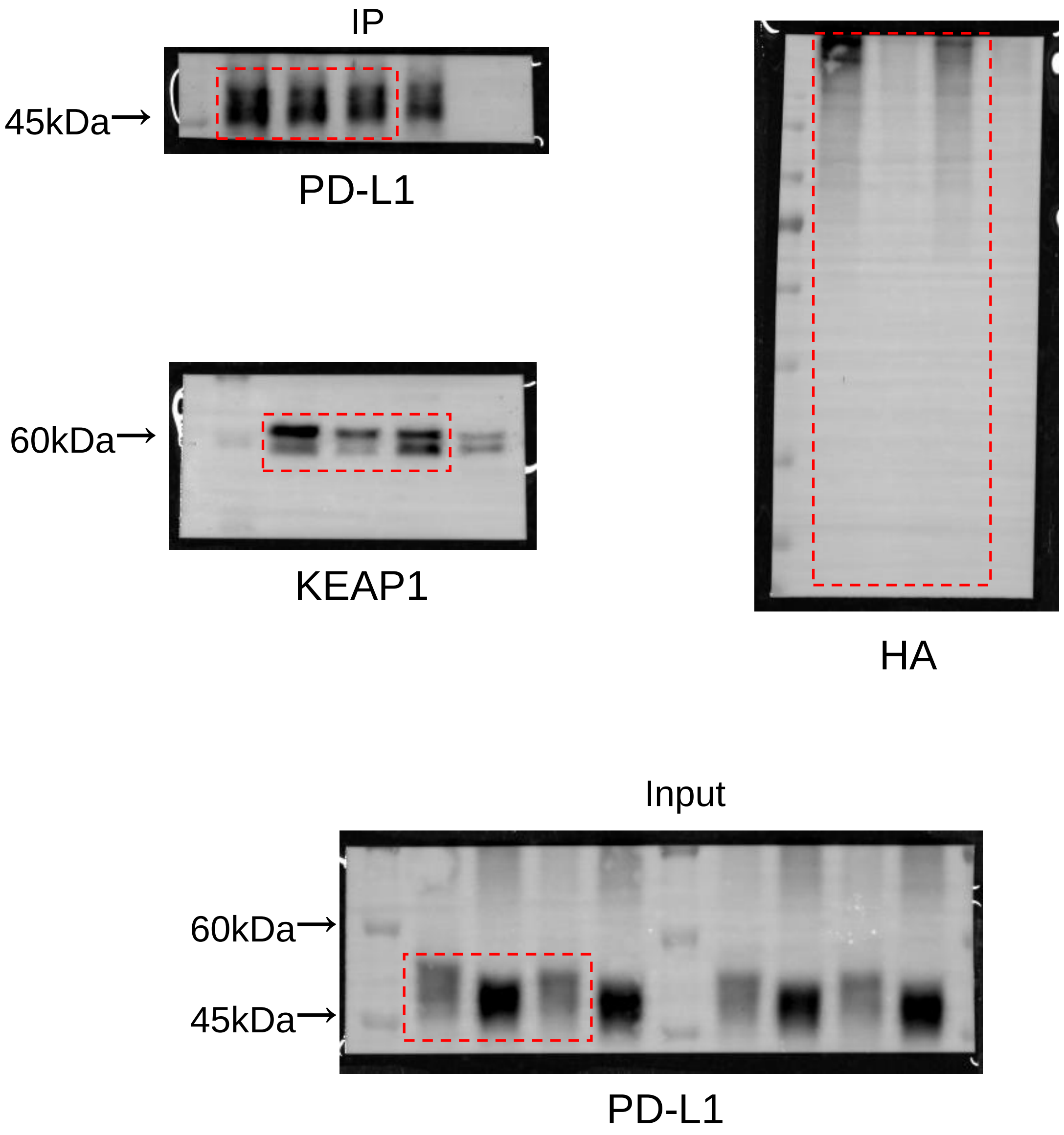

Figure 5A

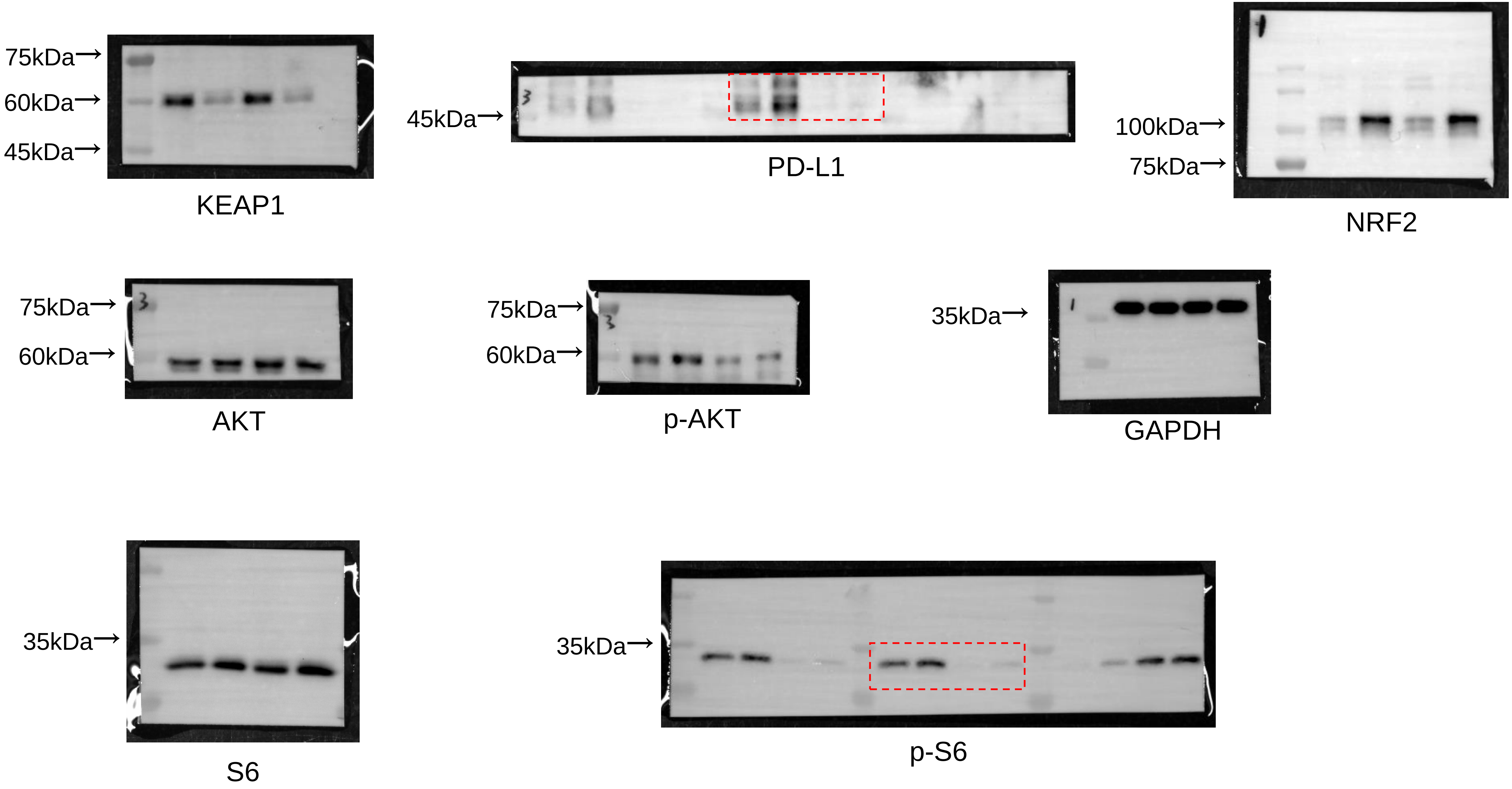
75kDa
60kDa
45kDa
KEAP1
45kDa
PD-L1
100kDa
75kDa
NRF2
75kDa
60kDa
AKT
75kDa
60kDa
p-AKT
35kDa
GAPDH
35kDa
S6
35kDa
p-S6

Figure 6G

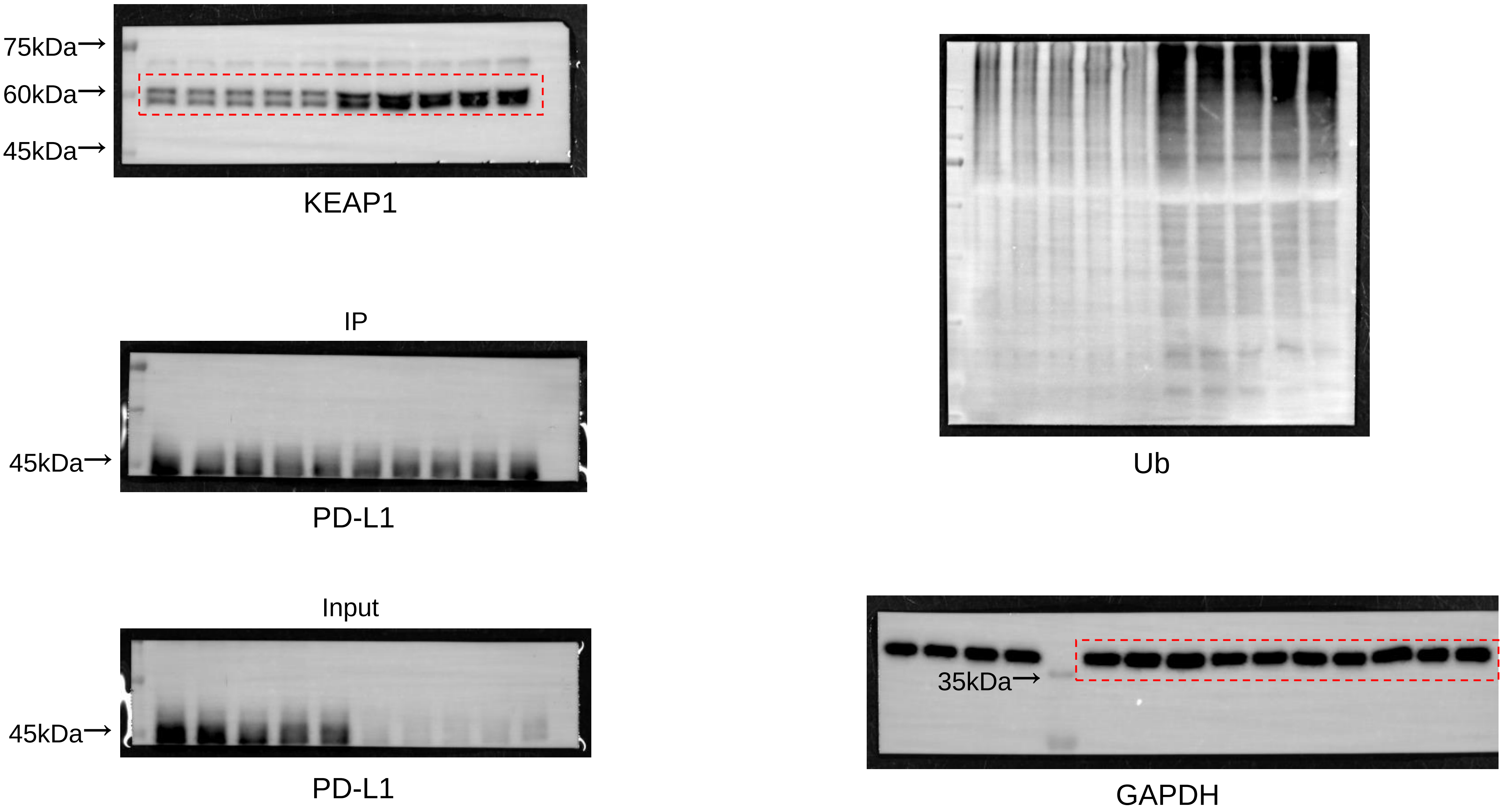
75kDa→
60kDa→
45kDa→
KEAP1
IP
45kDa→
PD-L1
Input
45kDa→
PD-L1
Ub
35kDa→
GAPDH

Figure S2A

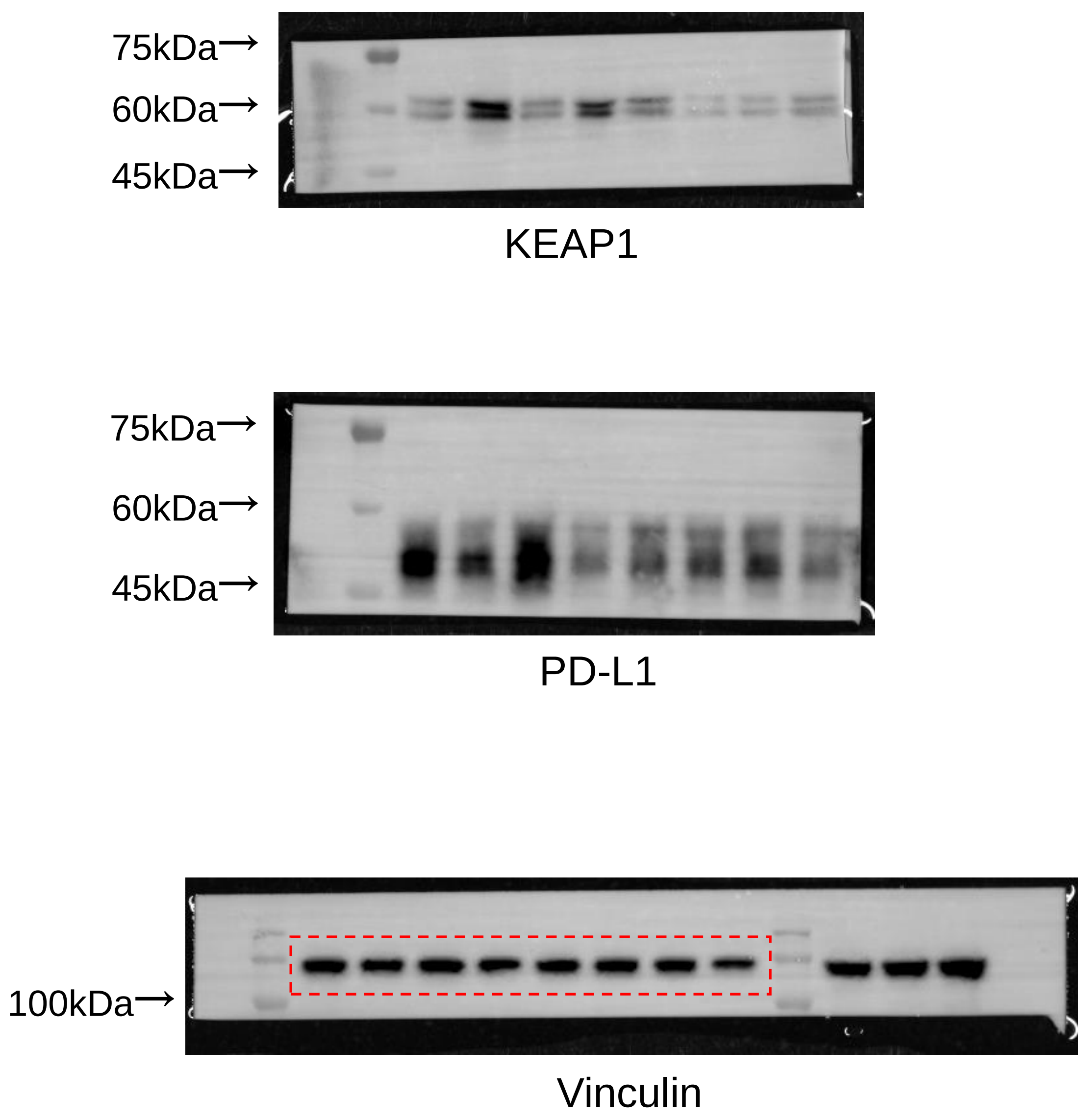

Figure S2B

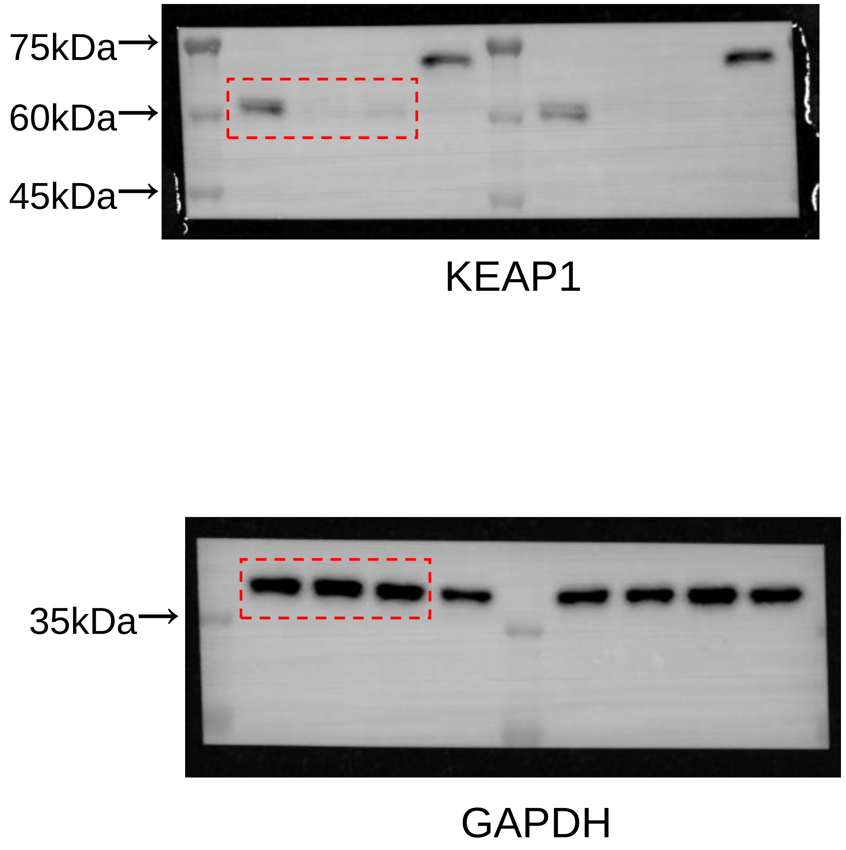

Figure S3C

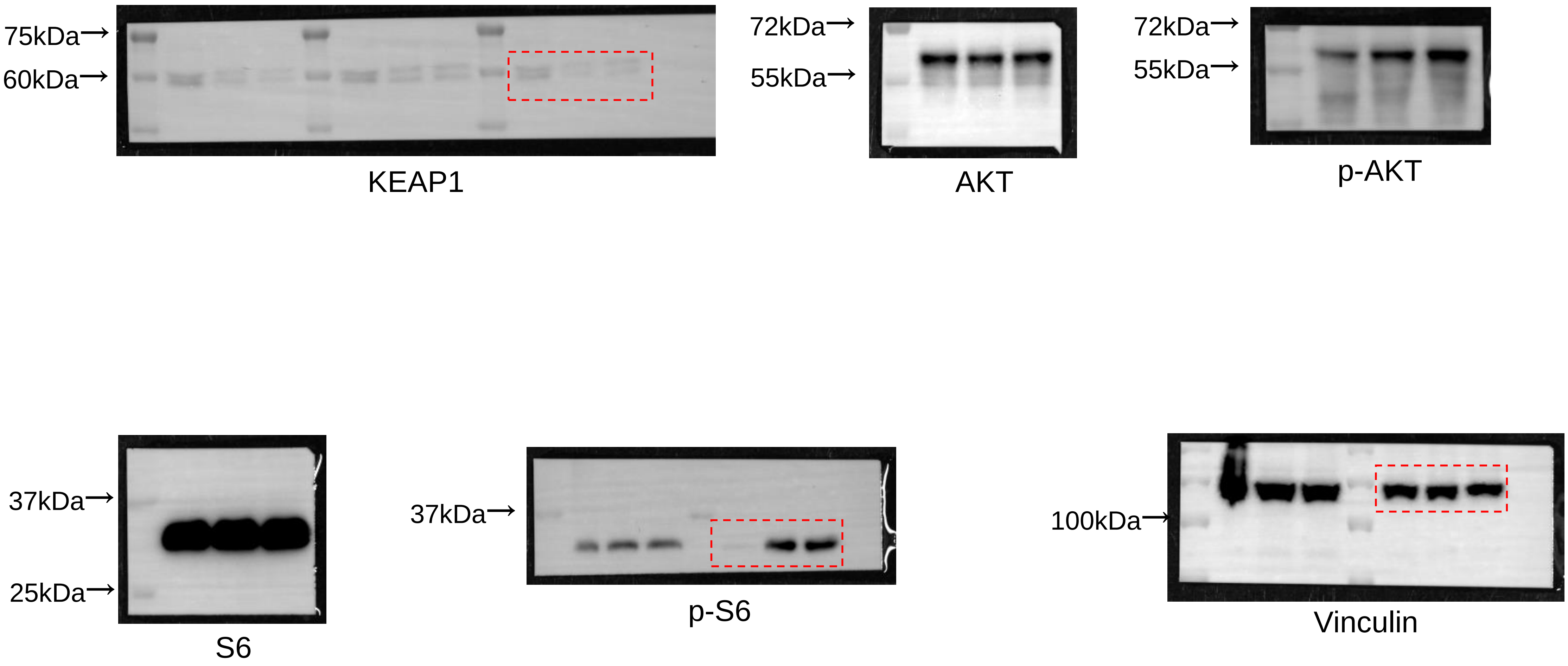
75kDa
60kDa
KEAP1
72kDa
55kDa
AKT
72kDa
55kDa
p-AKT
37kDa
25kDa
S6
37kDa
p-S6
100kDa
Vinculin

Figure S4A

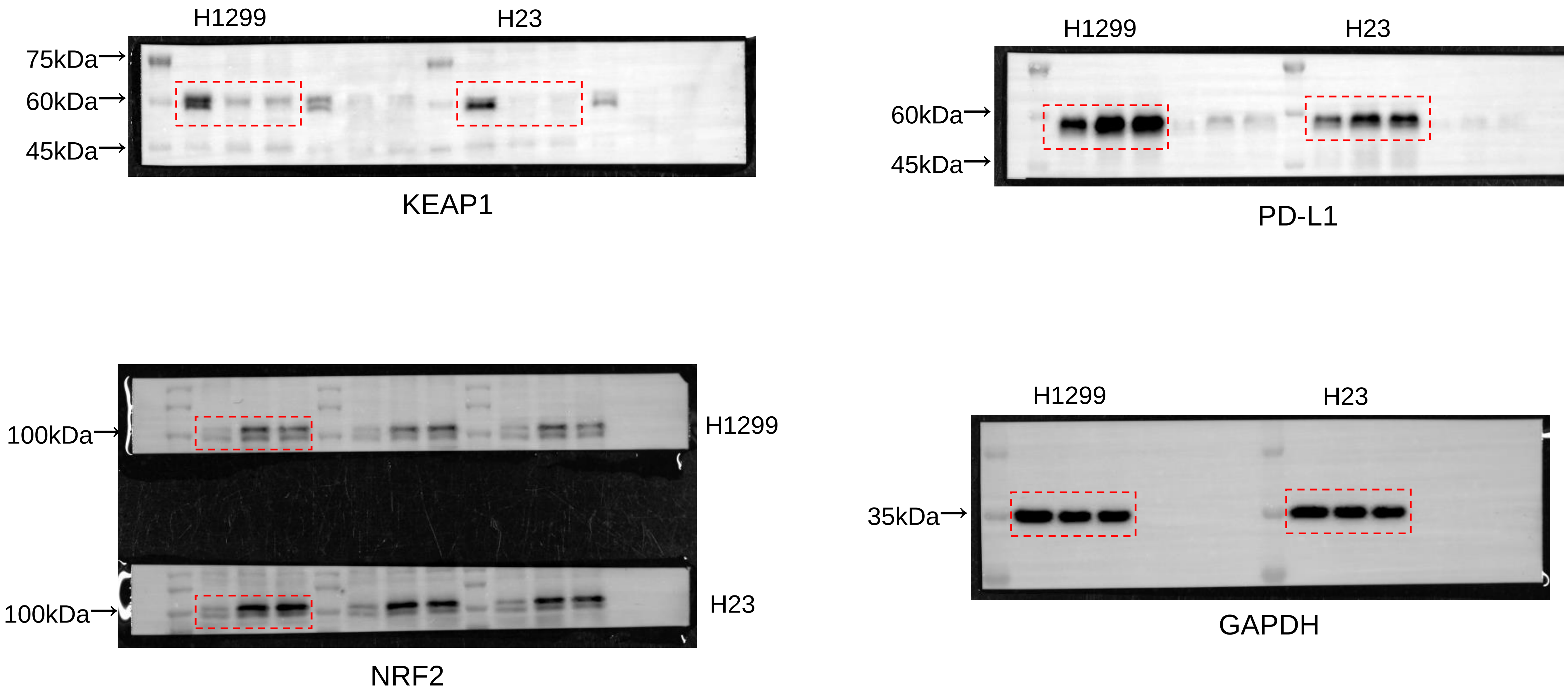
H1299
H23
75kDa
60kDa
45kDa
KEAP1
H1299
H23
60kDa
45kDa
PD-L1
100kDa
H1299
100kDa
H23
NRF2
H1299
H23
35kDa
GAPDH

Figure S4B

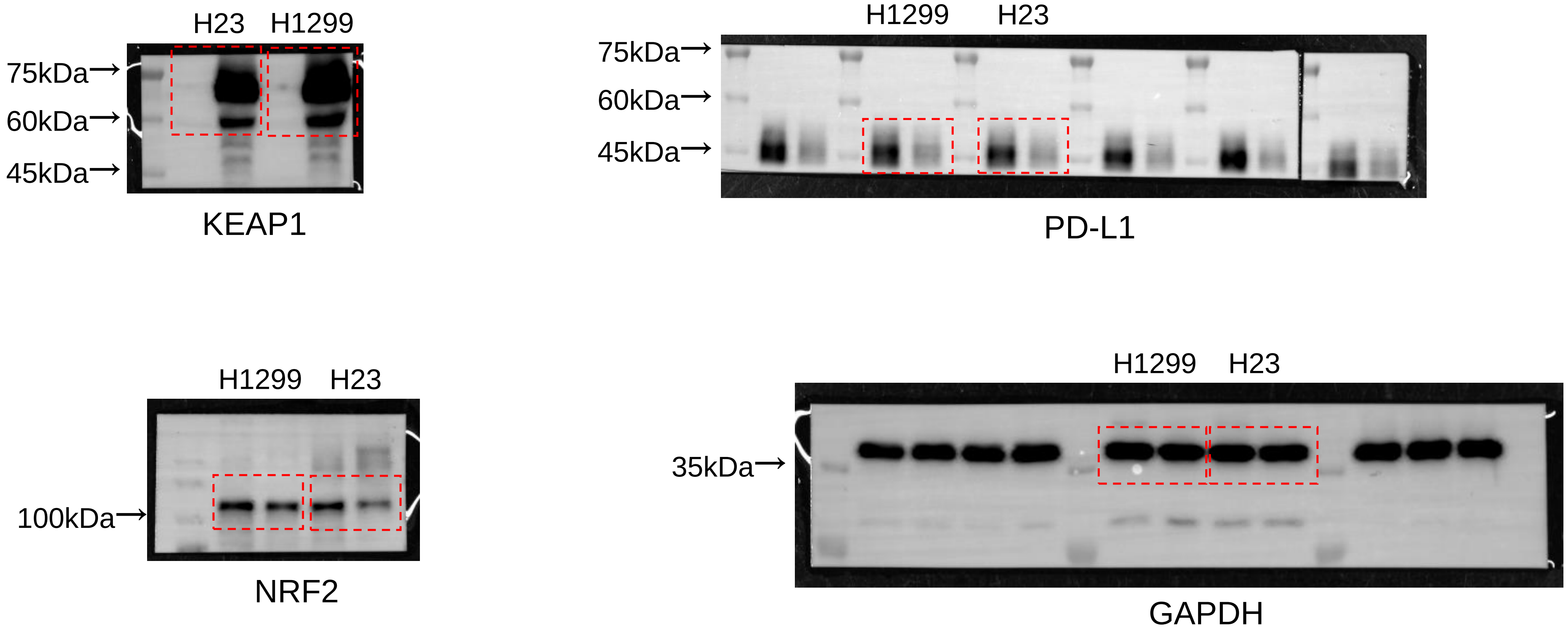

H23
H1299
75kDa→
60kDa→
45kDa→
KEAP1
H1299
H23
75kDa→
60kDa→
45kDa→
PD-L1
H1299
H23
100kDa→
NRF2
H1299
H23
35kDa→
GAPDH

Figure S4C

Well	Fluor	Target	Content	Sample	Cq
A03	SYBR	PD-L1	Unkn	sh2	21.38
A04	SYBR	PD-L1	Unkn	shc	21.19
A05	SYBR	PD-L1	Unkn	sh1	21.91
A08	SYBR	KEAP1	Unkn	sh2	20.11
A09	SYBR	KEAP1	Unkn	shc	18.42
A10	SYBR	KEAP1	Unkn	sh1	20.28
B03	SYBR	PD-L1	Unkn	sh2	21.66
B04	SYBR	PD-L1	Unkn	shc	21.52
B05	SYBR	PD-L1	Unkn	sh1	21.50
B08	SYBR	KEAP1	Unkn	sh2	20.28
B09	SYBR	KEAP1	Unkn	shc	18.03
B10	SYBR	KEAP1	Unkn	sh1	20.10
C03	SYBR	PD-L1	Unkn	sh2	21.27
C04	SYBR	PD-L1	Unkn	shc	21.05
C05	SYBR	PD-L1	Unkn	sh1	21.45
C08	SYBR	KEAP1	Unkn	sh2	20.04
C09	SYBR	KEAP1	Unkn	shc	17.80
C10	SYBR	KEAP1	Unkn	sh1	19.95
D03	SYBR	PD-L1	Unkn	sh2	21.42
D04	SYBR	PD-L1	Unkn	shc	21.57
D05	SYBR	PD-L1	Unkn	sh1	21.55
D08	SYBR	KEAP1	Unkn	sh2	20.43
D09	SYBR	KEAP1	Unkn	shc	18.03
D10	SYBR	KEAP1	Unkn	sh1	20.22
E08	SYBR	GAPDH	Unkn	sh2	15.01
E09	SYBR	GAPDH	Unkn	shc	14.79
E10	SYBR	GAPDH	Unkn	sh1	15.04
F08	SYBR	GAPDH	Unkn	sh2	15.17
F09	SYBR	GAPDH	Unkn	shc	15.07
F10	SYBR	GAPDH	Unkn	sh1	15.33
G08	SYBR	GAPDH	Unkn	sh2	15.05
G09	SYBR	GAPDH	Unkn	shc	14.77
G10	SYBR	GAPDH	Unkn	sh1	15.12
H08	SYBR	GAPDH	Unkn	sh2	15.40
H09	SYBR	GAPDH	Unkn	shc	15.15
H10	SYBR	GAPDH	Unkn	sh1	15.26

shc: shControl

sh1: shKEAP1-1

sh2: shKEAP1-2

Figure S4D

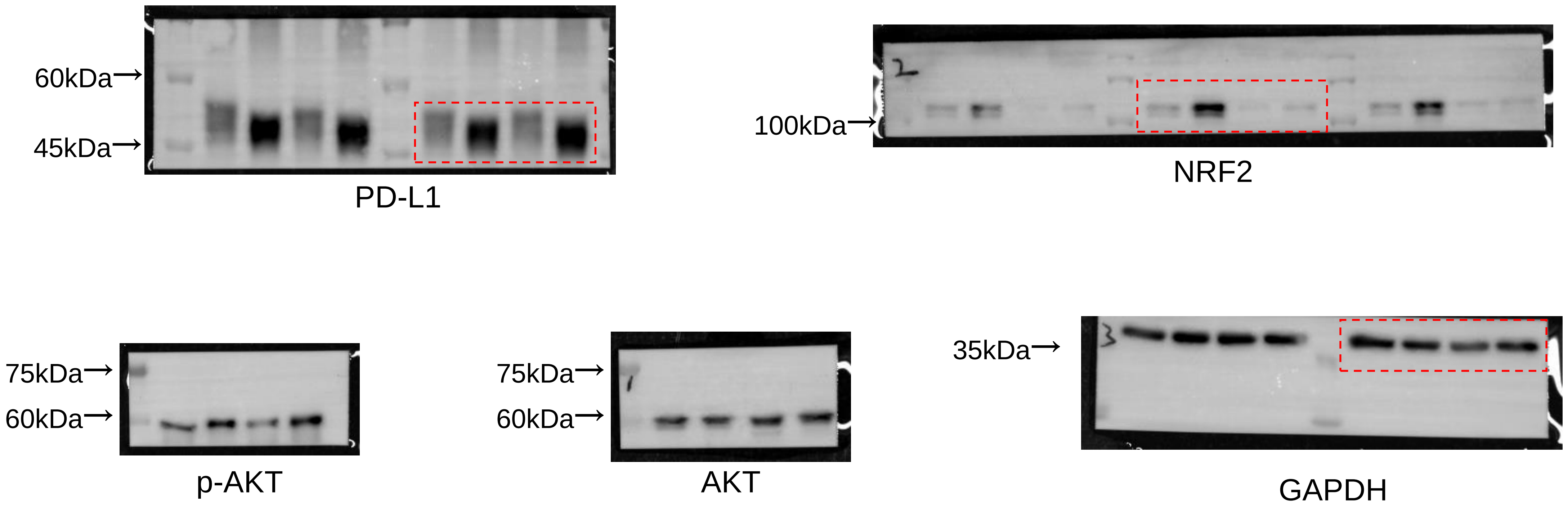
60kDa
45kDa
PD-L1
100kDa
NRF2
75kDa
60kDa
p-AKT
75kDa
60kDa
AKT
35kDa
GAPDH

Figure S5A

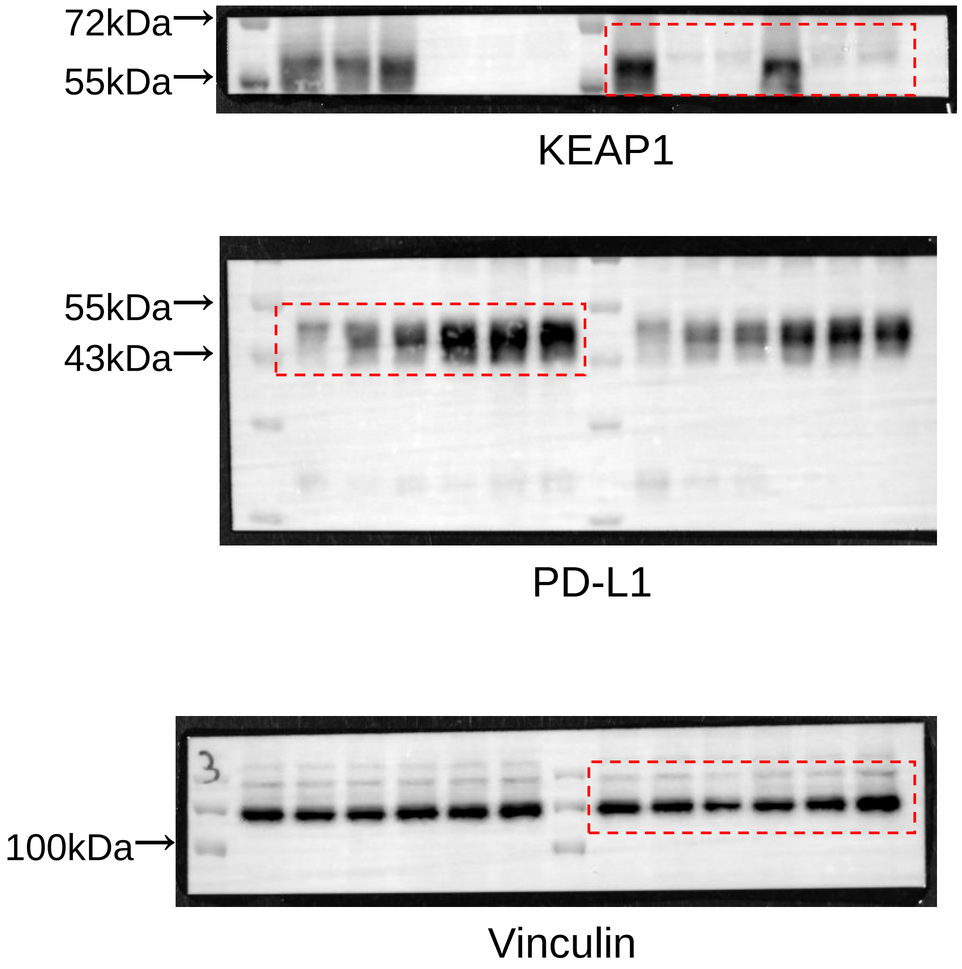

Figure S5B

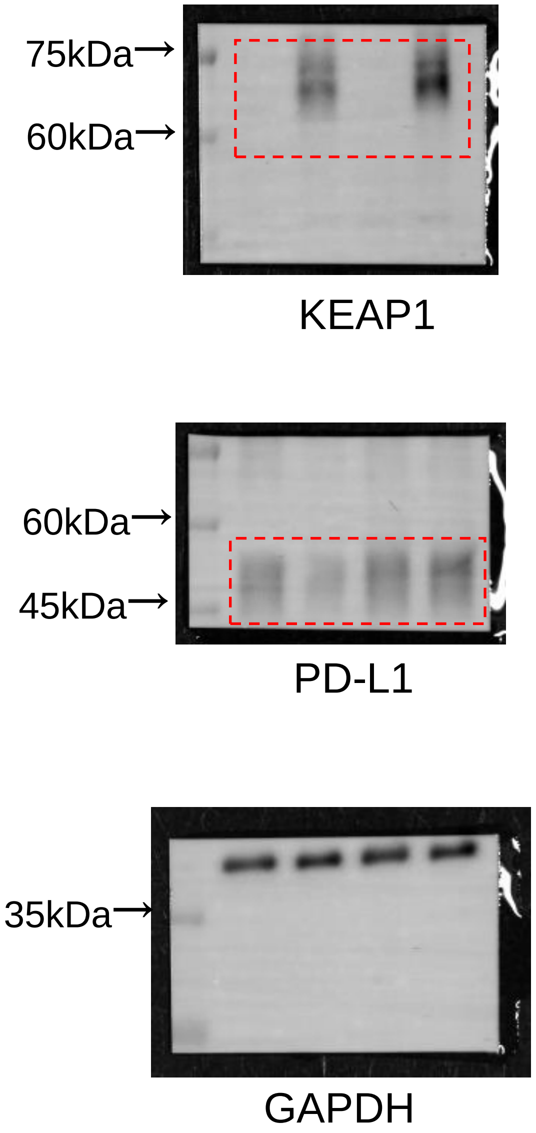

Figure S5C

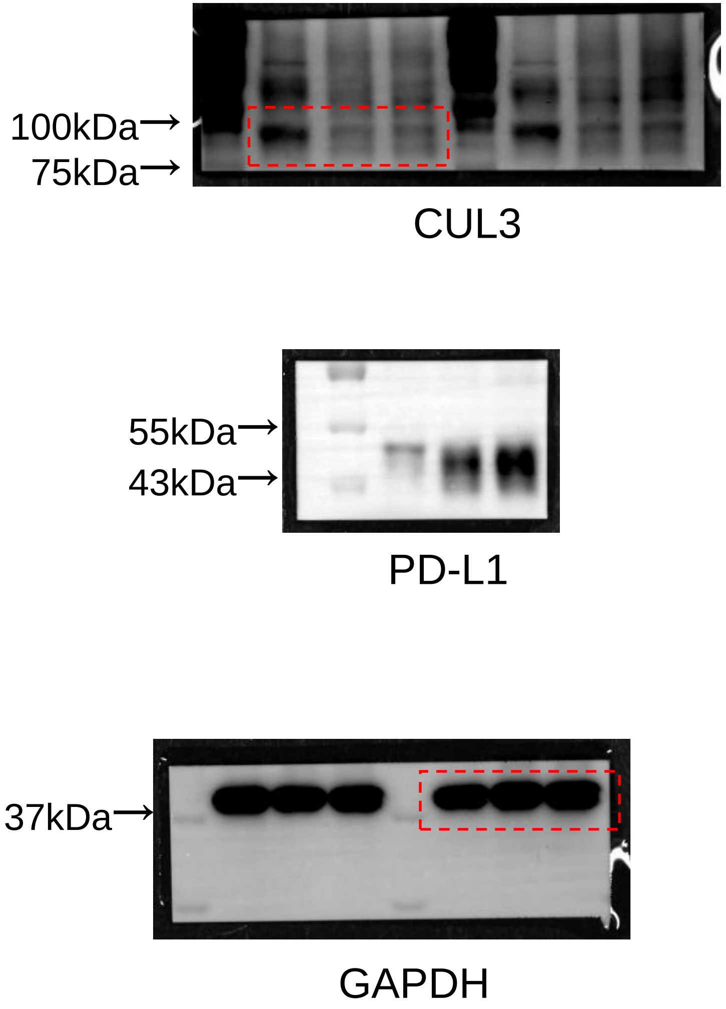

Figure S5D

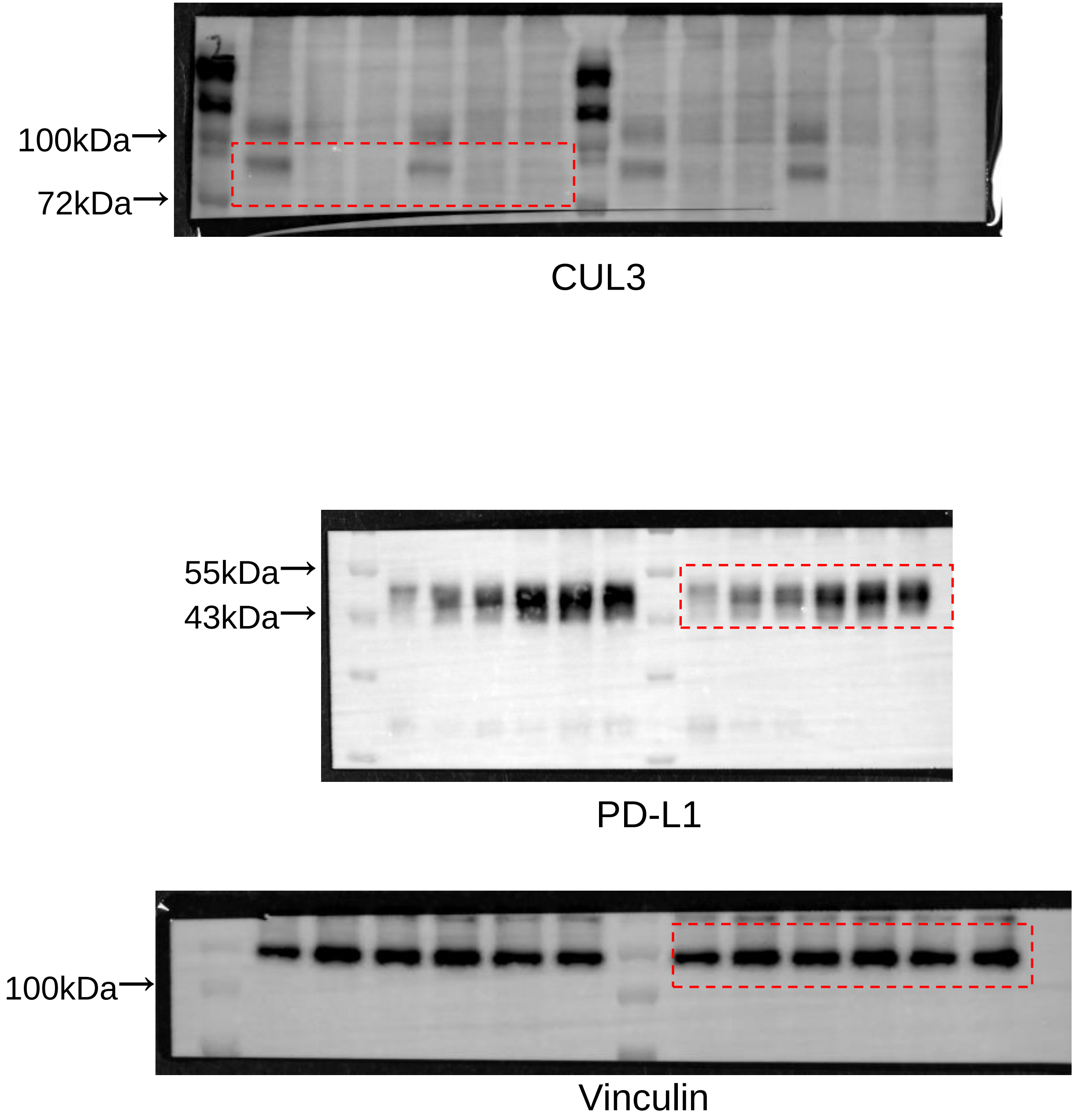

Figure S5E

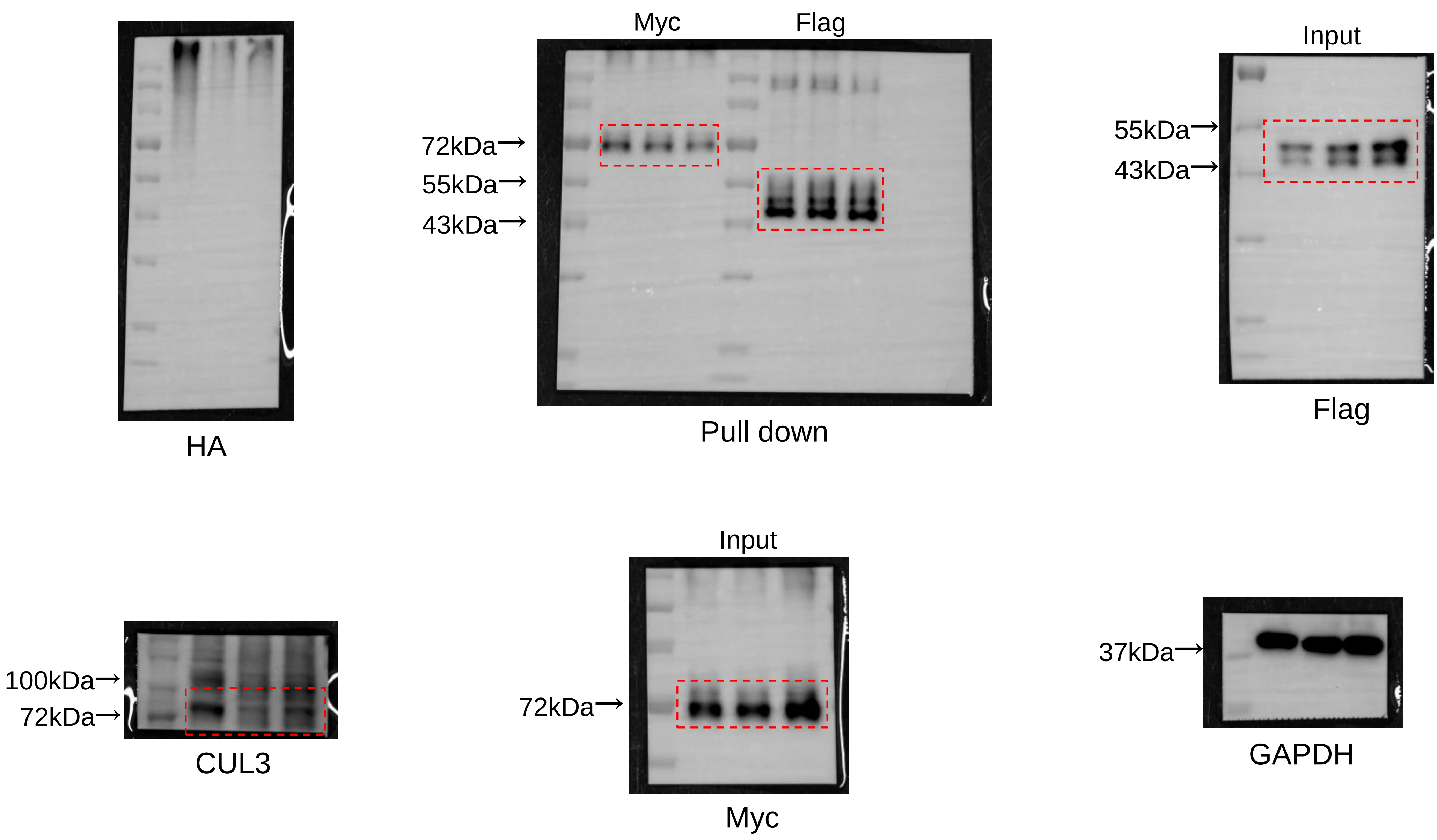
HA
Myc
Flag
72kDa
55kDa
43kDa
Pull down
Input
55kDa
43kDa
Flag
100kDa
72kDa
CUL3
Input
72kDa
Myc
37kDa
GAPDH

Figure S5F

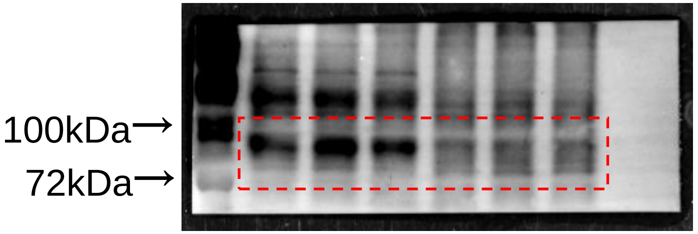

CUL3

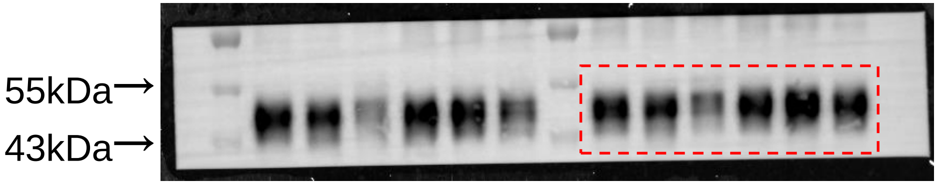

PD-L1

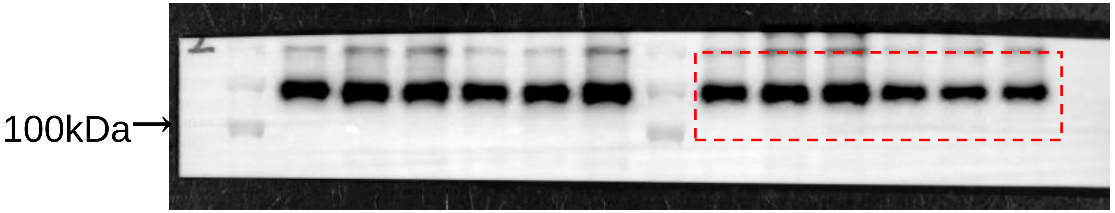

Vinculin

Figure S6A

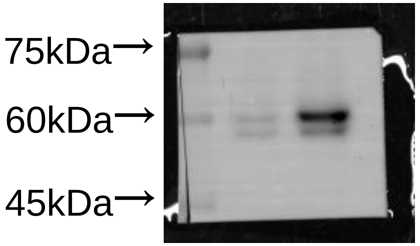

KEAP1

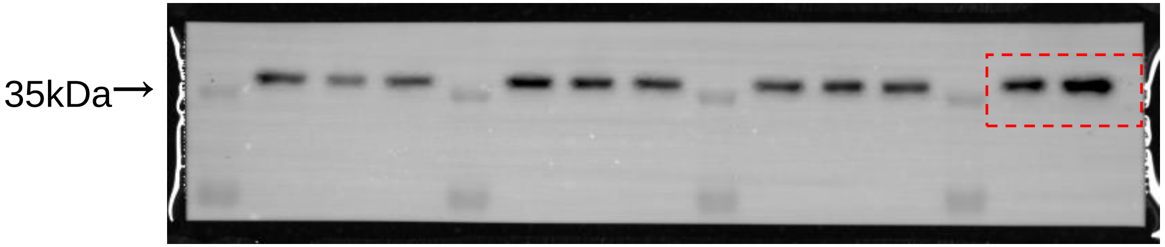

GAPDH

Figure S8A

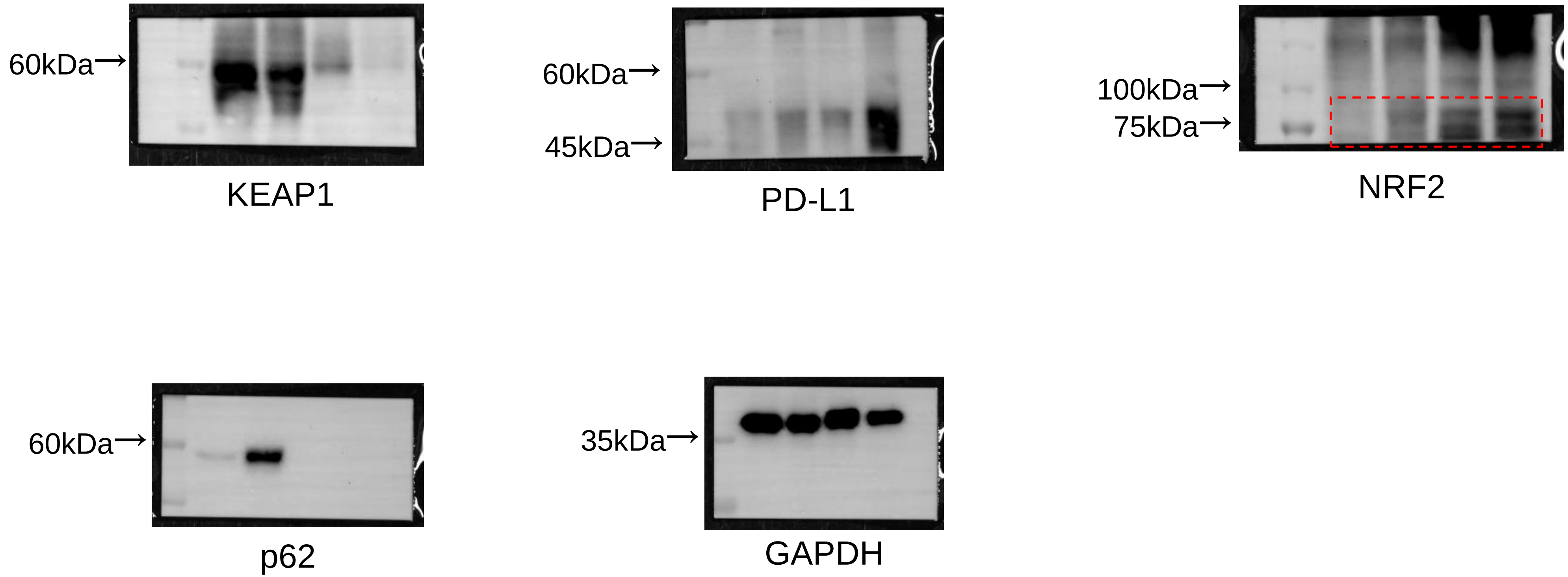
60kDa→
KEAP1
60kDa→
45kDa→
PD-L1
100kDa→
75kDa→
NRF2
60kDa→
p62
35kDa→
GAPDH

Figure S8B

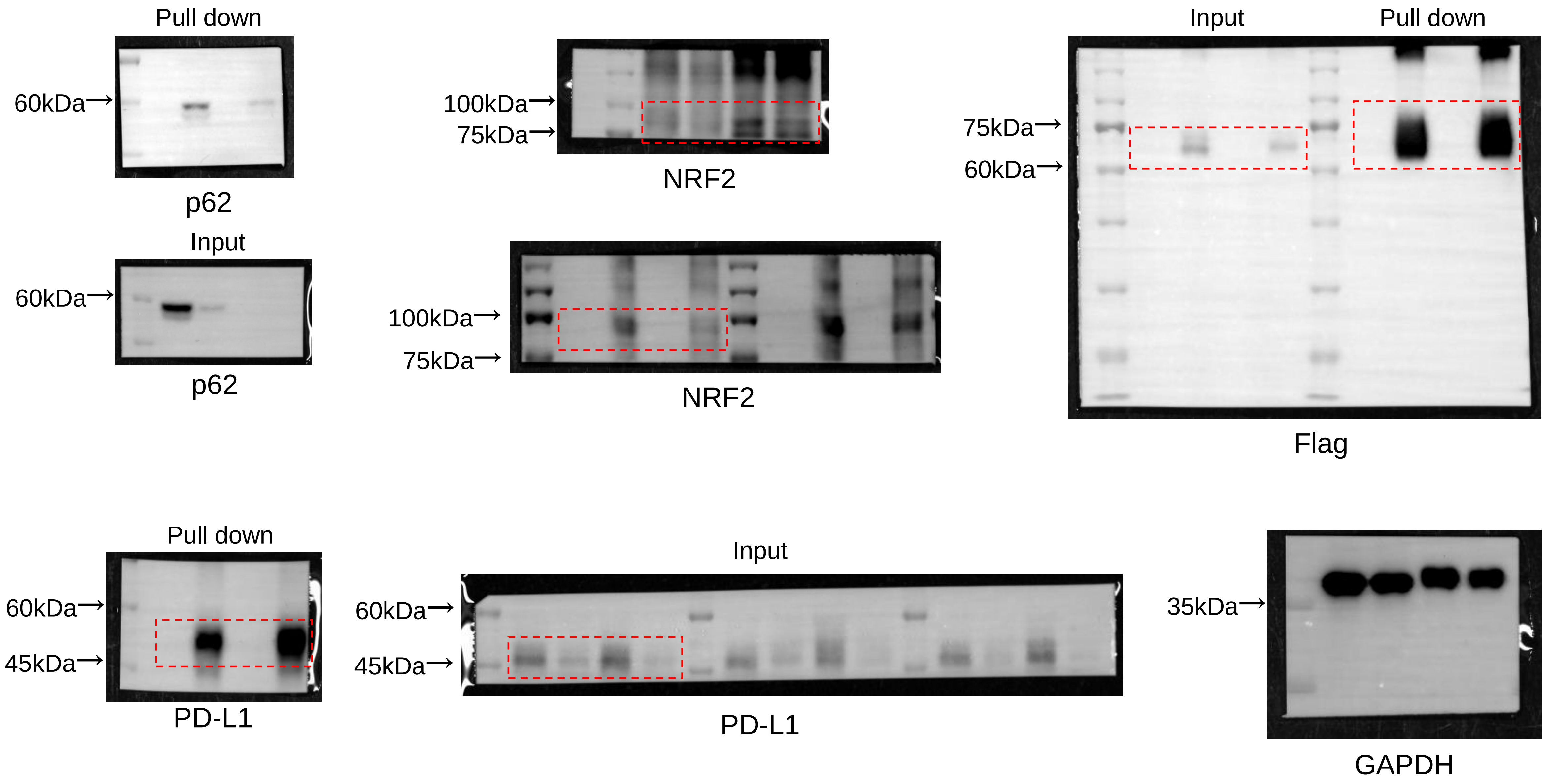
Pull down
60kDa
p62
Input
60kDa
p62
100kDa
75kDa
NRF2
100kDa
75kDa
NRF2
Input
Pull down
75kDa
60kDa
Flag
Pull down
60kDa
45kDa
PD-L1
Input
60kDa
45kDa
PD-L1
35kDa
GAPDH
